# Stylized Facts in Brazilian Vote Distributions

**DOI:** 10.1371/journal.pone.0137732

**Published:** 2015-09-29

**Authors:** Angelo Mondaini Calvão, Nuno Crokidakis, Celia Anteneodo

**Affiliations:** 1 Department of Physics, PUC-Rio, Rio de Janeiro, RJ, Brazil; 2 Institute of Physics, Universidade Federal Fluminense, Niterói, RJ, Brazil; 3 National Institute of Science and Technology for Complex Systems, Rio de Janeiro, RJ, Brazil; Institut Pluridisciplinaire Hubert Curien, FRANCE

## Abstract

Elections, specially in countries such as Brazil, with an electorate of the order of 100 million people, yield large-scale data-sets embodying valuable information on the dynamics through which individuals influence each other and make choices. In this work we perform an extensive analysis of data sets available for Brazilian proportional elections of legislators and city councilors throughout the period 1970–2014, which embraces two distinct political regimes: a military regime followed by a democratic one. We perform a comparative analysis of elections for legislative positions, in different states and years, through the distribution *p*(*v*) of the number of candidates receiving *v* votes. We show the impact of the different political regimes on the vote distributions. Although *p*(*v*) has a common shape, with a scaling behavior, quantitative details change over time and from one electorate to another. In order to interpret the observed features, we propose a multi-species model consisting in a system of nonlinear differential equations, with values of the parameters that reflect the heterogeneity of candidates. In its simplest setting, the model can not explain the cutoff, formed by the most voted candidates, whose success is determined mainly by their peculiar, intrinsic characteristics, such as previous publicity. However, the modeling allows to interpret the scaling of *p*(*v*), yielding a predictor of the degree of feedback in the interactions of the electorate. Knowledge of the feedback is relevant beyond the context of elections, since a similar interactivity may occur for other social contagion processes in the same population.

## Introduction

In the last few decades, the study of elections has sparked special attention amongst statistical physicists [1–6]. An election can be seen as a large scale event, that provides a huge amount of real data about people choices, resulting from collective processes. Then, its analysis may reveal important hints on how people are influenced and opinions propagate. The amount of data is particularly huge in countries such as Brazil, with a large and diversified electorate. Moreover, differently from the case of presidential or governor elections, with few candidates, in an election for legislators or for city councilors, there is a large pool of candidates among which a voter can choose. This provides a wide spectrum of choices that allows to probe how people preferences distribute [7–9].

Plausibly following these motivations, pioneering works analyzed the Brazilian proportional elections of 1998 [10–14], for federal and state deputies in the most populated states (São Paulo and Minas Gerais). It was first claimed that the probability density function (PDF) of the number of votes *p*(*v*) for deputies exhibited a common feature: a power law decay *p*(*v*) ∼ 1/*v* ^*α*^, with exponent *α* = 1 [10–16]. Diverse opinion dynamics models were then proposed to explain such behavior [11–13, 17]. The simple Sznajd dynamics (only agreeing pairs of individuals can convince their neighbors in the network of contacts [4]) appeared to be enough to explain the power-law exponent *α* = 1. A robust result, almost independent of the network properties [11, 12]. A simple contagion model [17] was also able to reproduce the 1/*v* behavior, but the small-world effect appeared to be crucial in that case.

Nevertheless, for Brazilian city councilors, the exponent was found to be larger than unit [14]. Elections with proportional rules in other countries, like India [13], Finland [15], Italy [18], and France [19], have also been analyzed revealing that the power-law exponent, or even the power-law itself, was not robust [18]. As a consequence, it was argued that an essential feature to capture a universal behavior was to take into account the role of parties [15, 18, 20]. In fact, it is sound that a voter chooses first a party, following its ideology, and then the voter chooses a candidate belonging to that party. Moreover, voters may be influenced by the fact that the total amount of votes for a party will decide the corresponding number of seats. However, at least for Brazilian proportional elections, several features hamper a meaningful normalization by party: i) There are currently over 30 political parties, distributed in a broad spectrum of orientations [21], but parties occasionally merge together or split, and there are also alliances, in some cases of mixed political orientation. ii) There are no electoral thresholds to disqualify parties with low representativity. iii) Because the vote is compulsory, many people who are not interested in politics participate. iv) Although there is the possibility of voting on a party (*legenda* vote), instead of the nominal vote on a candidate, for those interested in supporting a party, most people vote mainly for candidates directly (within an open list system). Then, electoral choices largely rely on the personal characteristics of candidates [22]. Moreover, we will analyze exclusively nominal votes. For all these reasons, we will disregard aspects related to parties. By scrutinizing *p*(*v*), we aim to gain insights on the formation of preferences of the electorate about candidates only.

We analyze Brazilian elections for legislators over the period of 1970–2014, for which data are available at the website of the Brazilian Federal Electoral Court [23]. This period encompasses elections that took place during the civilian governments after 1986, as well as during the precedent military dictatorship [24], allowing to investigate the impact of very different political regimes on vote distributions. Complementarily, we also consider the elections for city councilors between 2000 and 2012.

A detailed description of the electoral context is provided in the *Materials and methods* section. In the section *Vote distributions*, we show illustrative cases of the analysis of empirical data, and the stylized facts of *p*(*v*), based on the Supporting Information (SI) material. In section *Model*, we introduce a mathematical model inspired in multi-species population dynamics that provides a possible interpretation of the empirical shape of the distributions. Notice that the scenario for legislative elections we analyze is different from the one of presidential elections involving a few candidates (a binary choice in USA [5]) and hence requires a distinct modeling. A discussion is presented in the last section.

## Results

### Vote distributions

**Fig 1:**
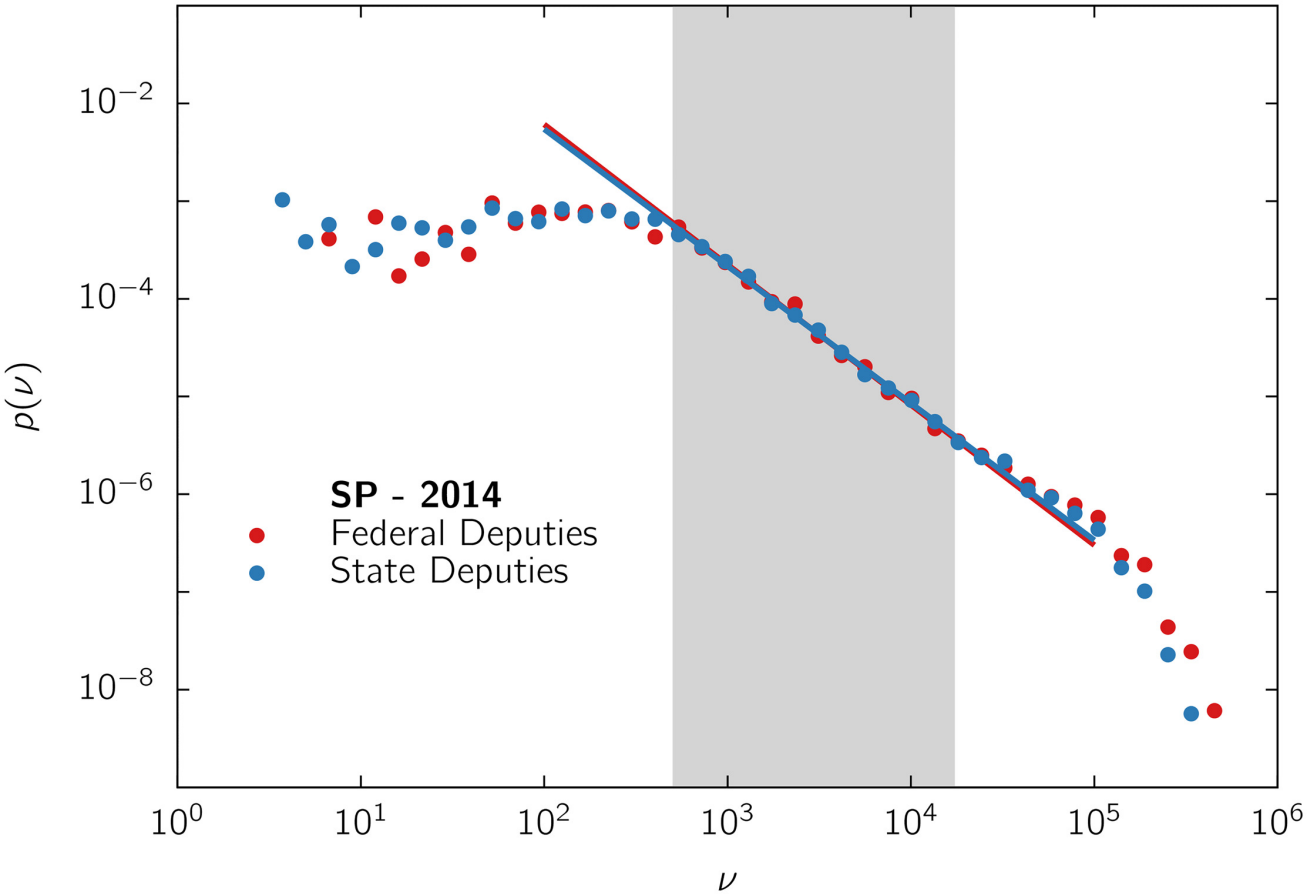
Probability density function (PDF) of the quantity of votes *p*(*v*). received by candidates for deputies of São Paulo state in 2014. Data are logarithmically binned [25]. PDFs are normalized (with unitary area). The solid lines represent a power-law fit, as explained in the text, performed over the data interval in the shadowed region.

The PDFs of the quantity of valid votes received by candidates, *p*(*v*), were estimated by constructing normalized histograms with logarithmic bins [25]. The typical shape of the PDFs in doubly logarithmic plot is illustrated in Fig 1, where we used the data of the elections for deputies of São Paulo state in 2014. The distributions for all the calendars of the several states, including the four most populated ones (São Paulo, Minas Gerais, Rio de Janeiro and Bahia), are shown in the Supporting Information (SI).

For example, for the data in Fig 1, we first tested the hypothesis of a log-normal distribution, observed in other cases [18]. However, it can be ruled out by the Kolmogorov-Smirnov test (at 5% significance level), while this test does not discard the hypothesis of a power law, after excluding the data corresponding to the initial plateau. In fact, small numbers of votes (up to less than ∼ 10^3^ from a total of ∼ 2 × 10^7^, in the case of the figure) appear to be almost uniformly distributed, within statistical fluctuations, forming a plateau or flat profile. This regime is followed by a scaling region that can be described by a power-law with a cutoff [26–28]. This cutoff is constituted by the large numbers of votes of famous people and/or very popular experienced politicians. But, recall that in these elections there are thousands of candidates and dozens of winners of seats for each state. Hence, although there can be a few highly voted candidates, the cutoff, typically, contains a minority of the total number of votes, and those outliers represent only a small fraction of the winners of seats. For instance, in the case of Fig 1, about 60% and 72% of the total number of votes (for federal and state deputies, respectively) correspond to the power-law region. It is also important to stress that most of the candidates gaining seats belong to that scale-free region. Therefore, it is a relevant portion of the distribution of votes that will be the focus of our attention. Although the exponent *α* may change from one case to another, there are qualitative regularities, common to the many data sets for different electoral years and states. Such regularities may reflect some general features and, in particular, the appearance of a power-law regime may be a signature of some nontrivial phenomenon. Differently, the cutoffs do not display a common pattern, indicating that they may have a different origin in each particular case, inherent to some individual candidates.

In order to obtain a quantitative characterization, we computed the exponent *α* by means of a least squares linear regression applied to the log-transformed PDF, over the shadowed interval depicted in Fig 1. (The use of the more sophisticated method in Ref. [29] is not adequate in our case, where *α* ≤ 1 occurs, see also SI.) Although in many cases the scale-free region can exceed the shadowed area, we opted to fix a shorter fitting interval, but common to all analyzed sets, to avoid introducing further fitting parameters and to guarantee the results reproducibility.

**Fig 2:**
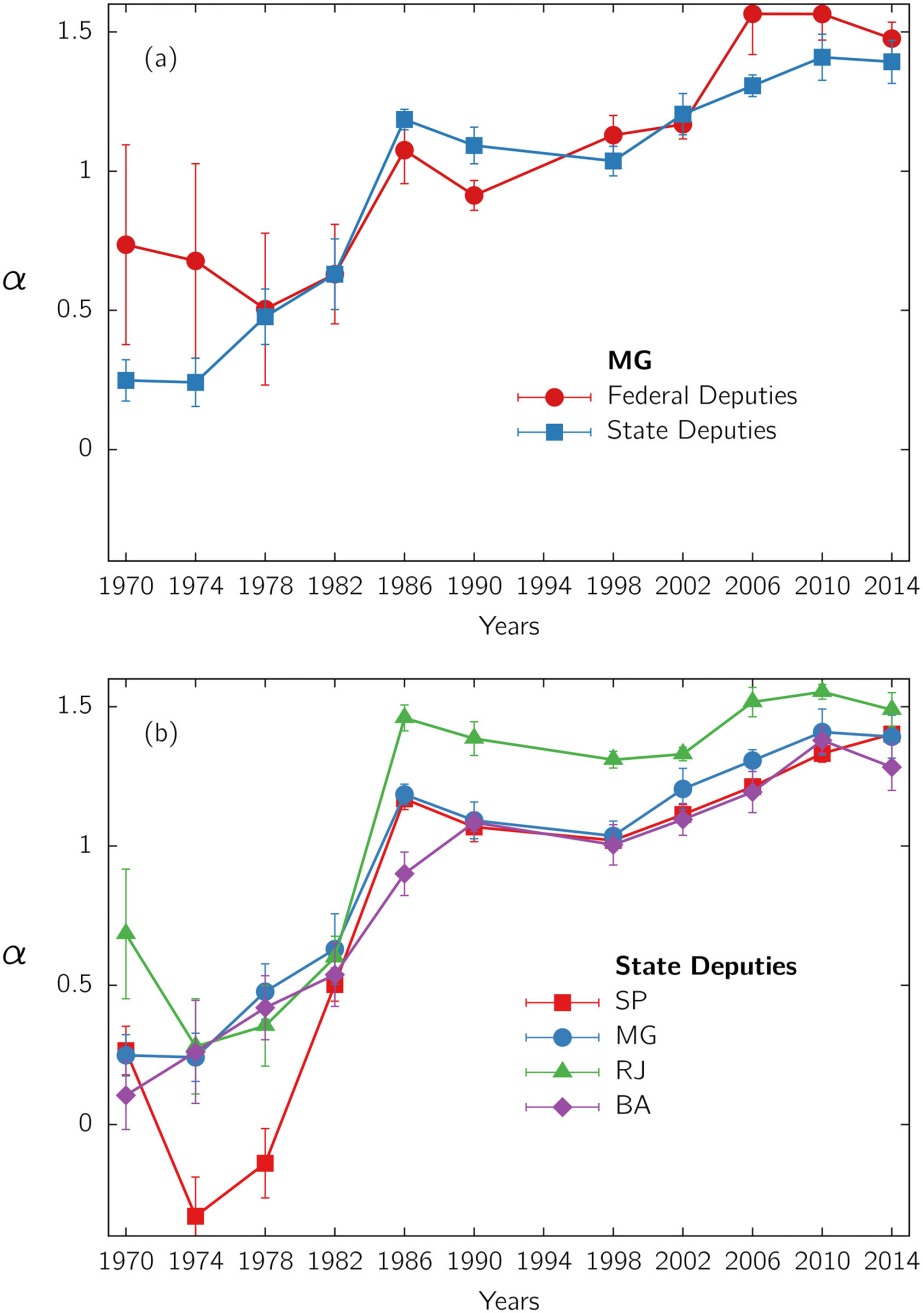
Exponent *α* of the PDF of votes *p*(*v*). as a function of the electoral year, from elections for (a) state and federal deputies in Minas Gerais (MG) state, and (b) state deputies in the four most populated states: São Paulo (SP), Minas Gerais (MG), Rio de Janeiro (RJ) and Bahia (BA). In all cases, the exponent *α* and its error were obtained from a linear least squares regression applied to the log-transformed PDF, over the scale-free region indicated in Fig 1.

Fig 2a shows *α* as a function of the electoral year for the state and federal deputies of Minas Gerais state (see also the PDFs and regressions for all the calendars in S2 and S6 Figs). Notice that, specially in the democratic period, the values of *α*, for both elections at the same calendar, practically coincide within error bars. For the military period, discrepancies and error bars are larger, since the statistics gets worst. In Fig 2b, we show the evolution of *α* for state deputies elections in the four most populated states (the corresponding PDFs and regressions are presented in S1–S8 Figs). Atypically, Sã Paulo state presents (in 1974 and 1978) even negative values of *α* for state deputies (see SI). We observe that, in contrast with the results for São Paulo and Minas Gerais in 1998 [10, 14], the exponent *α* typically differs from 1. Notice that *α* presents a tendency to increase with time, except for the peak at 1986. The two political regimes clearly have a different impact in the behavior of *α*. A rapid increase with time occurs during the dictatorship and a slow one during democracy. The variability of the exponent *α* cannot be explained simply by differences in the quantity of votes *N*_*v*_ or of candidates *N*_*c*_, although all tend to increase with time. In fact, for instance, the major changes of *α* as a function of time (around the record value in 1986) occur in a period of relative stability of the electorate size. On the other hand, it is clear in the example of Fig 2a the proximity of the values of *α* for federal and state deputies in the democratic period, despite the number of candidates *N*_*c*_ differs in both elections (see Materials and methods section), and mainly despite their pools of candidates are distinct. Noticeably, not only the exponents but the whole profiles, for both kinds of deputies, voted by the same electorate, are very close to each other, as illustrated in Fig 1. The main difference between the profiles for state and federal deputies is in the level of the flat region, indicating a larger fraction of candidates receiving few votes (plausibly amateurs) in the elections for state deputies, which also present a larger number of candidates (see the Materials and methods section).

The present statistical analysis suggests that the value of *α*: i) is not universal, ii) varies with time, and iii) seems to be associated to some property of the electorate or its interactions, independently of the pool of candidates and beyond the electorate size. Therefore, the scale-free regime may reflect a fundamental feature of the complex collective processes involved.

**Fig 3:**
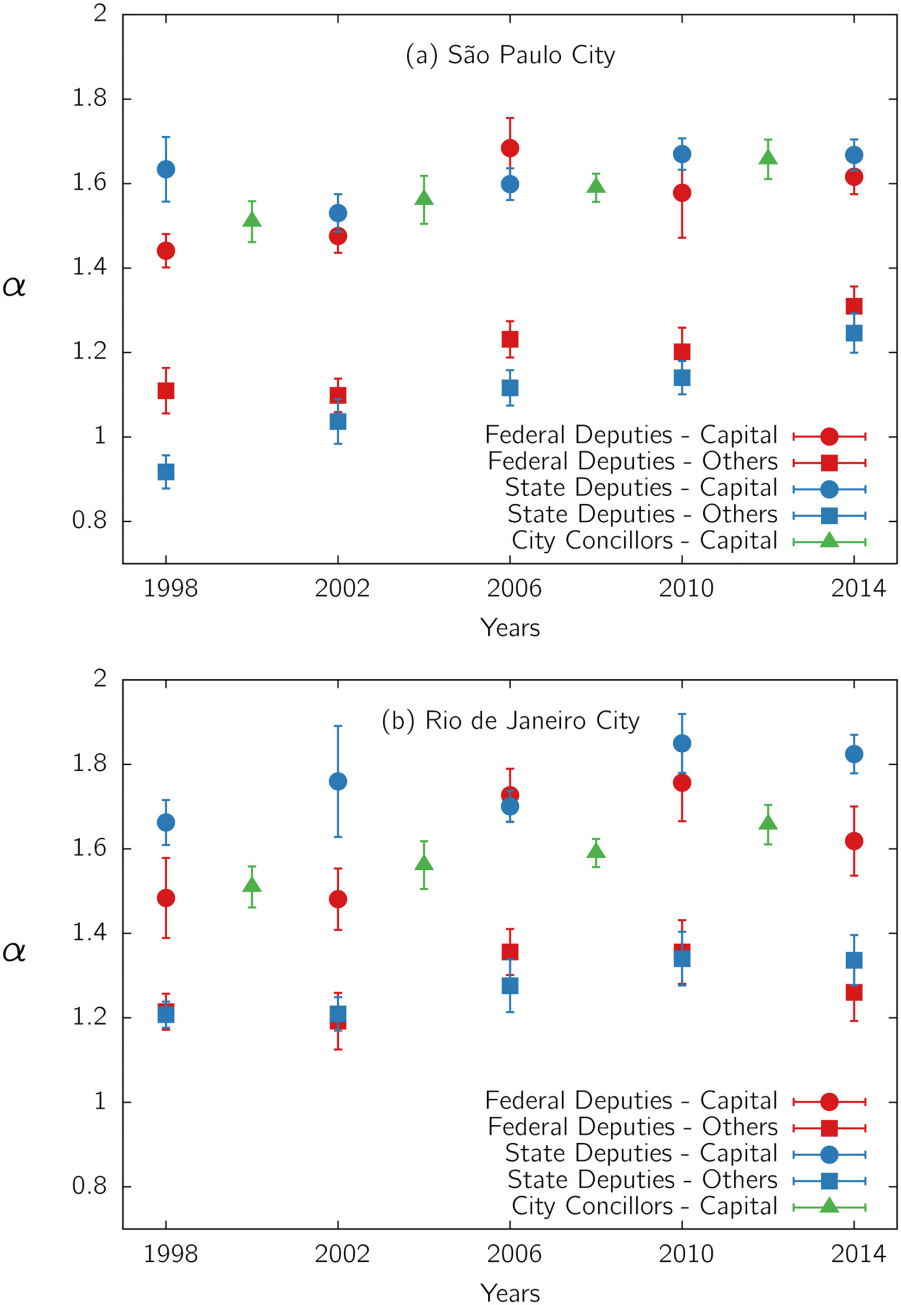
Exponent *α* of *p*(*v*). for candidates to city councilors and deputies (counting votes in a given city only), as a function of time, for (a) São Paulo and (b) Rio de Janeiro capital cities. The exponent *α* for deputies in the electorate of the state excluding the capital (denoted “others”) is also shown for comparison.

In order to see whether the same *α* is also observed in other cases involving the same electorate, we considered the elections for city councilors. In this case, the PDFs also present the same characteristic shape shown for legislators (see S9 and S10 Figs), but with a larger value of the exponent *α*, as already has been observed in previous literature [14]. For suitable comparisons, we restricted the analysis done for legislators to the electorate of each city considered (see S11 and S12 Figs). Because of the better statistics, we analyzed only state capital cities. The results for Rio de Janeiro and São Paulo cities are presented in Fig 3. Recall that elections for city councilors and deputies occur on alternate even years. Notice that although the candidates are different, the points representing the exponent *α* for deputies and city councilors exhibit a continuity, belonging almost to a same curve. This reinforces the hypothesis that this exponent is associated to a feature of the electorate and its interactions, independently of the pool of candidates. We also plotted, for comparison, the exponent for the remainder of the electorate in each state (see S13 and S14 Figs). It is also remarkable that the values of *α*, for large urban conglomerates, are larger than those for the population of the corresponding state out of the capital city, where interactivity is expected to be lower.

### Model

Previously proposed models reproduce the 1/*v* law but do not allow to describe the PDF decay of the form 1/*v* ^*α*^ with generic *α* that we observe in real data. We propose a simple model, where we describe the temporal evolution of the quantity of votes *v*_*i*_(*t*) received by each candidate *i* as a continuous variable. This is justified by the fact that the fractions *v*_*i*_/*N*_*v*_, where *N*_*v*_ is the total number of votes, tend to assume continuous values in the limit of a very large number of voters. In analogy to multi-species models of population dynamics, we consider that the quantity of votes *v*_*i*_, follows a logistic-like growth dynamics. Then, the time evolution is governed by a set of ordinary differential equations of the form

$$\begin{array}{c} \frac{dv_{i}}{dt}=r_{i}{\left(v_{i}+c\right)}^{\gamma }f_{i}(\{v_{j}\})\;, \end{array}$$ (1)

for 1 ≤ *i*, *j* ≤ *N*_*c*_, where *r*_*i*_, *c* and *γ* are positive parameters, and 0 ≤ *f*_*i*_ ≤ 1 limits the quantity of votes. This is a kind of mean-field model, independent of a particular explicit network of contacts.

Heterogeneity is a realistic ingredient of social dynamics, relative both to individual attitudes and to social influences [1]. Then, one should consider, in principle, that the equation parameters are drawn from a given probability distribution to reflect the heterogeneity of the pool of candidates, the electorate and their interactions. However, for simplicity, we will assume heterogeneity only in the parameters where it seems more crucial, as discussed below.

The coefficient *r*_*i*_ represents a kind of fitness of candidate *i*. It is related to the capacity of persuasion determined by a set of attributes attached to the candidate, such as its political proposal or personal appeal. It can be related to the personal attribute of a candidate known as *valence*[30, 31]. A negative value would lead to a vanishing fraction of votes. Then, for the candidates that win votes, the parameter *r*_*i*_ should take positive values. We consider that the candidates are heterogeneous with respect to this parameter, according to a given PDF of *r*, *f*(*r*). A similar kind of heterogeneity has been assumed in the cellular automaton of Ref. [12], where the probability of convincing is different for each candidate in a first stage where only candidates can influence voters.

The propagation of opinions about candidates can be thought as a sort of branching or multiplicative process [10, 14], in which a supporter of a given candidate can persuade, with a given probability, a fraction of its neighbors in the network of contacts, and each one of them, in turn, can influence others. In our model, the underlying network and multiplicative processes are not explicit but taken into account in an effective mean field manner, through the intrinsic *per capita* rate of growth of the number of votes *v*_*i*_.

In the standard case, the intrinsic rate of growth is constant, given by *r*_*i*_. However, if there were a positive feedback or “autocatalysis” between individuals, which is sound in human systems [32], the *per capita* rate would increase with *v*_*i*_, which can be described by *γ* > 1. A similar idea of self-reinforcing mechanisms, that can lead to herding behavior, have already been considered in the context of financial bubbles [33]. But avalanches of opinion propagation can occur in other contexts too [34, 35]. Notice that *γ* > 1 means that the probability of contagion becomes larger as the number of followers increases, like in autocatalytic reactions. A negative feedback may also occur and is represented by *γ* < 1. It is true that one could associate a different *γ* to each candidate, reflecting the particular feedback of the community in which the candidate exerts influence. However, in a first approach, we will consider that *γ* is predominantly homogeneous across a given electorate.

The introduction of parameter *c* allows to contemplate the existence of two realistic regimes, as considered in a previous model of elections [12]. In an initial phase when *v*_*i*_ ≪ *c*, candidates influence voters either directly or through their staff (by means of leafleting, outdoors, blogs, and other forms of propaganda), independently of the number of followers of each candidate. Otherwise, when the number of followers becomes large enough, people interact and become influenced by other electors too. These seem to be the two main mechanisms for dissemination of the preference for a given candidate, that is, propaganda and interaction within groups. In legislative elections, there are too many candidates to appear all in the media (TV, radio, newspapers, etc.), therefore, this effect is not so relevant as in the case of president-like elections with few candidates [36–43]. Parameter *c* can be related to the component of the spreading process which is independent of the number of followers *v*_*i*_. This parameter could be, in principle, a random parameter, different for each candidate, but, also in this case, we will take it as homogeneous.

Finally, we consider a logistic factor of the general form

$$\begin{array}{c} f_{i}\left(\left\{v_{i}\right\}\right)=1-\frac{v_{i}}{\beta_{i}N_{v}}-\frac{\sum_{j\neq i}\beta_{ij}v_{j}}{N_{v}}\;, \end{array}$$ (2)

which assumes that each quantity of votes *v*_*i*_ follows a logistic growth, up to a maximum value *β*_*i*_ *N*_*v*_, with 0 < *β*_*i*_ ≤ 1, corresponding to the portion of the electorate a candidate would conquer in the absence of other candidates. The last term is responsible for coupling the equations and describes the inter-specific competition amongst candidates in their struggle for conquering voters, where *β*_*ij*_ measures the competitive effect of candidate *j* on candidate *i*. When modeling voting processes, it is commonly considered, as more realistic, that the steady state has not necessarily been attained when the election occurs [12]. Then, as soon as the logistic term affects only the long-time dynamics, for simplicity, we consider that *β*_*ij*_ = *β*_*i*_ = 1, ∀*i*,*j*.

Besides randomness in the equation parameters, another source of heterogeneity may reside in the initial distribution of votes *v*_0*i*_ ≡ *v*_*i*_(0). It is clear that there are candidates with a political history and hence may start with a given community of followers, then, it would be interesting to investigate realistic initial distributions. However, if the initial numbers of votes are relatively small compared to *c*, as expected for the majority of the candidates, their precise values will not affect the distribution at later times. Then, for all candidate *i*, we will set *v*_0*i*_ = 1, corresponding to the minimal value of its own vote.

Under the above assumptions, the evolution equations are reduced to the simple form

$$\begin{array}{c} \frac{dv_{i}}{dt}=r_{i}{\left(v_{i}+c\right)}^{\gamma }(1-\sum_{j}v_{j}/N_{v})\;, \end{array}$$ (3)

for 1 ≤ *i* ≤ *N*_*c*_, where *γ* and *c* are positive constants and *r*_*i*_ is the only random parameter. Since we do not have any empirical knowledge *a priori* about *f*(*r*), we assume that *r* uniformly distributed in [0, 1]. (Notice that introducing a different maximal value of *r* will be equivalent to rescaling the time, then we chose the unit interval). Therefore, the model depends on only two parameters that appear to be the most relevant ones, namely *c* and *γ*.

**Fig 4:**
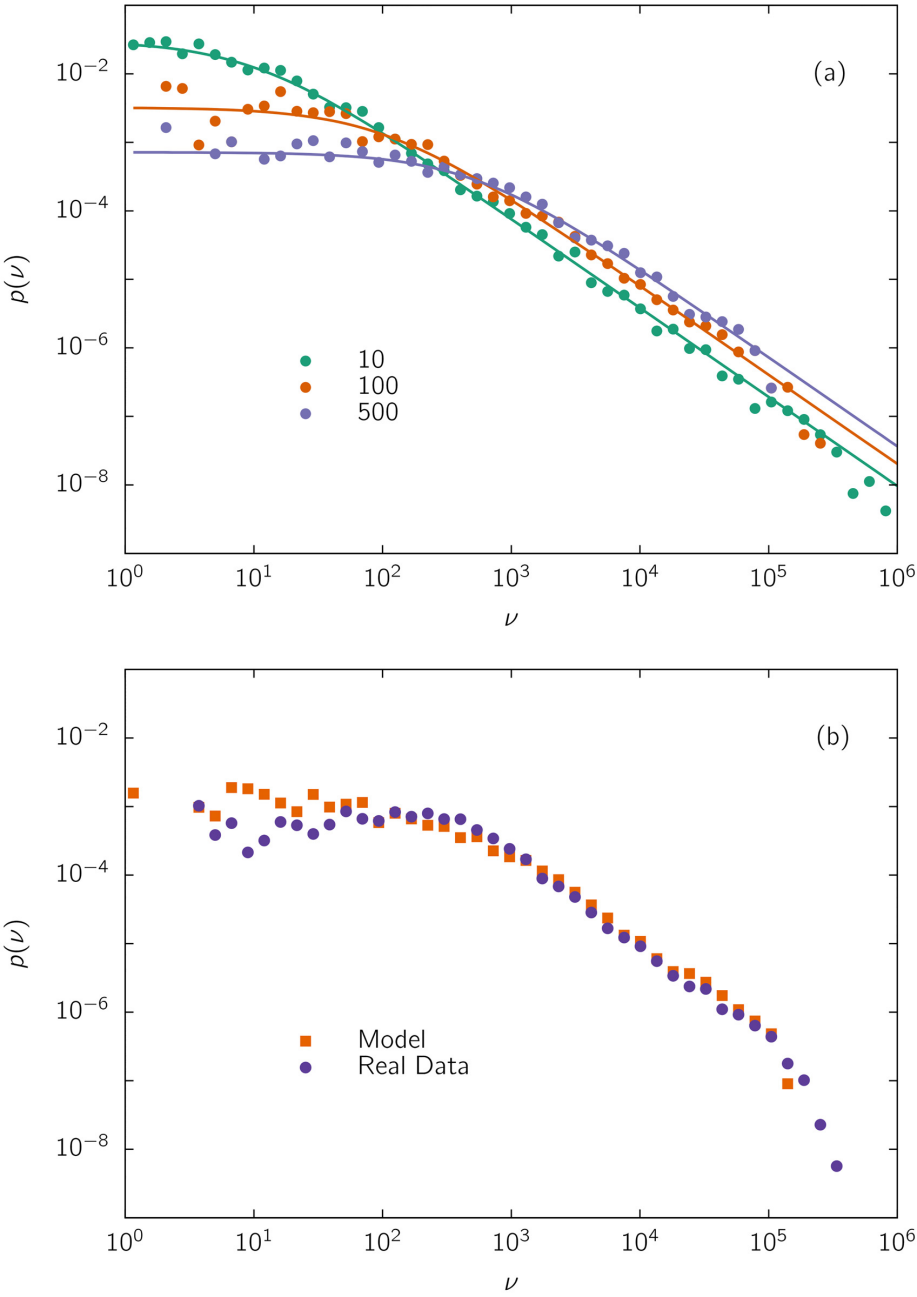
Performance of the model given by Eq (3). (a) Simulations using *γ* = 1.3 and *r*_*i*_ is uniformly distributed in the real interval [0, 1], for different values of *c* indicated on the figure, with *N*_*v*_ = 10^7^ and *N*_*c*_ = 10^3^. The initial condition was *v*_*i*_(0) = 1 for all *i*, and *p*(*v*) was computed from the values of *v*_*i*_ at the final steady state of a single realization. The lines correspond to the theoretical prediction given by Eq (5). (b) Comparison of the model with empirical data, for state deputies of São Paulo in 2014 (*N*_*v*_ = 17,348,029 and *N*_*c*_ = 1,876). Simulations were performed with real values of *N*_*v*_ and *N*_*c*_, with *γ* = *α* = 1.4 and *c* = 430.

From the numerical integration of this set of differential equations, we obtained *p*(*v*). We considered the values of *v*_*i*_ at the steady state, however, the results of the model are not significantly affected if we do not wait for the steady state to be reached. As illustrated in Fig 4a, the model produces outcomes qualitatively similar to the empirical ones, with a flat region and a power-law decay. In Fig 4b, we show a comparison of the model outcomes with results for state deputies in 2014. A very good accord of the PDF from simulations with that of real data is observed. The theoretical value of *α* can be identified with the intrinsic value *γ*.

In general, simulations fail in describing the initial portion, which is not completely flat. This could be improved by using a realistic initial distribution of votes, instead of *v*_*i*0_ = 1 for all *i*.

Notice also that the last phase of rapid decay of the distribution observed in the majority of empirical plots is not reproduced in the simulation. In fact, very popular candidates gaining very large numbers of votes are those contributing to the cutoff, but they are not contemplated by our model in its present form. (Although important features may be hidden in a cutoff [27, 28], we did not aim to model it, because no regular pattern is observed and only a few points typically constitute the cutoffs to allow a reliable comparison between modeling and real data.) A plausible way to include the cutoffs would be by modifying *f*(*r*), to take into account people with outstanding fitness, instead of a uniform distribution, and/or by introducing heterogeneity in other parameters and/or in the initial conditions. However, as far as we know, available empirical information does not allow to perform those extensions without introducing more free parameters.

**Fig 5:**
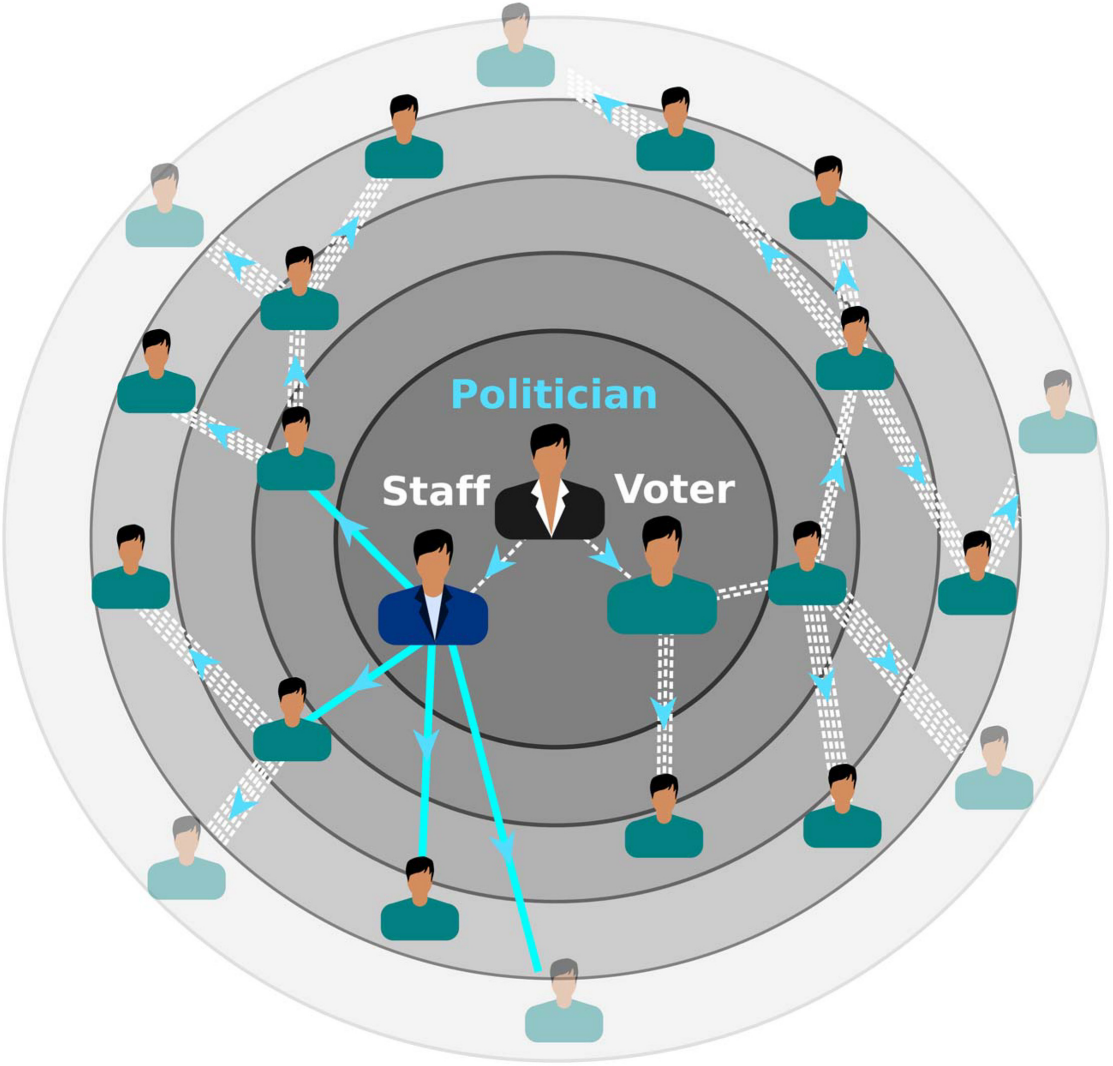
Pictorial representation of the model. A candidate gains followers either by means of the interaction of the candidate and her/his staff with the electorate or by means of a cooperative interaction of the electors. The arrows indicate contagion of ideas in time (evolving radially outwards). The figure illustrates the case of positive feedback: the probability of contagion increases (represented by thicker arrows) as the population of followers increases.

A pictorial representation of the model is given in Fig 5. It illustrates the two mechanisms of dissemination of ideas of a candidate *i*: i) without interactivity of the electorate, for low *v*_*i*_ compared to *c*, the dissemination to immediate contacts of the candidate dominates the diffusion process, and ii) when the number of followers *v*_*i*_ is large enough, the phase of interactivity of the electorate occurs with feedback (*γ* ≠ 1) or without it (*γ* = 1). The (positive) feedback is represented in the picture by thicker lines. In the measure than the number of followers of a candidate grows, the probability or rate *per capita* of convincing the nearest neighbors in the network of contacts also increases. The scale free regime is associated precisely to this interactive phase. Actually, there is a third stage, when the number of undecided people becomes small (∑*v*_*i*_(*t*) ∼ *N*_*v*_, hence the factor *f*_*i*_ tends to zero), a phase of competition between candidates operates, that is not represented in the picture.

The distribution *p*(*v*) resulting from the model can be analytically evaluated. At sufficiently short times the evolution of *N*_*i*_ will not feel the competition term and the equations are uncoupled. This corresponds to an initial phase in which voters interact mainly with candidates. As a first approximation, integrating Eq (3) with *f*_*i*_ = 1 for all *i*, we have

$$\begin{array}{c} v_{i}\left(t\right)≃v_{i0}{\left(1-\left(\gamma -1\right)r_{i}\;t\;v_{i0}^{\gamma -1}\right)}^{-1/(\gamma -1)}\;. \end{array}$$ (4)

This approximate expression is expected to hold up to a time close to the upper threshold given by the condition ∑*v*_*i*_(*t*) = *N*_*v*_ (*f*_*i*_ = 0). Eq (4) provides the dependence between the random variables *v* and *r* that allows to find the relation between their PDFs, by equating *p*(*v*)*dv* = *f*(*r*)*dr*. In particular, if *r* is uniformly distributed in the interval [0, 1], we obtain that the distribution of *v*(*t*) is

$$\begin{array}{c} p\left(v\right)=\frac{N}{{\left(c+v\right)}^{\gamma }}\;, \end{array}$$ (5)

for 1 ≤ *v* ≤ *v*_*max*_, where *v*_*max*_ is an upper bound, which guarantees the existence of a finite normalization factor N when *γ* ≤ 1. In fact, this approximate expression is in good accord with the results of the numerical integration of the model (see Fig 4). It also satisfactorily describes real distributions, with only two parameters *c* and *γ*, which control the crossover, between the plateau and the scale-free regimes, and the power-law decay, respectively. Then Eq (5) allows to identify the fitting exponent *α* with the intrinsic exponent *γ*, furnishing a possible interpretation for the origin of the scale-free regime. Namely, the empirical exponent *α* can be associated to the degree of feedback *γ* of the spreading processes.

It is worth noting that the candidates contributing to the power-law regime are not, in general, electorate-widely discussed, but they are discussed in large local communities (e.g., neighborhood, workplace, university, church, etc.) where feedback effects may abound. Moreover, the interaction of the electorate (word of mouth, phone calls, communication through emails, social networks, etc.) is the main way those candidates communicate their candidatures, differently to the popular candidates (contributing to the cutoff), that appear in the mass media.

Therefore, the free-scale regime seems to embody the relevant contribution of the electorate interactivity. This interpretation is reinforced by the fact that a given electorate, characterized by a degree of feedback or autocatalysis, presents similar values of *α* in different elections and for different pools of candidates.

## Discussion

We analyzed the distributions of votes *p*(*v*) for different Brazilian elections along the years of 1970–2014. During the democratic period, *p*(*v*) presents a rather flat region followed by a power-law decay. The power-law exponent changes over time, as shown in Fig 2. The flat region, associated to amateur candidates winning few votes, is absent during the military period. The extension of the flat region as well as the power-law exponent change from one case to another (see Suppl. Mat.). However, the responses of the same electorate (from a given capital city or state, at a given calendar) to distinct elections (with a different pool of candidates) present similar values of exponent *α*. This effect can be observed in Figs 1 and 3, and in the Suppl. Mat., suggesting that this exponent reflects a feature that is predominantly characteristic of the electorate.

In order to describe the stylized facts of vote distributions, we introduced a new model of opinion formation in elections, following a different approach than in previous literature. Our model consists in a *N*_*c*_-dimensional nonlinear dynamical system, similar to multi-species models of population dynamics. The heterogeneity of the pool of candidates is mirrored by the fitness parameter *r*, which is assumed to be uniformly distributed. Interestingly, the model allows to interpret the power-law regime in terms of the degree of feed-back or auto-catalysis of the electorate. The cutoffs are not contemplated by the model in its present form. However, it could still be improved to be more realistic in several ways, for instance, by considering empirical distributions of *r* and *c*, and/or of the initial distribution of votes *v*_0_. But, it would imply the introduction of more free parameters, as soon as that information is unavailable. Even in its present setting, the model already captures relevant ingredients, and seems plausible by its ability in describing the dominant features of the empirical distribution of votes.

The scaling region contains the majority of votes and most of the candidates gaining seats. Therefore, it is a relevant portion of the distribution of votes, both quantitatively and qualitatively, mainly because it seems to manifest electorate interactivity according to the modeling. This interactivity appears typically in the interval of votes from 500–1,000 to 10,000–50,000. The number of votes within this interval roughly reflects the size of social groups (in workplaces, neighborhoods, universities, churches, clubs, etc.) where an interactive regime can settle. Since their members have similar interests, auto-catalytic effects are expected to be more pronounced [32]. It is also worth remarking that, in contrast to the case of presidential elections, in legislative elections, even the most popular candidates are not usually discussed across the whole electorate of the state or city. Differently from the case of prominent figures, the ideas of the typical candidates are disseminated mainly by propaganda and mouth-to-mouth, as far as they do not appear in the media. For instance, in the case of city councilors, typical candidates have limited finances for propaganda and certainly do not appear in the media. So, it is reasonable that their main mechanism is mouth-to-mouth, plausibly driven by promises of the candidate to bring further resources to the community (e.g. schools, health centers, creches).

Differently, the few highly-voted candidates (forming the cutoff), well known politicians and public figures, are discussed across most of the electorate of the state or city, but they also appear in the media and have previous publicity that may allow them to win more votes than the interactive mechanisms. In fact, since the vote is mandatory, many citizens who are not interested in political discussions, may vote influenced by the media. Since the cutoff of *p*(*v*) may be determined by the very specific way prominent candidates capture their votes, it does not follow a general pattern. This makes difficult modeling the cutoff. Notice also that in some cases (shown in the SI, such as in small states or during the military government) the cutoff is lacking, probably associated to the absence of such prominent people. Also, a difficulty is that cutoffs are given by extreme events, as such, they possess low statistics for adequate modeling. For these reasons, their votes might depart from the power-law regime without a neat pattern. But, despite the cutoffs contain the highly-voted candidates, they typically represent a minority of the total number of votes.

According to our modeling, the nonuniversality of *α* appears to be a manifestation of the different degree of feedback through which voters can interact. This is in accord with the observation that the values of *α* coincide for the same electorate. Moreover, values of *α* are larger for urban centers, with higher interactivity than rural areas. It is also noticeable that *α* is typically larger than one for the democratic regime, possibly because of a positive feedback of the electorate.

**Fig 6:**
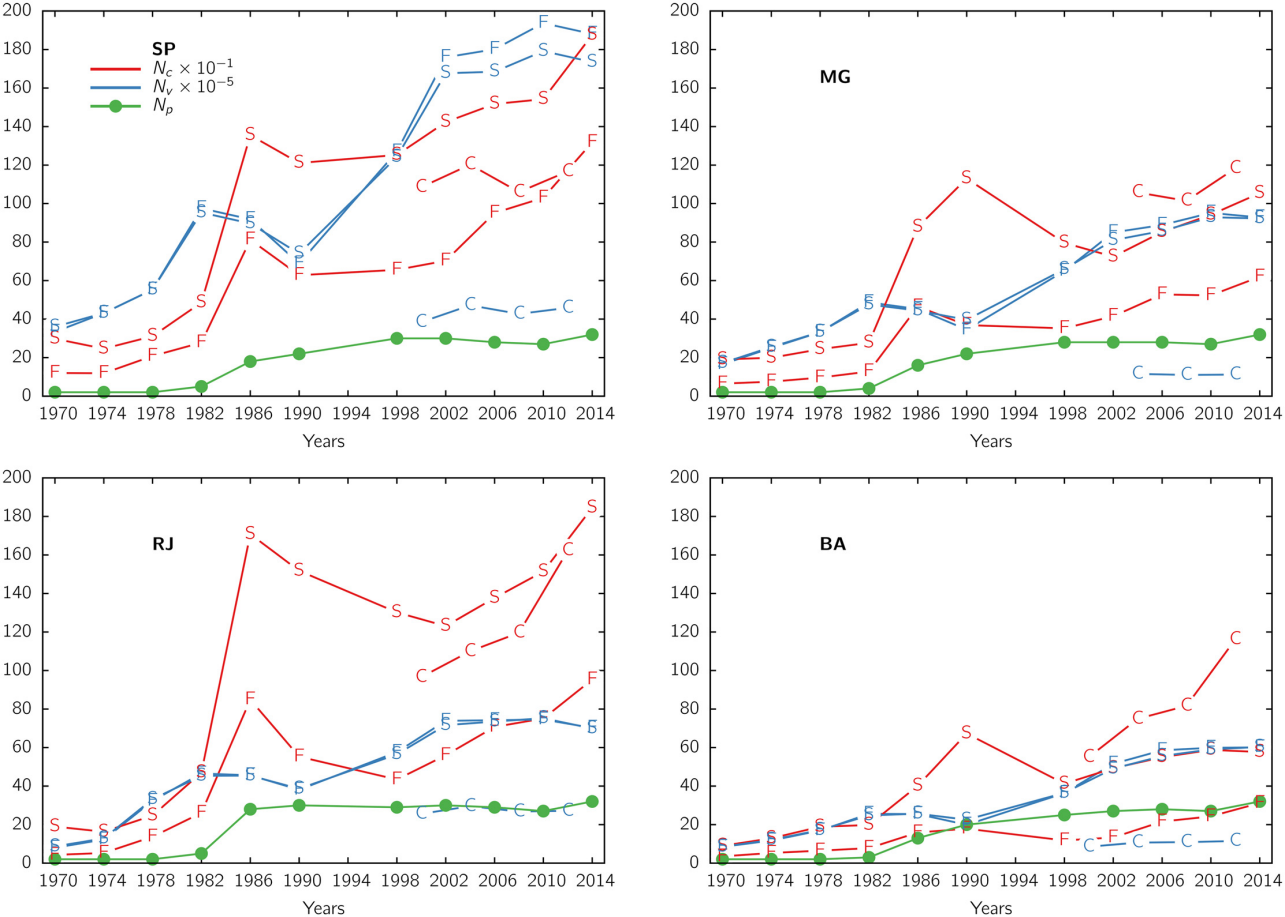
Electoral sizes. Quantities of valid nominal votes *N*_*v*_ (blue), number of candidates *N*_*c*_ (red) and number of parties *N*_*p*_ (gren), for state (S) and federal (F) deputies, and capital city councilors (C) as a function of the electoral year. Data for 1994 were unavailable.

Concerning the military regime, if one looks at an isolated single PDF (for instance in the panels from that period in S1–S4 Figs), it is difficult to recognize the pattern with flat and power-law regions observed for recent elections. In particular, the flat region is absent. However, if we look at the full temporal sequence in each one of those figures, one can recognize a definite slope in the shadowed area that tends to a small value when going back to the past. Lower than one, almost null, values of *α* are observed. According to the model, these small values can be associated to negative feedback (*γ* < 1) and/or absence of interaction of the electorate (large *c*), respectively. Both features are consistent with a dictatorial regime imposing severe restrictions to social interactivity, generating distrust and negative feedback. The null probability for low number of votes is also due to the restrictions eliminating the amateur candidates observed in later elections. However, the errors and uncertainty are large, due to the low number of candidates in the military regime (see Fig 6). In fact, the number of candidatures and electoral choices were initially small and increased during the transition from bipartidism to pluripartidism [24]. In the measure that some of the restrictions to democracy gradually relaxed towards the end of the military regime, the increase of *α* is observed, as well as the appearance of the plateau.

About the record value of *α* in 1986, let us comment that it may be related to diverse factors that favored the participation of a large and active electorate [24]. Besides marking the end of the military period, in this opportunity the legislators responsible for the elaboration of a new Constitution (the 1988 Constitution) were to be chosen [24]. It is remarkable that the singular historical context could in fact have promoted a particularly high positive feedback in the Brazilian electorate.

Following the model interpretation, the change of *α* in the last two decades, during the democratic phase, may be related to the fact that the way people interact is changing. In fact, the number of people accessing Internet has increased in that period and, mainly in the last decade, the number of people connected to social networks (such as, Orkut, Twitter, Facebook, etc.) has increased too. Furthermore, people may participate in more than one of these platforms and members may belong to groups of interest. These groups of people holding common interests are expected to be propitious for a positive feedback amongst their participants, however, the role of the internet and social networks in that respect is still unclear [44]. Apparent changes of *α* might also reflect changes in *f*(*r*), that is taken uniform in our model. These possibilities should be checked somehow through additional empirical data, to improve the modeling.

A limitation that can be attributed to this approach is that the role of parties was neglected. However, this was founded on the reasons exposed in the introduction. Mainly recall that only nominal votes were analyzed and that the vote is mandatory. The latter fact may explain the particularities of Brazilian vote distributions.

Despite several studies to characterize the structure and properties of social networks [45–48], the networks of ties and fluxes of influences among individuals in a population or electorate can be hardly known, in particular to allow a quantification of the feedback in the interactions. Moreover, how social contacts affect individuals and groups, induce collective effects, commitment to community and cooperation among individuals, are still open questions. Then, we believe that our present study might furnish, through the results of elections and the interpretation provided by the model, a metric to quantify the feedback in the process of contagion of electoral preferences. In that sense, we expect that our work will also motivate further research for alternative quantifiers. In the context of elections, this measure could be taken into account, for instance, to design campaign strategies according to the electorate mood. But it may be useful also in other contexts, since it reflects an important property of the population interactivity, its degree of feedback or herding behavior, which governs the propagation of opinions and influences.

## Materials and Methods

We analyzed the nominal votes of the elections for different kinds of legislators in Brazil: federal and state deputies, and also city councilors.

Federal deputies are representatives in the chamber of deputies of the national Congress, elected in a given state (from a total of 26 states in the federation and a federal district). Federal deputies are in proportion to the population of the respective state. State deputies are local representatives elected to serve in the unicameral legislature of each state. All deputies serve during a four-year term. All these elections occur simultaneously in the same countrywide electoral event, together with the election for president, governors and senators, at four-year intervals. Councilors serve during four-year terms in each city council and are voted in the same municipal election in which voters chose mayors, every four years, in even years alternating with presidential elections.

From a pool of candidates, an elector can choose one name of each class [24]. Besides the possibility of voting on a candidate (nominal vote), it is also possible to vote directly on a party (the so called *legenda* vote), without specifying a particular candidate. Actually, only a minority of the electorate practices the legenda vote (typically much smaller than 20%).

In Brazil the vote is mandatory. Let us also mention that abstention, invalid votes and blank votes are all ignored in vote counting for proportional seat allocation purposes.

In this work, we scrutinized the distribution of votes for candidates (nominal votes) only. That is, blank, invalid votes, as well as, valid legenda votes were not taken into account, following our aim of gaining insights on the formation of opinions about candidates.

Electoral sizes for the 4 most populated states are shown in Fig 6. The quantity of (valid nominal) votes, *N*_*v*_, and the number of candidates, *N*_*c*_, for both deputies and city councilors are plotted vs the electoral year. The electorate gradually increased, faster than the population, after the establishment in 1985 of the voluntary voting for illiterate and minors between sixteen and eighteen years of age.

Both kinds of deputies are voted in the same electoral event and only one name can be indicated by each voter, then the respective total number of voters (hence of votes) *N*_*v*_ are very close.

The total of candidates *N*_*c*_ for the state legislatures is always larger than for the national one, reflecting the larger number of total seats to be assigned. The number of candidates was lower during the military regime due to the several restrictions, increased after the restoration of pluripartidism in 1980 and attained a relative stabilization at a higher level after 1986. The number of parties *N*_*p*_ is also represented, evincing the period of bipartidism at the beginning of the dictatorship and the progressive reintroduction of the multiparty system.

Finally, we would like to comment that, although we are not aware of any suspicion of large irregularities in elections, especially during the democratic period and in particular after 1996 when the electoral process became electronic, the possibility of fraud [49] and mistakes in vote counting [50] cannot be ruled out. In any case, if irregularities are only occasional, they are not expected to distort significantly the statistics of the actual behavior of electors. This is specially true in our case, where we are analyzing elections with many candidates, and interested in the power-law regime.

## Supporting Information

S1 Text Vote distributions for federal deputies. (DOCX)

S1 Fig Vote distributions for federal deputies, in São Paulo (SP) state. These figures illustrate the general procedure adopted to analyze the distributions. The shadowed area corresponds to the interval where the scaling behavior typically occurs. Although in some instances the scaling region exceeds the shadowed area, we considered this common interval for regression analysis. In each case, a linear least square regression to the double logarithmic plot was performed. The line with the resulting slope *α* is also depicted. Notice that the flat region corresponding to small number of votes, prior to the (shadowed) scaling region, is absent in the years of military regime, Also, the slope in the shadowed region becomes smaller in that period.
(PDF)

S2 Fig Vote distributions for federal deputies, in Minas Gerais (MG) state. (PDF)

S3 Fig Vote distributions for federal deputies, in Rio de Janeiro (RJ) state. (PDF)

S4 Fig Vote distributions for federal deputies, in Bahia (BA) state. (PDF)

S2 Text Vote distributions for federal and state deputies. (DOCX)

S5 Fig Vote distributions for federal and state deputies, in SP state. (PDF)

S6 Fig Vote distributions for federal and state deputies, in MG state. (PDF)

S7 Fig Vote distributions for federal and state deputies, in RJ state. (PDF)

S8 Fig Vote distributions for federal and state deputies, in BA state. (PDF)

S3 Text Vote distributions for city councilors. (DOCX)

S9 Fig Vote distribution for city councilors in São Paulo capital. (PDF)

S10 Fig Vote distributions for city councilors in Rio de Janeiro capital. (PDF)

S4 Text Vote distribution for deputies in capital cities. (DOCX)

S11 Fig Vote distributions for federal and state deputies in São Paulo capital. (PDF)

S12 Fig Vote distributions for federal and state deputies in Rio de Janeiro capital. (PDF)

S13 Fig Vote distributions for federal and state deputies in São Paulo state, except the capital. (PDF)

S14 Fig Vote distributions for federal and state deputies in Rio de Janeiro state, except the capital. (PDF)

S5 Text Vote distributions for federal deputies in all other states. (DOCX)

S1 Table Number of votes and candidates for federal deputies in the election of 2014. (PDF)

S15 Fig Vote distributions for federal deputies in Acre (AC) state. (PDF)

S16 Fig Vote distributions for federal deputies in Alagoas (AL) state. (PDF)

S17 Fig Vote distributions for federal deputies in Amapá (AP) state. (PDF)

S18 Fig Vote distributions for federal deputies in Amazonas(AM) state. (PDF)

S19 Fig Vote distributions for federal deputies in Ceará (CE) state. (PDF)

S20 Fig Vote distributions for federal deputies in Espirito Santo (ES) state. (PDF)

S21 Fig Vote distributions for federal deputies in Goiás (GO) state. (PDF)

S22 Fig Vote distributions for federal deputies in Maranhõ (MA) state. (PDF)

S23 Fig Vote distributions for federal deputies in Mato Grosso (MT) state. (PDF)

S24 Fig Vote distributions for federal deputies in Mato Grosso do Sul (MS) state. (PDF)

S25 Fig Vote distributions for federal deputies in Pará (PA) state. (PDF)

S26 Fig Vote distributions for federal deputies in Paraíba (PB) state. (PDF)

S27 Fig Vote distributions for federal deputies in Paraná (PR) state. (PDF)

S28 Fig Vote distributions for federal deputies in Pernambuco (PE) state. (PDF)

S29 Fig Vote distributions for federal deputies in Rio Grande do Norte (RN) state. (PDF)

S30 Fig Vote distributions for federal deputies in Rio Grande do Sul (RS) state. (PDF)

S31 Fig Vote distributions for federal deputies in Rondônia (RO) state. (PDF)

S32 Fig Vote distributions for federal deputies in Roraima (RR) state. (PDF)

S33 Fig Vote distributions for federal deputies in Santa Catarina (SC) state. (PDF)

S34 Fig Vote distributions for federal deputies in Sergipe (SE) state. (PDF)

S35 Fig Vote distributions for federal deputies in Tocantins (TO) state. (PDF)

S1 Dataset Federal deputies. The columns give year, state, city, party, candidate name, and number of votes, obtained from Brazilian Federal Electoral Court data repository, available at http://www.tse.jus.br/eleicoes/estatisticas/repositorio-de-dados-eleitorais
(ZIP)

S2 Dataset State deputies. The columns give year, state, city, party, candidate name, and number of votes, obtained from Brazilian Federal Electoral Court data repository, available at http://www.tse.jus.br/eleicoes/estatisticas/repositorio-de-dados-eleitorais
(ZIP)

S3 Dataset City councilors. The columns give year, state, city, party, candidate name, and number of votes, obtained from Brazilian Federal Electoral Court data repository, available at http://www.tse.jus.br/eleicoes/estatisticas/repositorio-de-dados-eleitorais
(CSV)
