# Supplementary material for: Stylized Facts in Brazilian Vote Distributions
Source: PLoS One. 2015 Sep 29;10(9):e0137732. doi: 10.1371/journal.pone.0137732 (PMC4587976; doi:10.1371/journal.pone.0137732)
Supplement: S1 Text — (DOCX) [file pone.0137732.s001.docx]

**Vote distributions for federal deputies.**

In S1-S4 Figs., we show the distributions for federal deputies across all the available calendars, for the four most populated states (SP, MG, RJ and BA, respectively), hence, those with better statistics (the remaining states are shown in S15-S35 Figs.). These examples illustrate the general procedure adopted to analyze the distributions.
