# Supplementary figures and images for: Stylized Facts in Brazilian Vote Distributions
Source: PLoS One. 2015 Sep 29;10(9):e0137732. doi: 10.1371/journal.pone.0137732 (PMC4587976; doi:10.1371/journal.pone.0137732)

SP  
• Federal Deputies

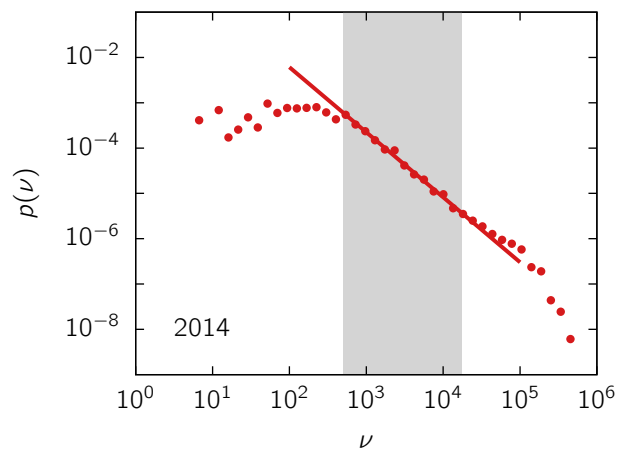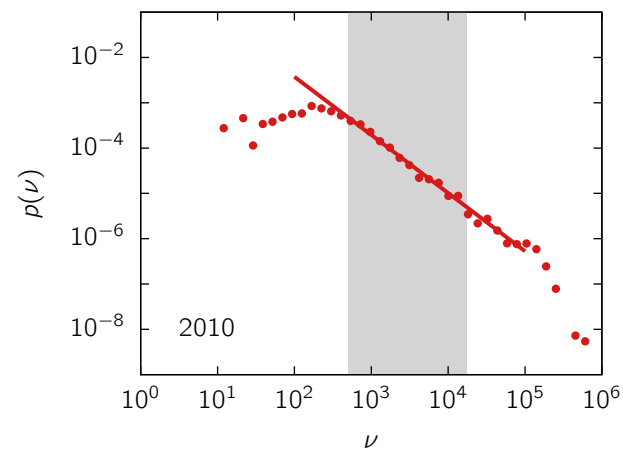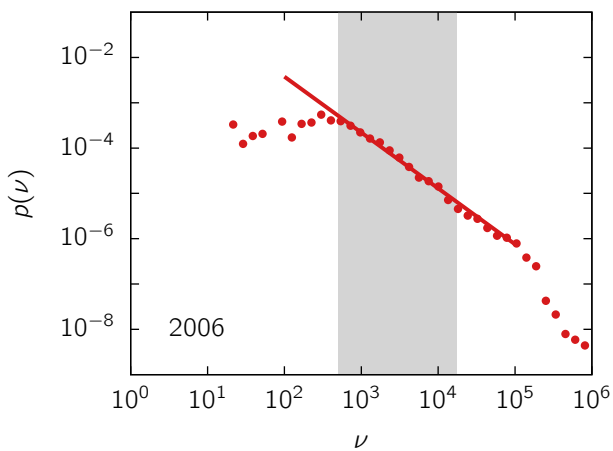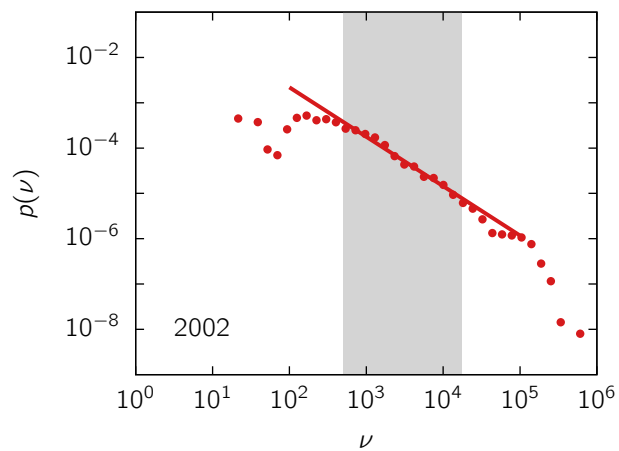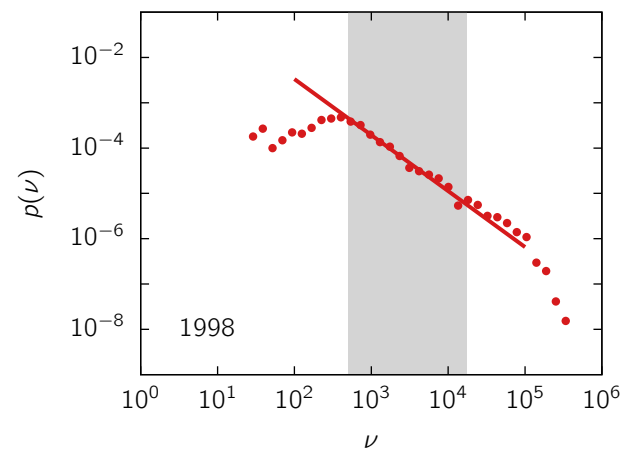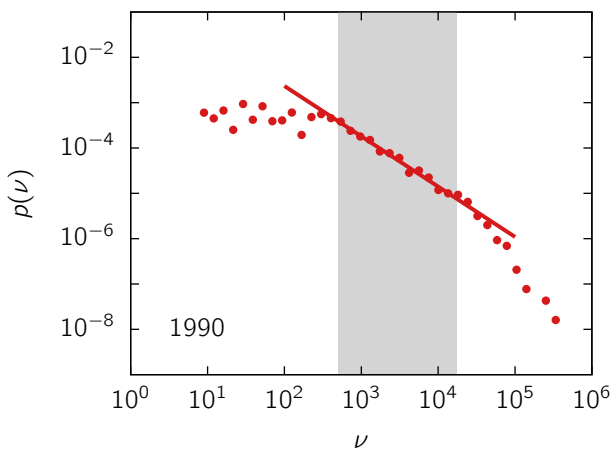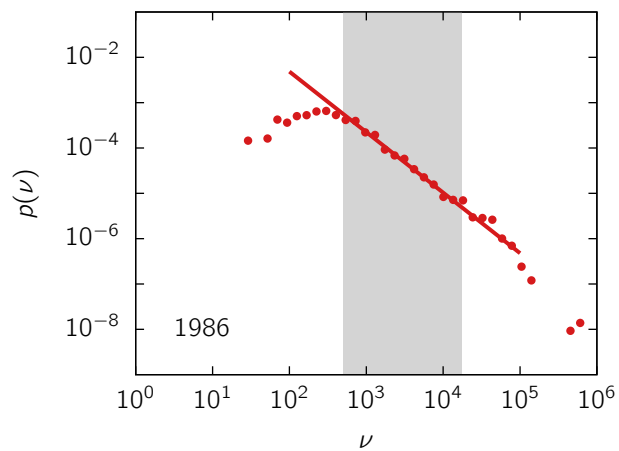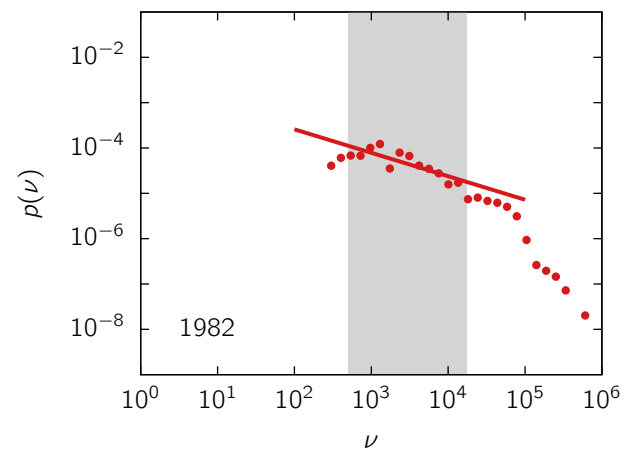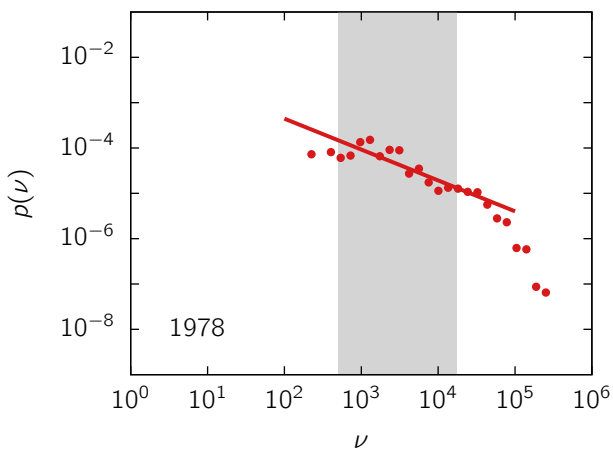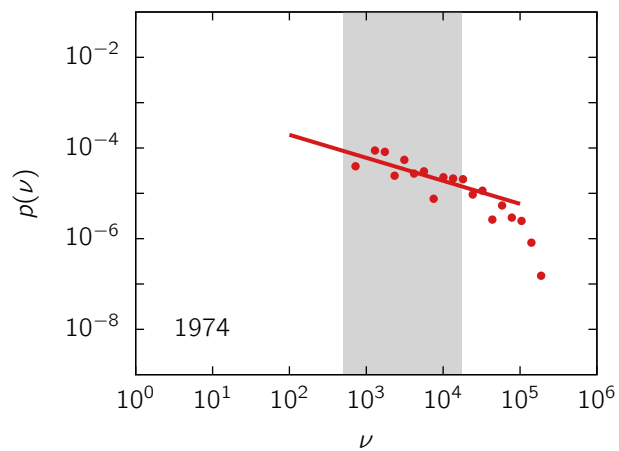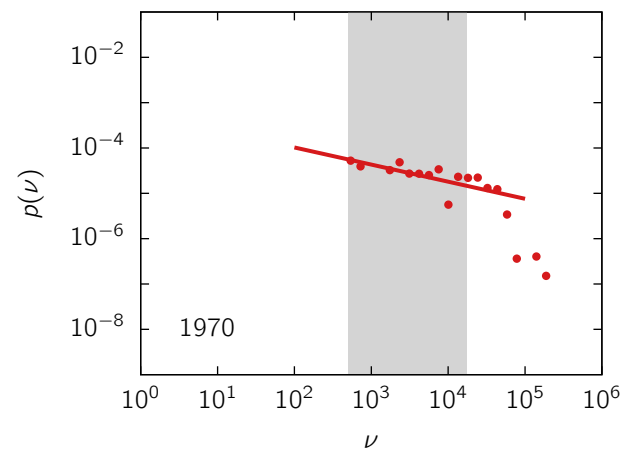

Supplement: S1 Fig — These figures illustrate the general procedure adopted to analyze the distributions. The shadowed area corresponds to the interval where the scaling behavior typically occurs. Although in some instances the scaling region exceeds the shadowed area, we considered this common interval for regression analysis. In each case, a linear least square regression to the double logarithmic plot was performed. The line with the resulting slope α is also depicted. Notice that the flat region corresponding to small number of votes, prior to the (shadowed) scaling region, is absent in the years of military regime, Also, the slope in the shadowed region becomes smaller in that period. (PDF) [file pone.0137732.s002.pdf]

MG  
• Federal Deputies

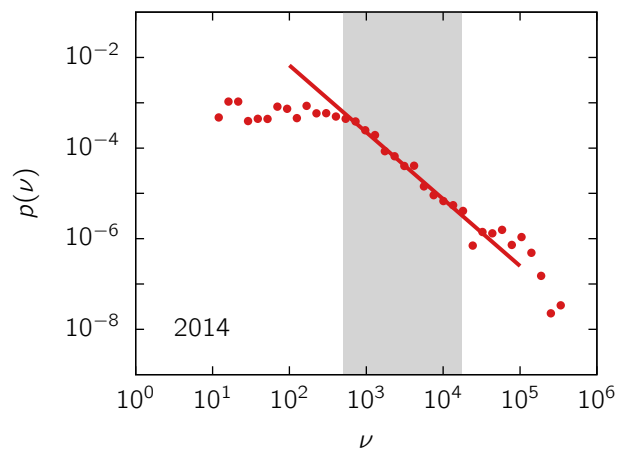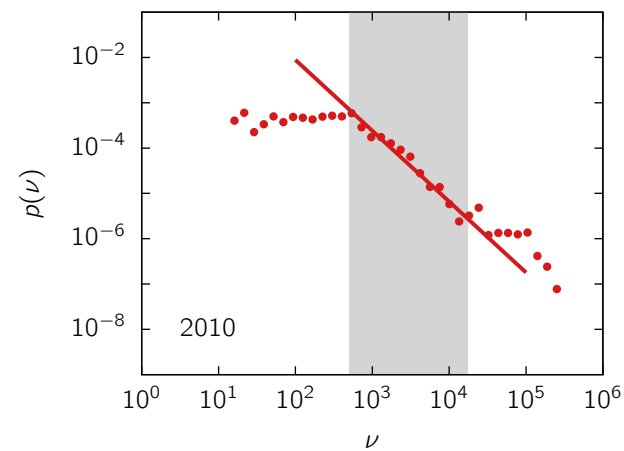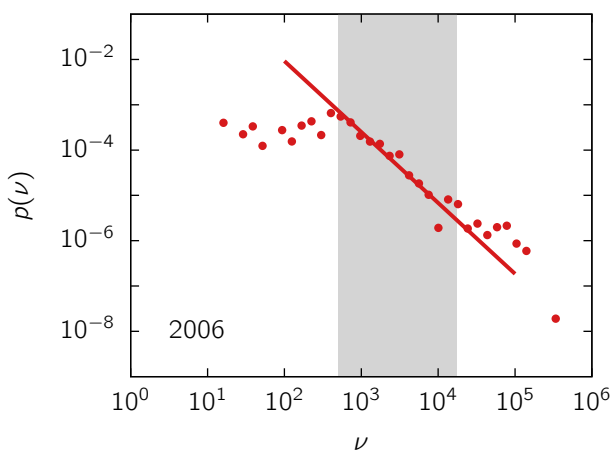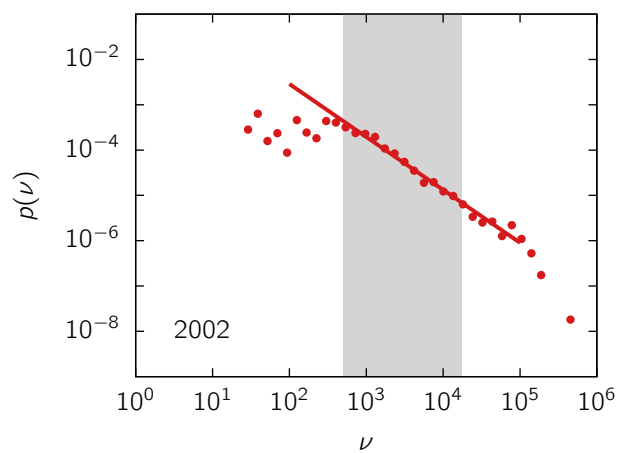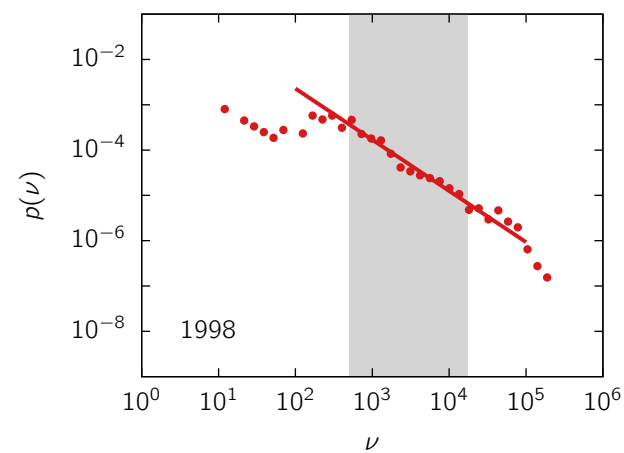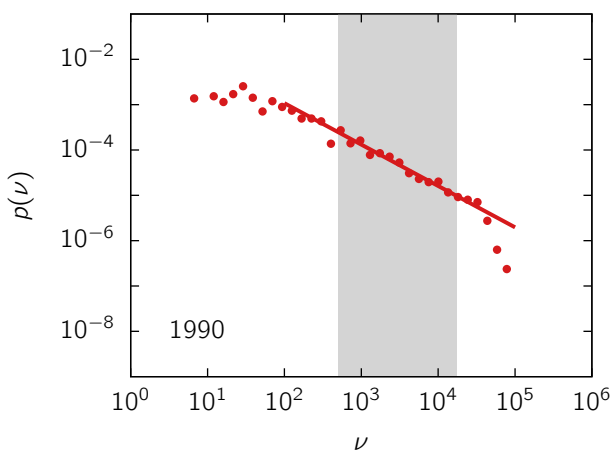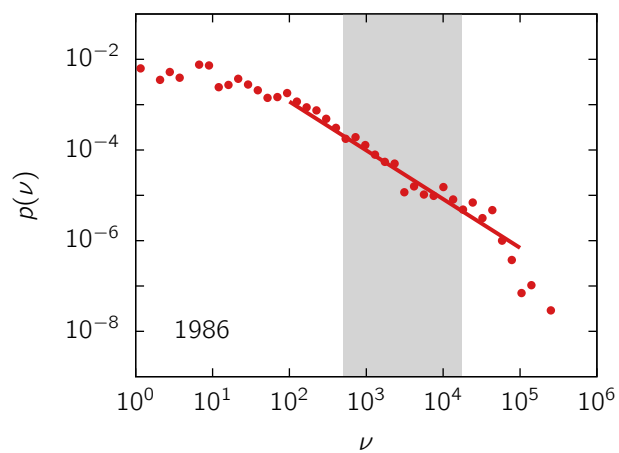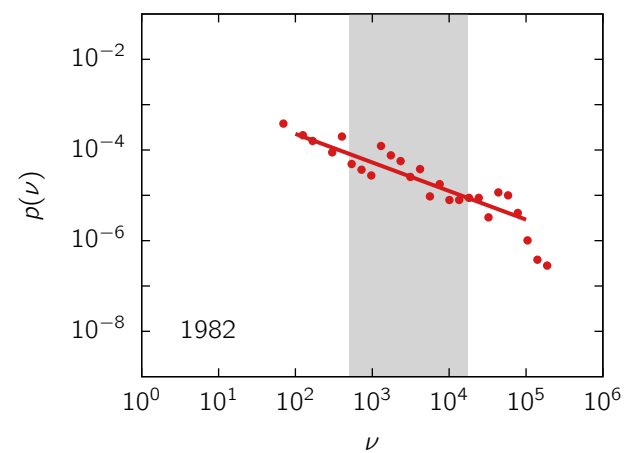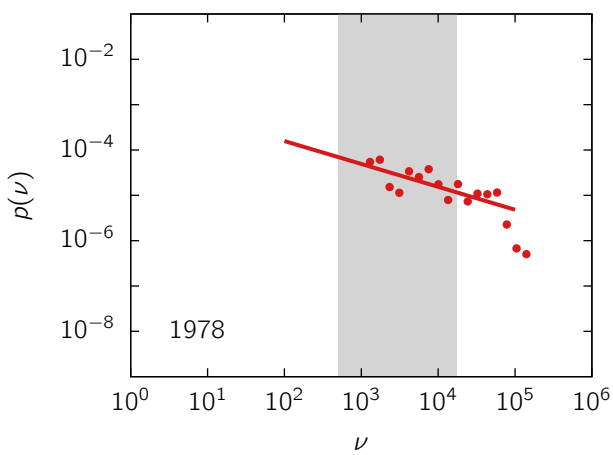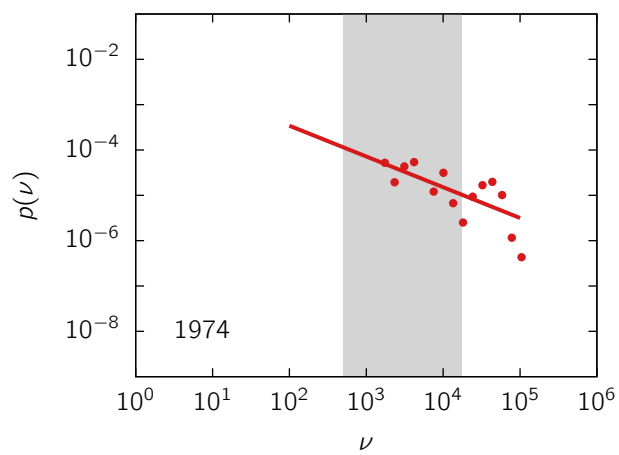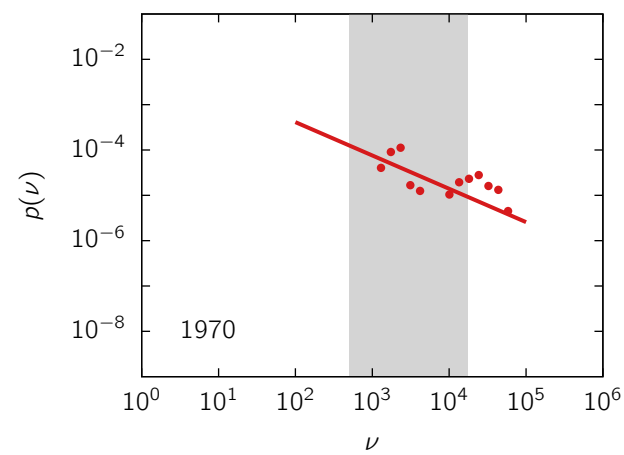

Supplement: S2 Fig — (PDF) [file pone.0137732.s003.pdf]

RJ  
• Federal Deputies

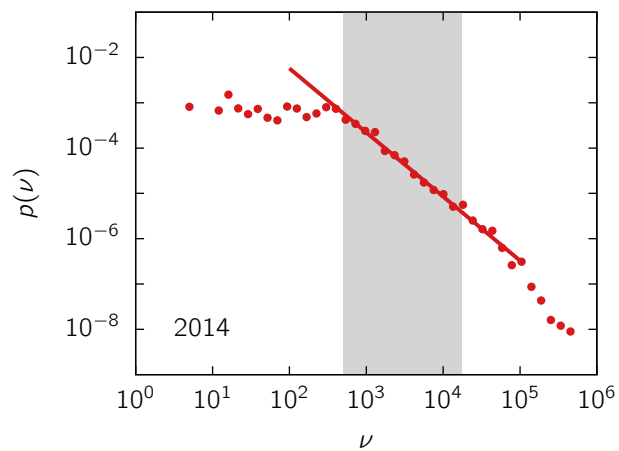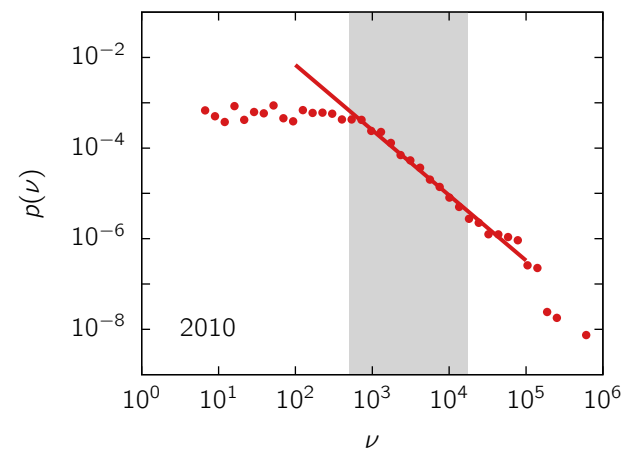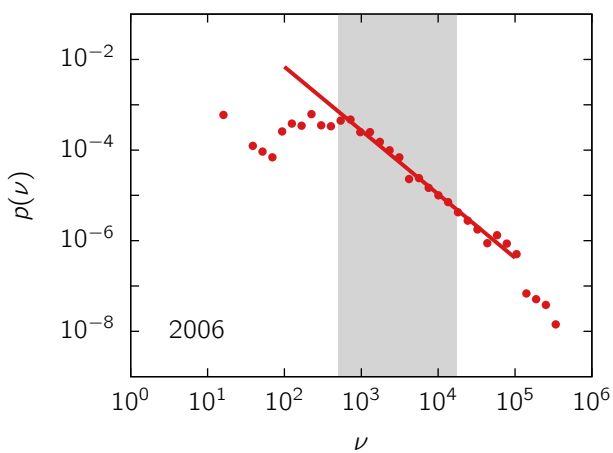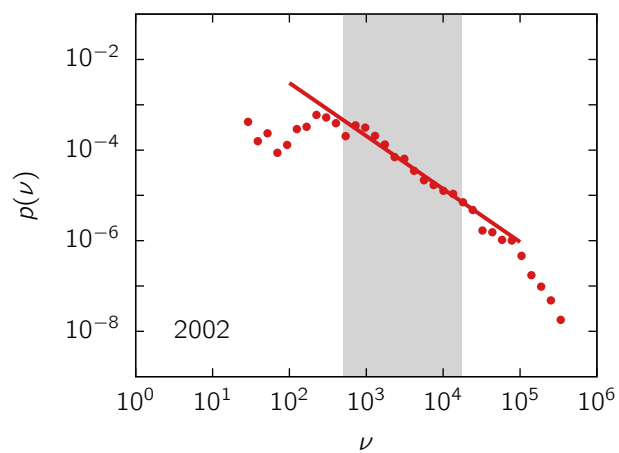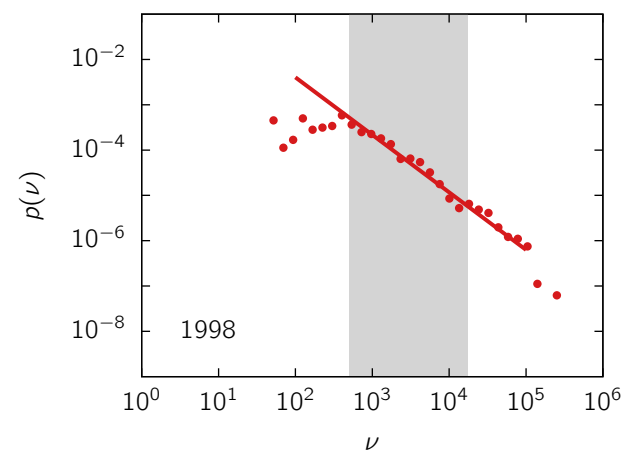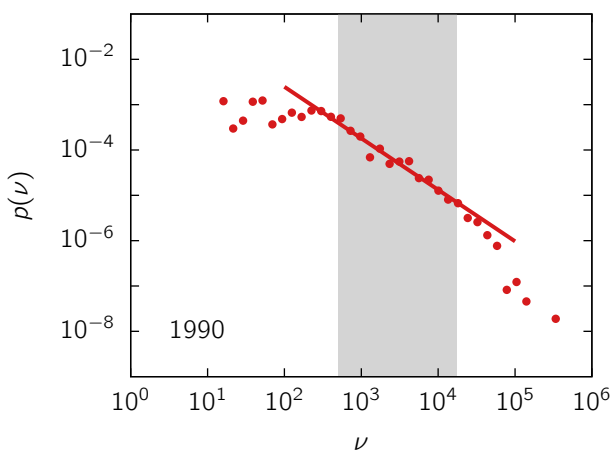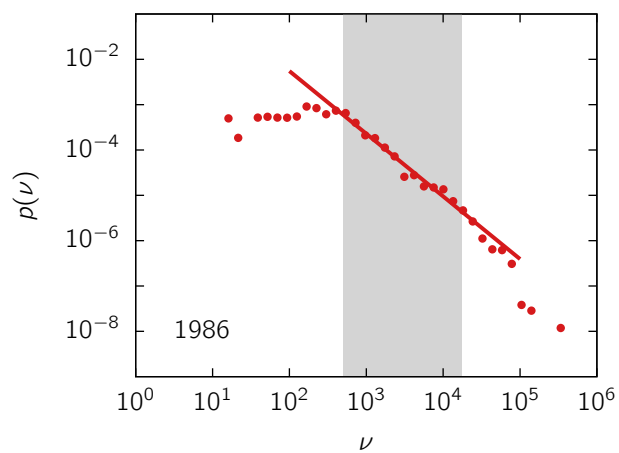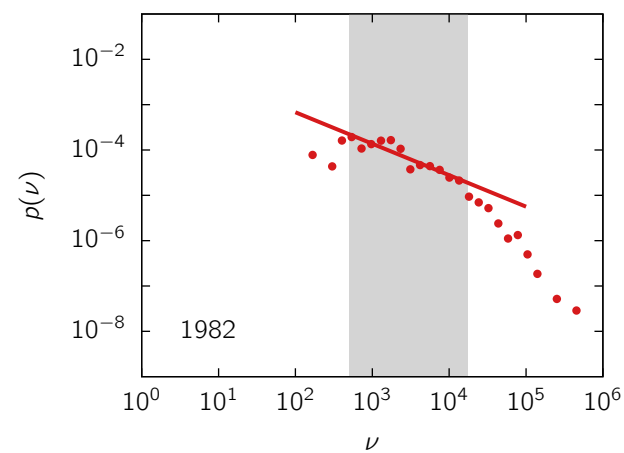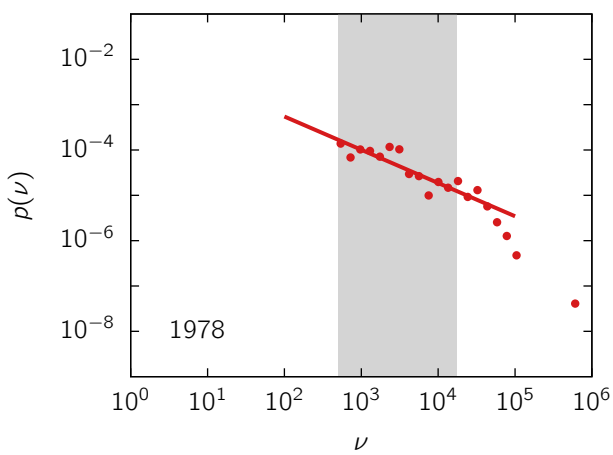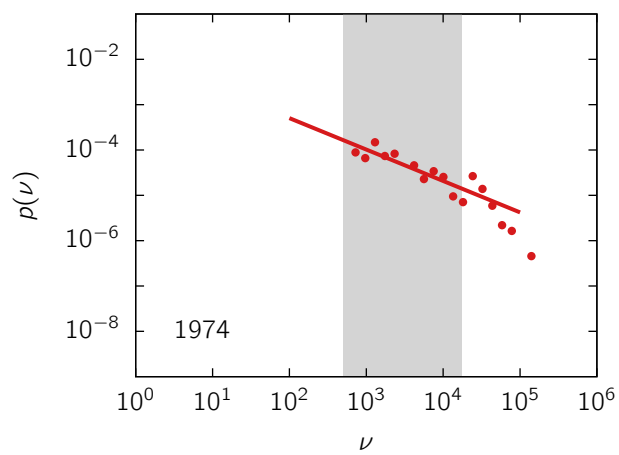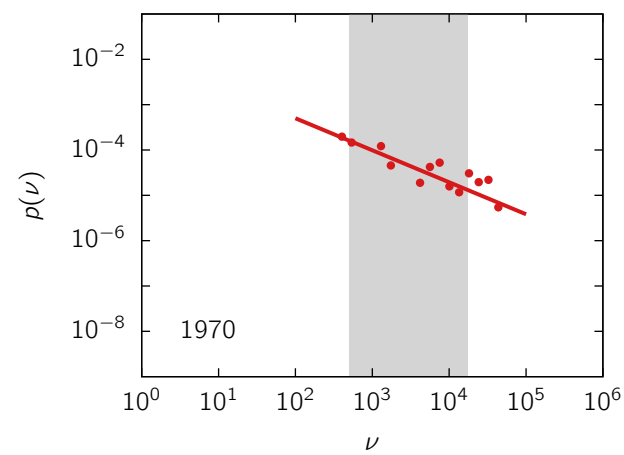

Supplement: S3 Fig — (PDF) [file pone.0137732.s004.pdf]

BA  
• Federal Deputies

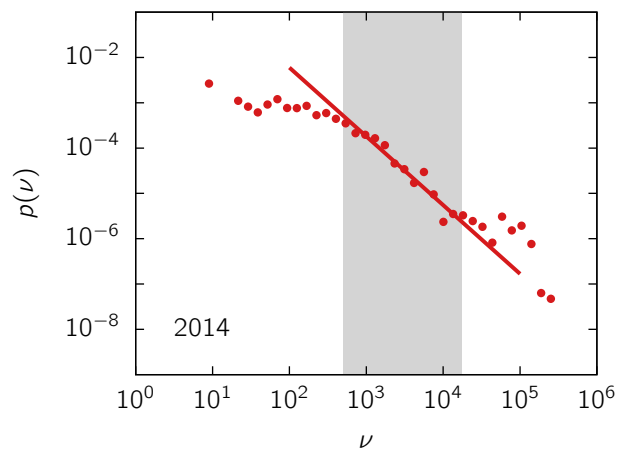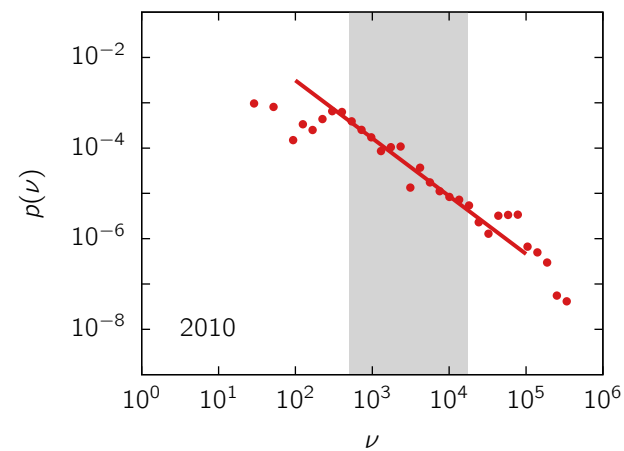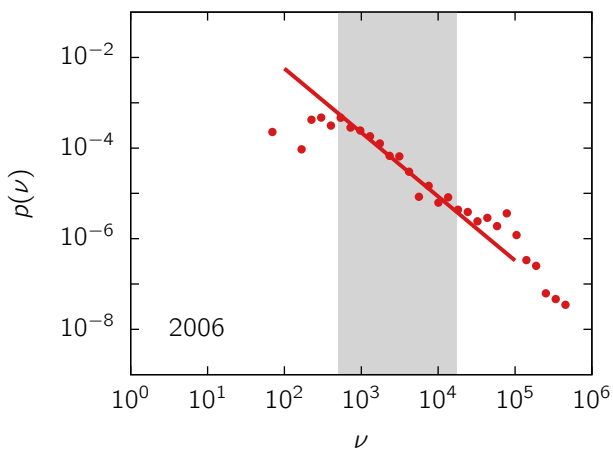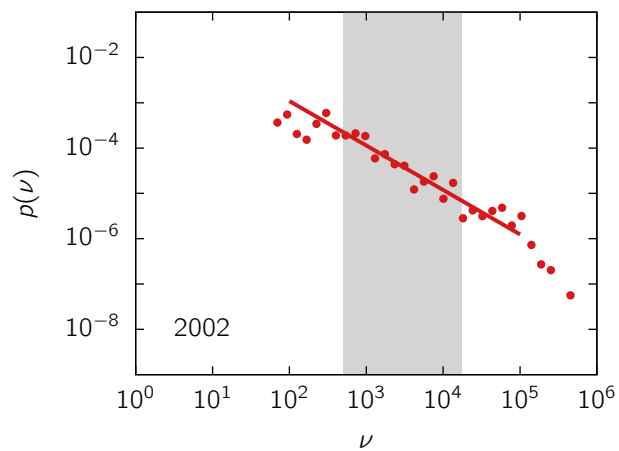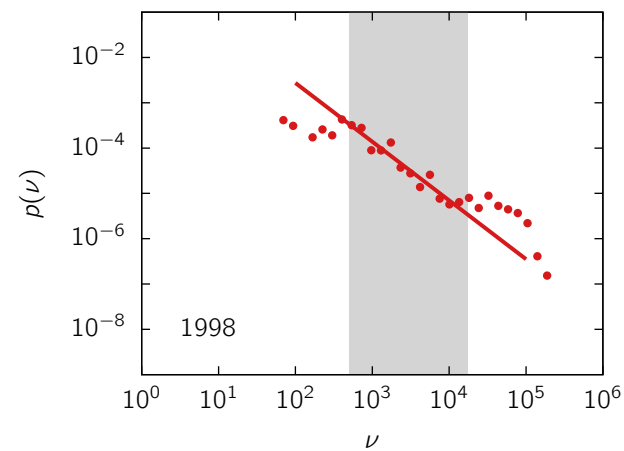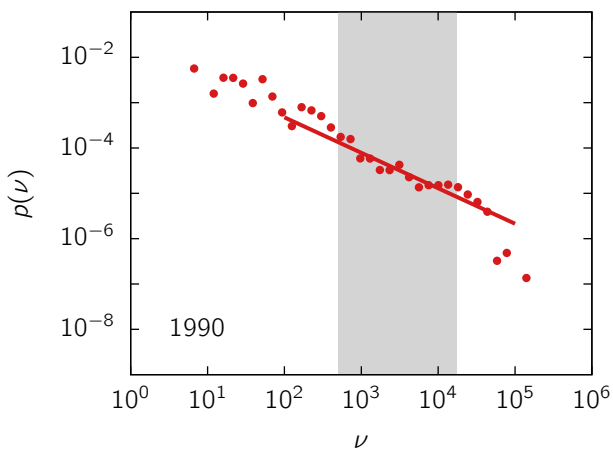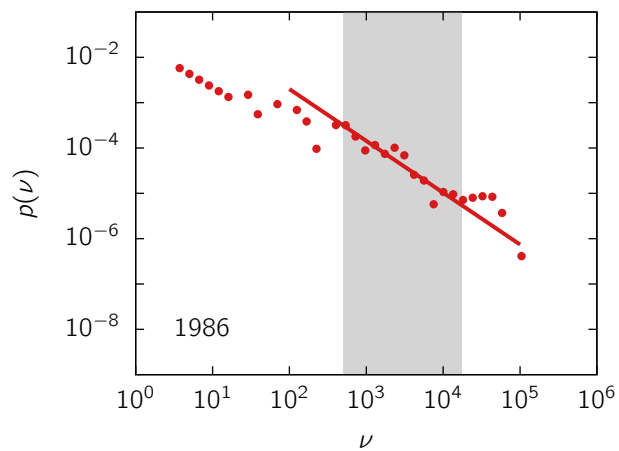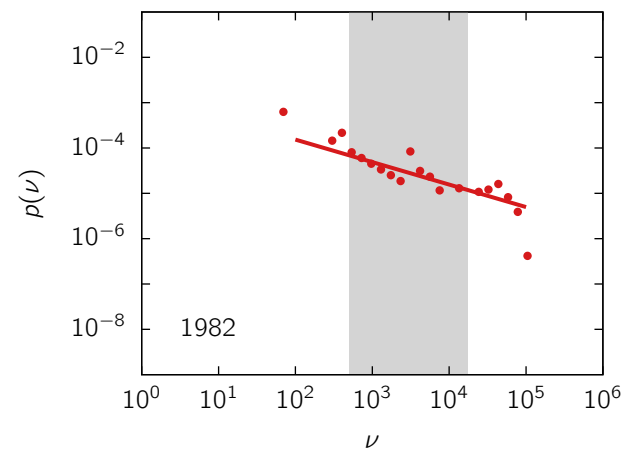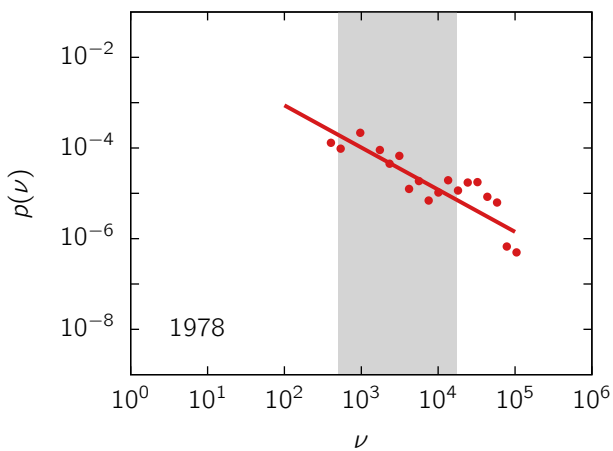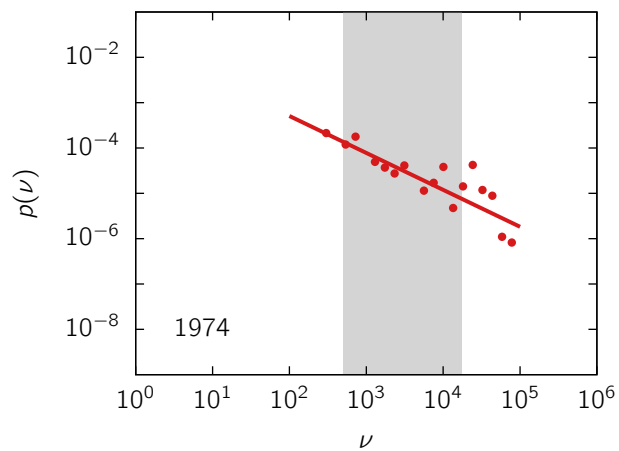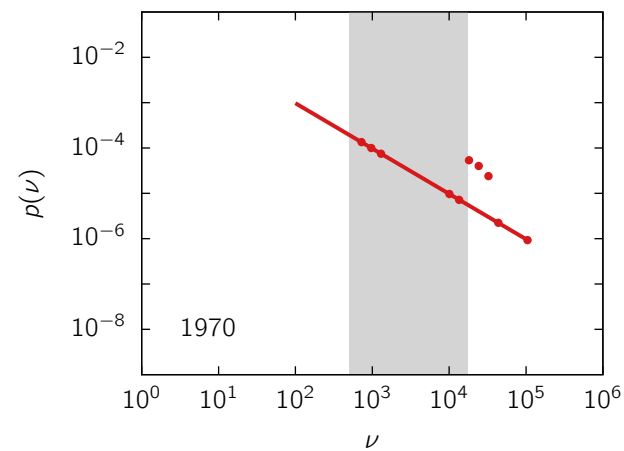

Supplement: S4 Fig — (PDF) [file pone.0137732.s005.pdf]

SP  
• Federal Deputies  
• State Deputies

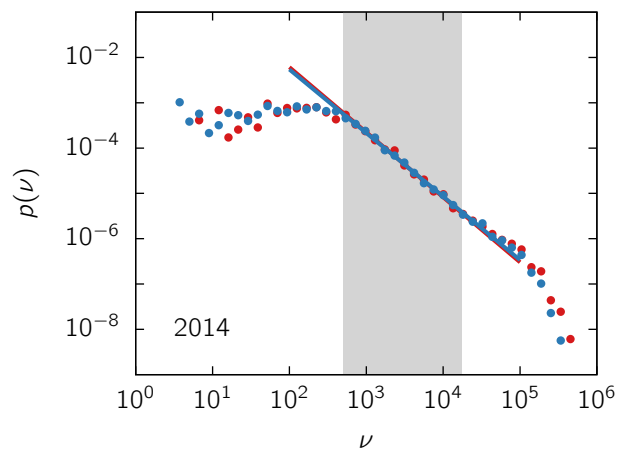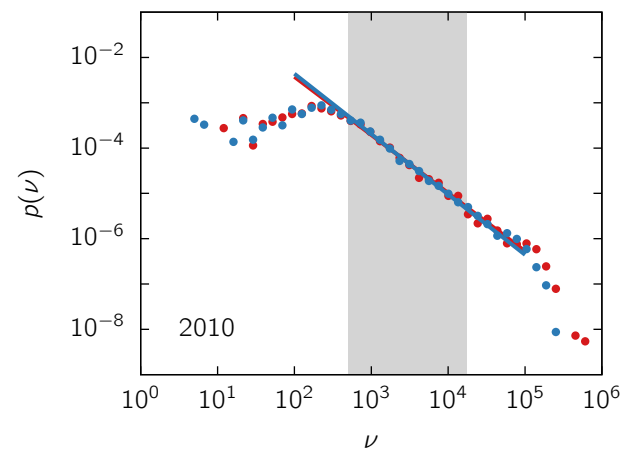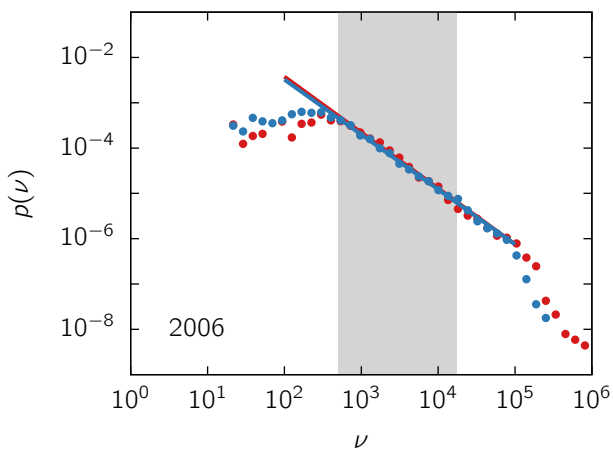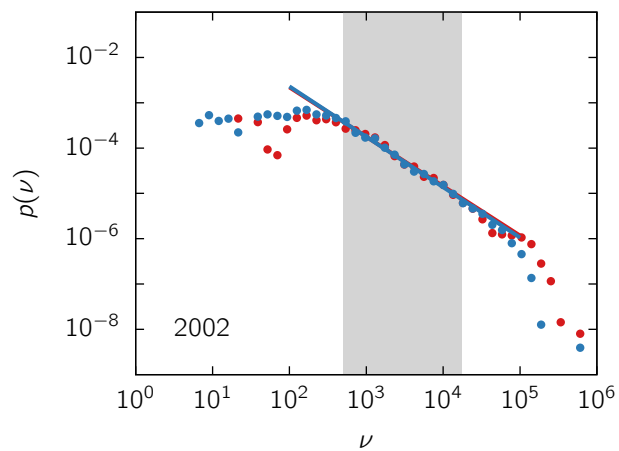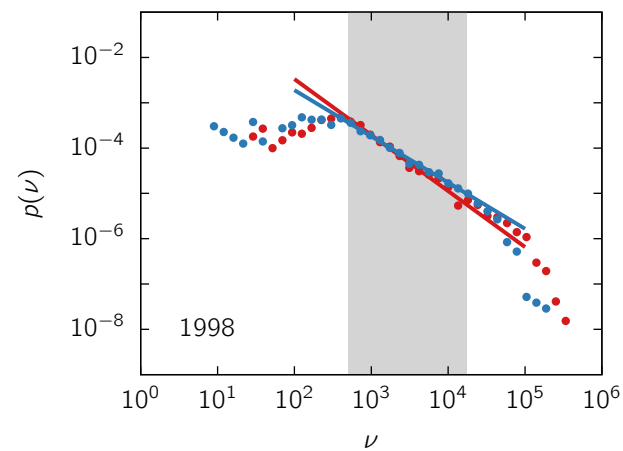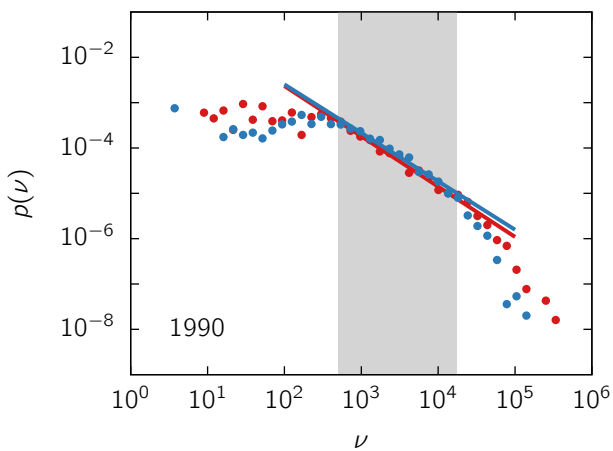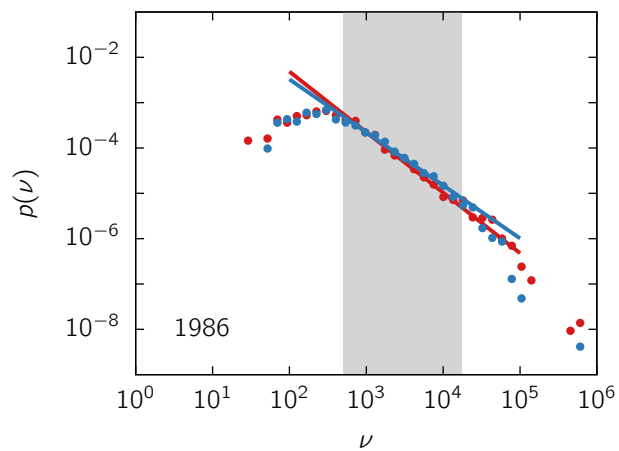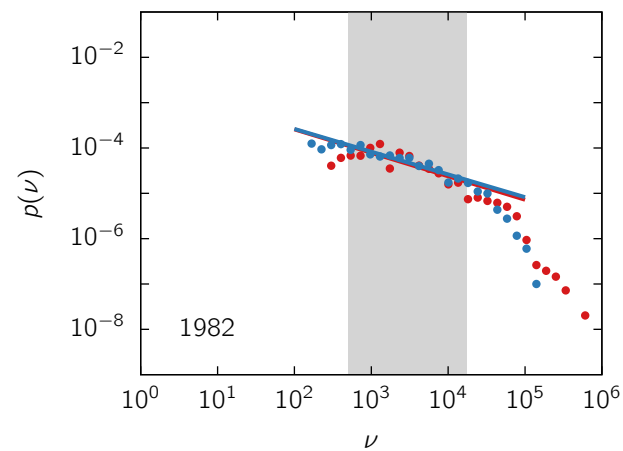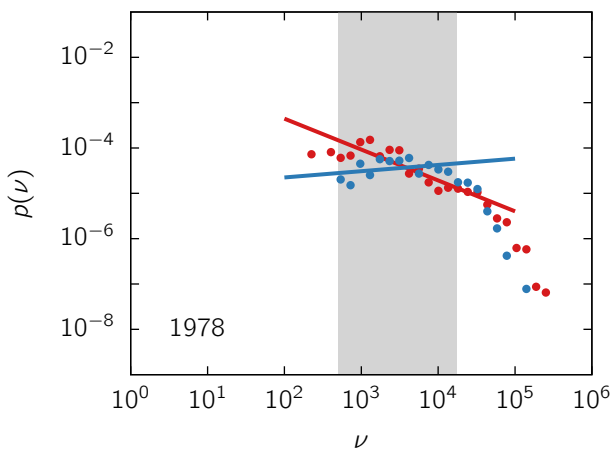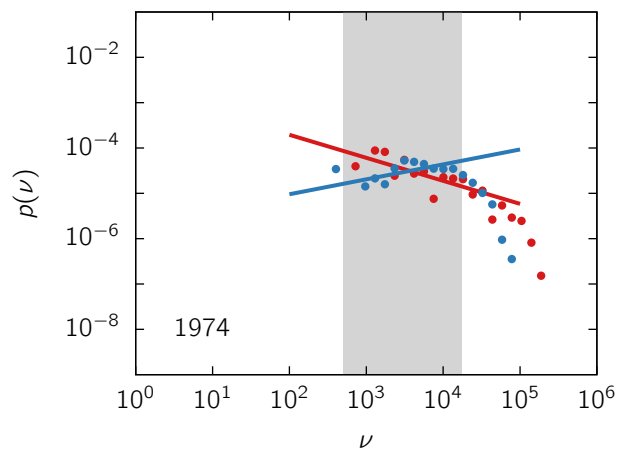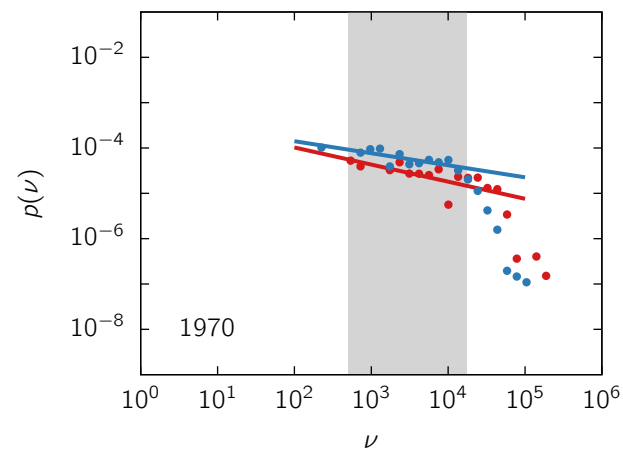

Supplement: S5 Fig — (PDF) [file pone.0137732.s007.pdf]

RJ  
• Federal Deputies  
• State Deputies

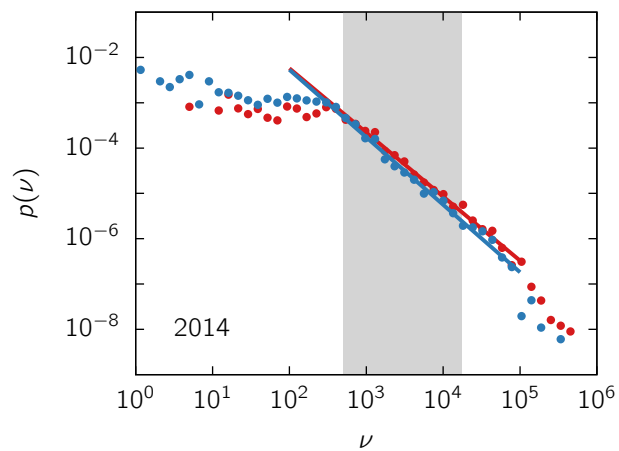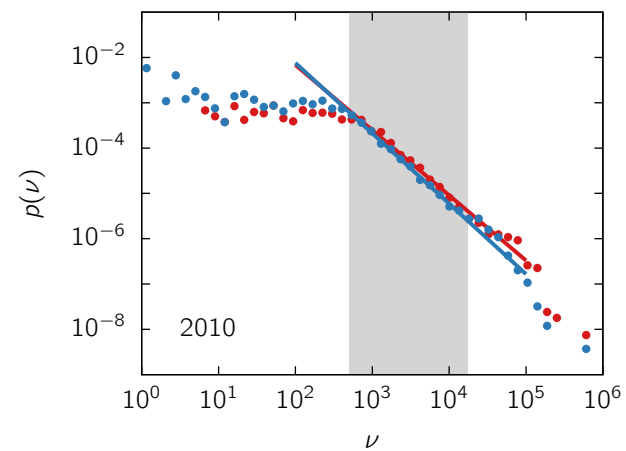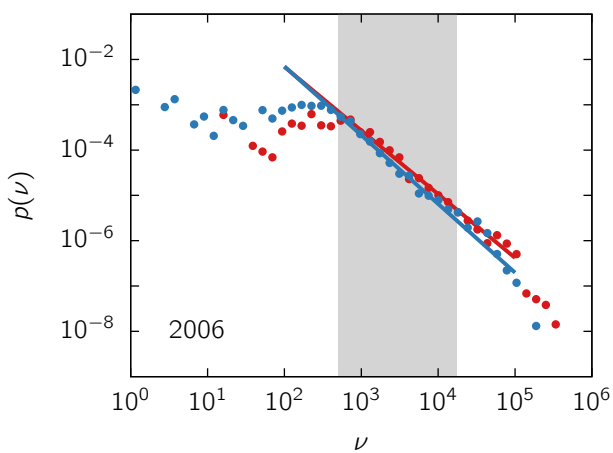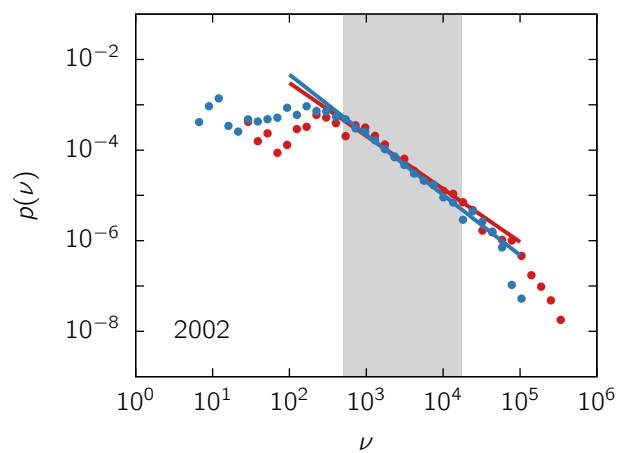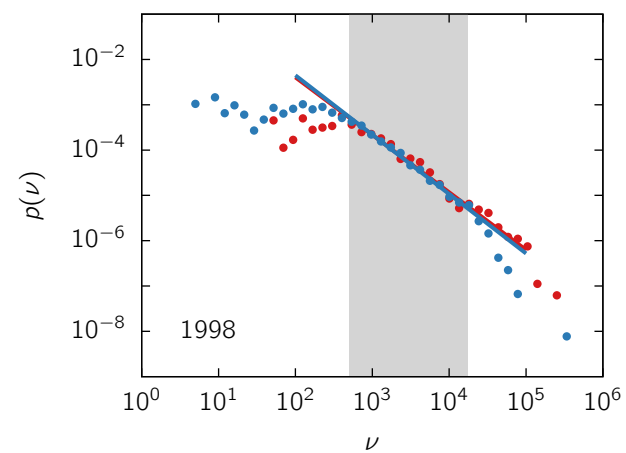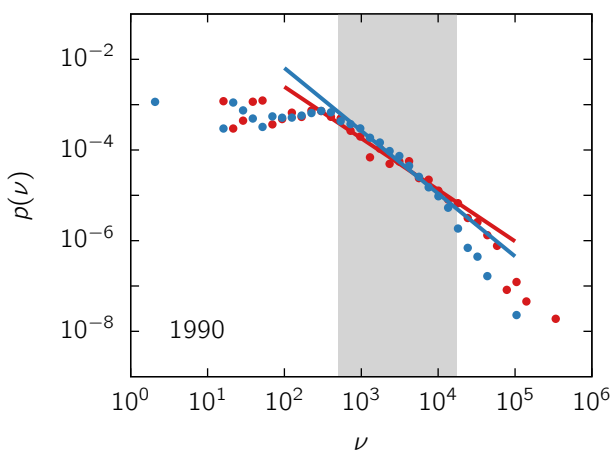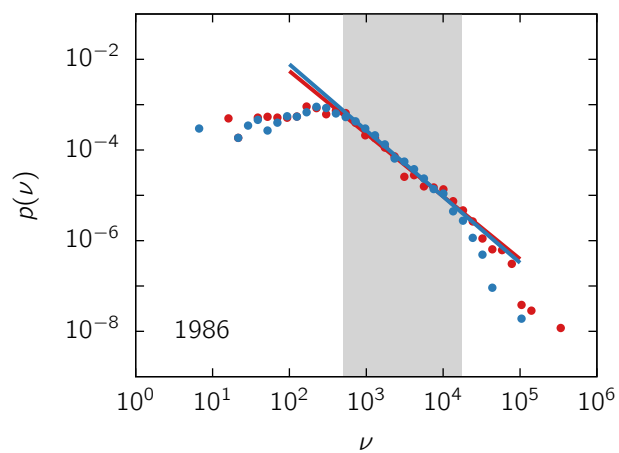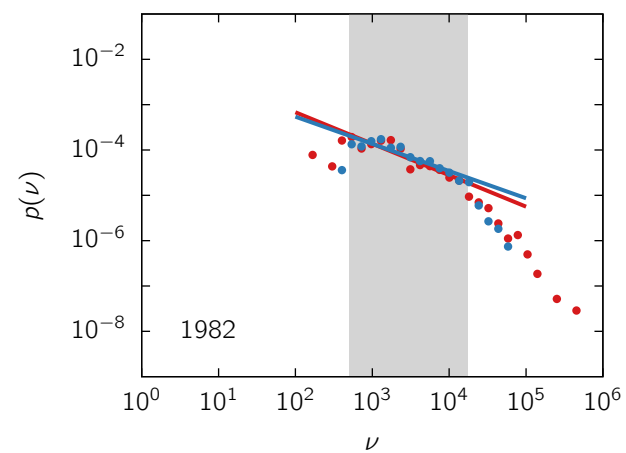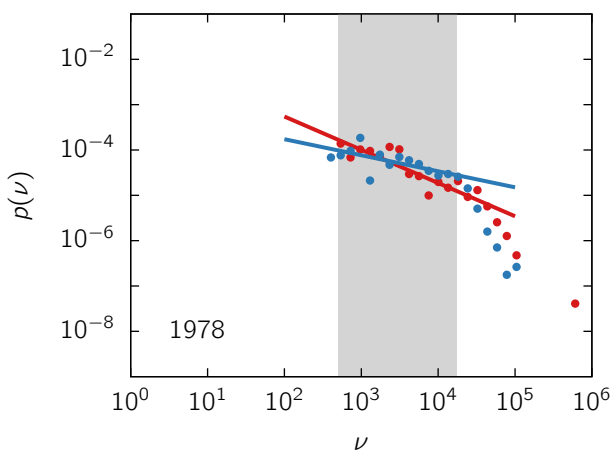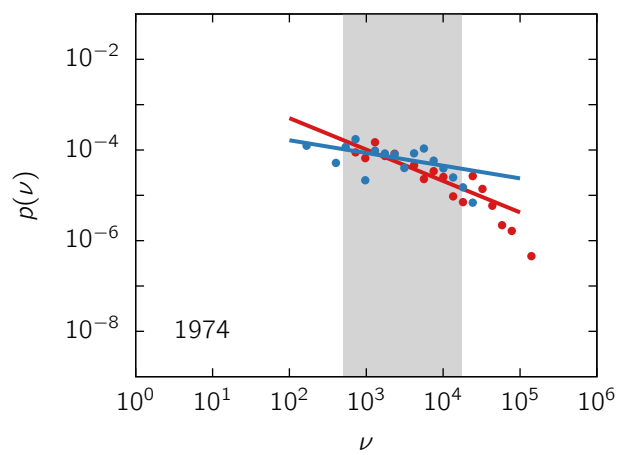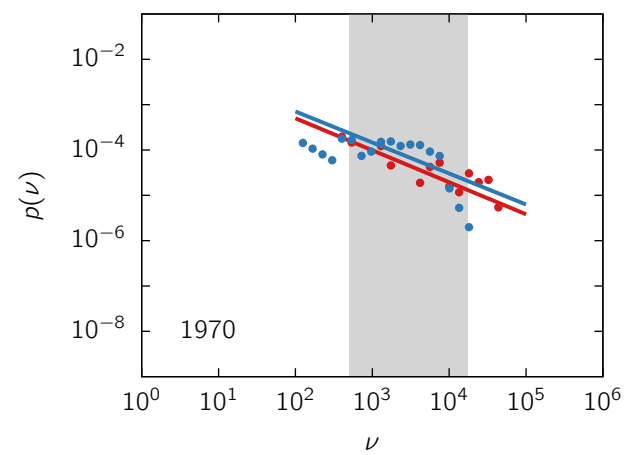

Supplement: S7 Fig — (PDF) [file pone.0137732.s009.pdf]

- Federal Deputies

- State Deputies

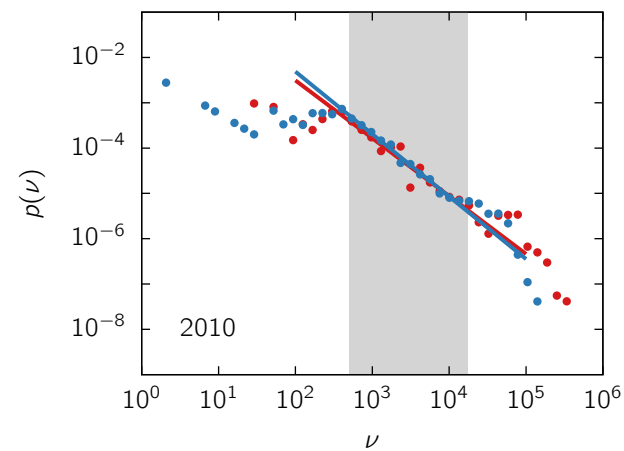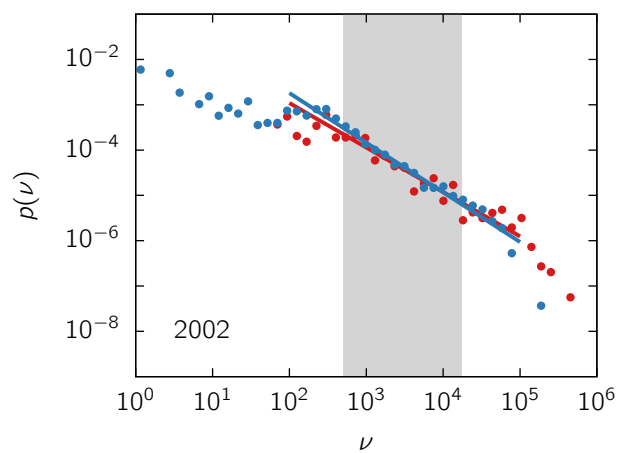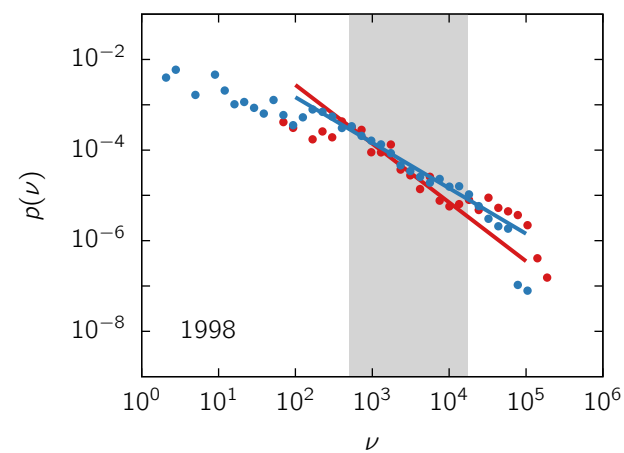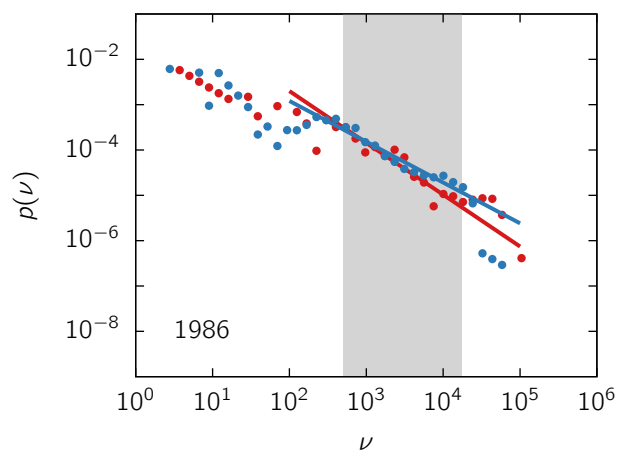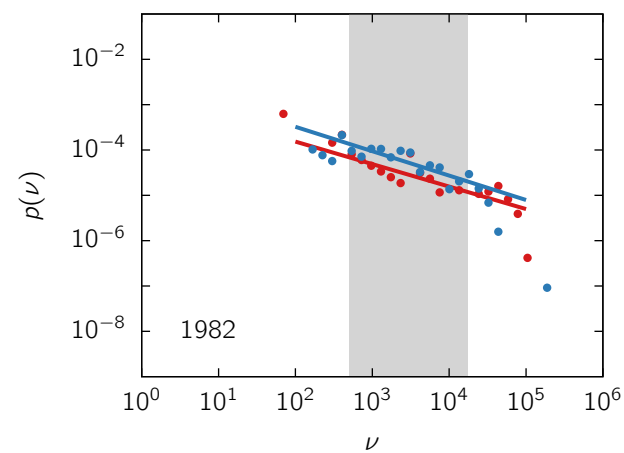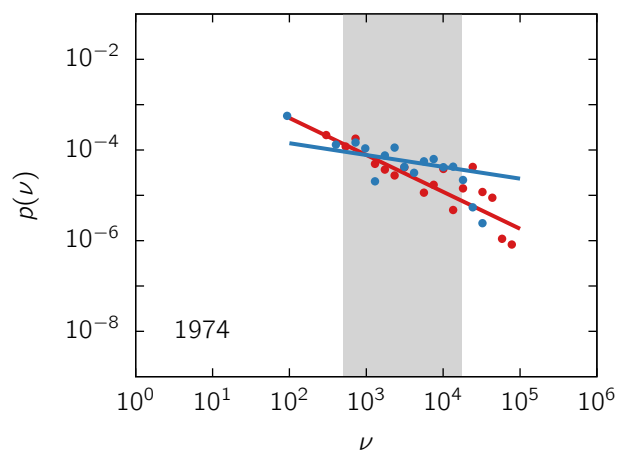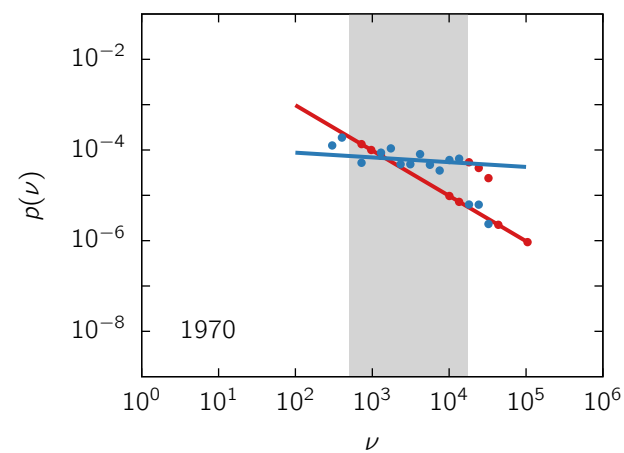

Supplement: S8 Fig — (PDF) [file pone.0137732.s010.pdf]

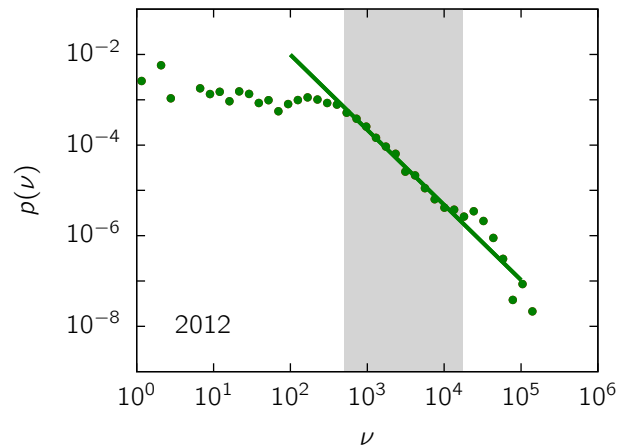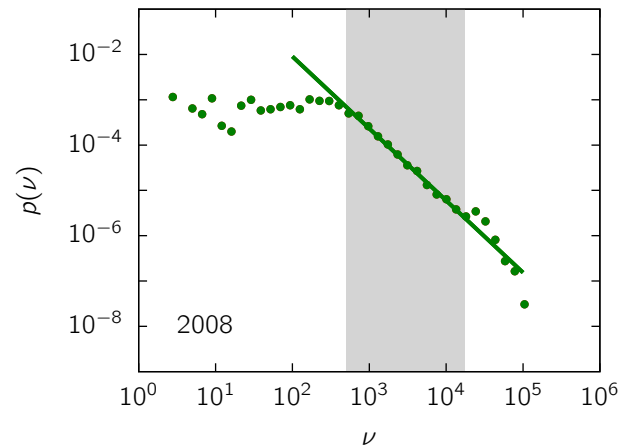

- SP - Capital
- City Councillors

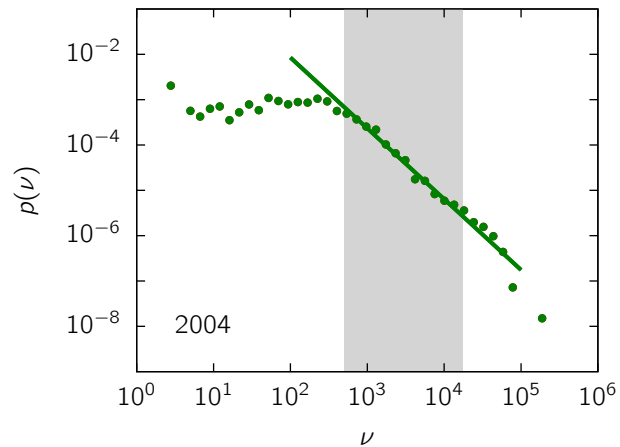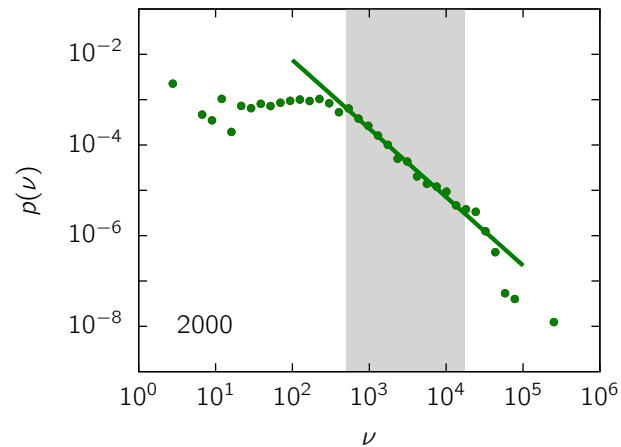

Supplement: S9 Fig — (PDF) [file pone.0137732.s012.pdf]

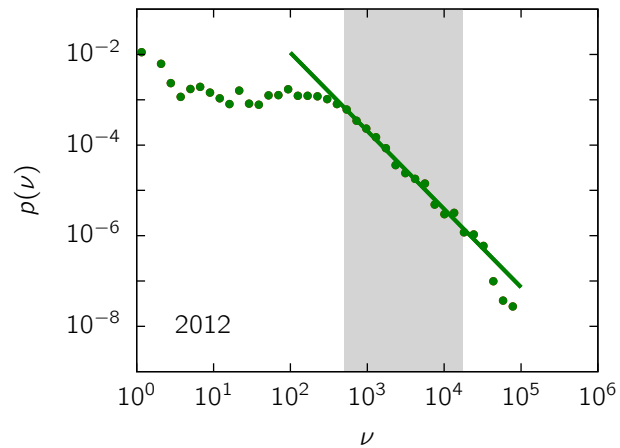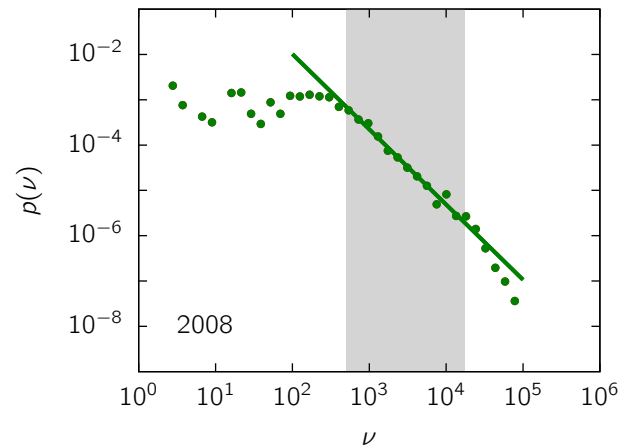

- RJ - Capital
- City Councillors

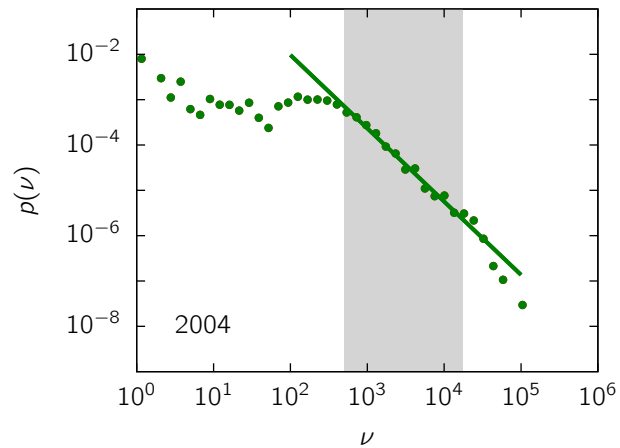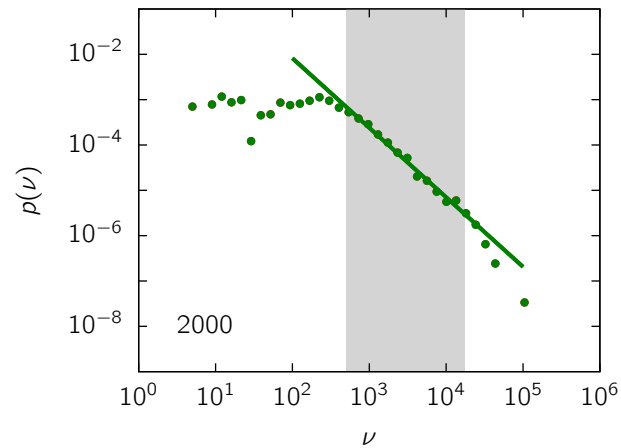

Supplement: S10 Fig — (PDF) [file pone.0137732.s013.pdf]

- SP - Capital
- Federal Deputies
  - State Deputies

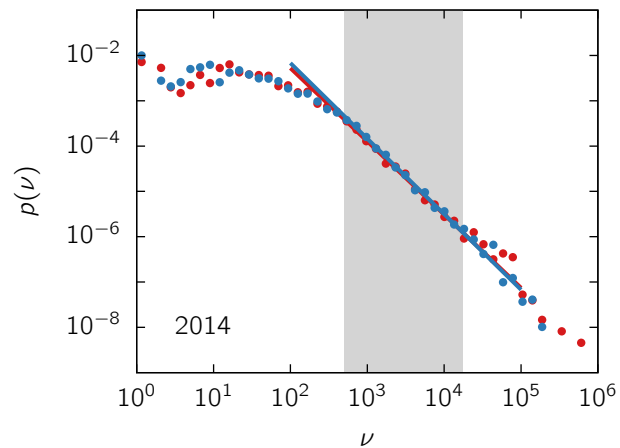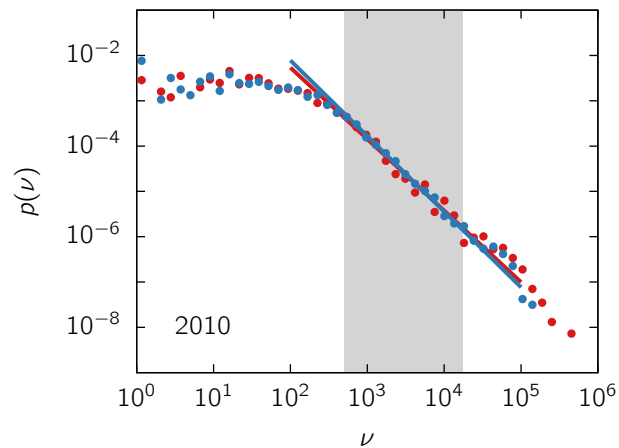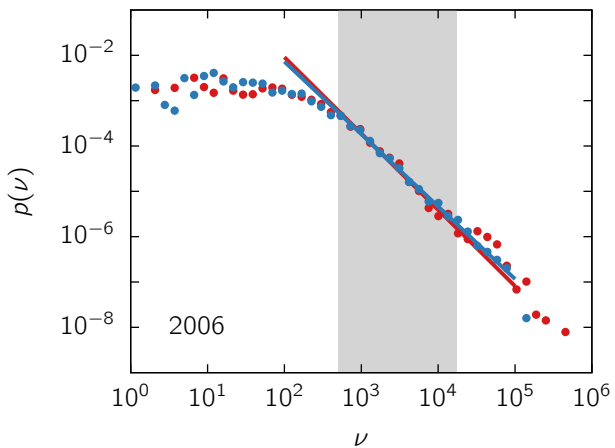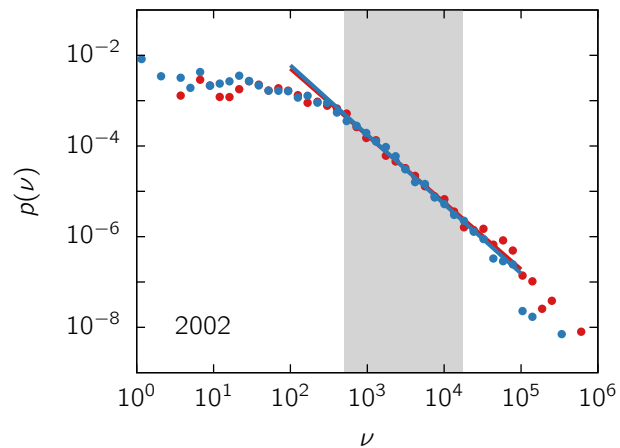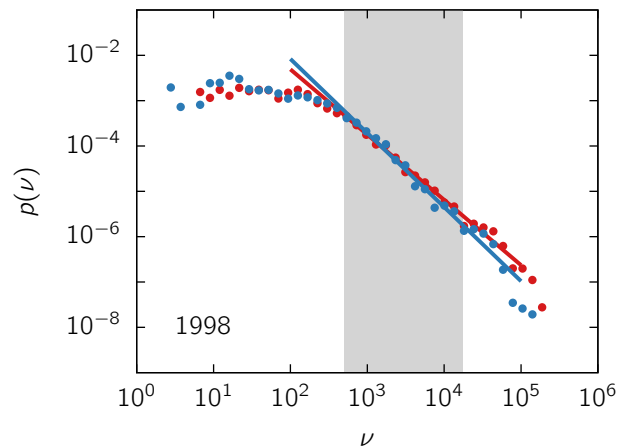

Supplement: S11 Fig — (PDF) [file pone.0137732.s015.pdf]

- RJ - Capital
- Federal Deputies
  - State Deputies

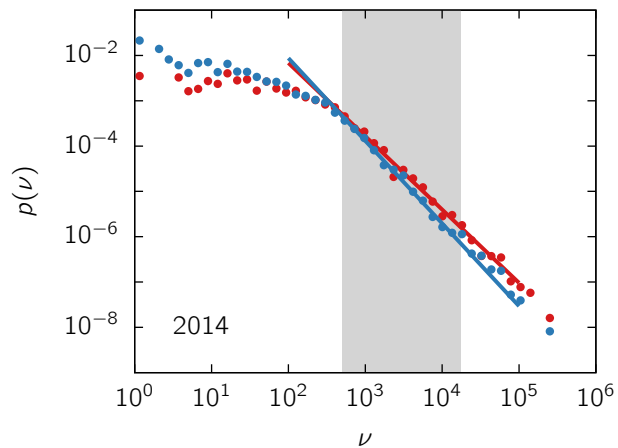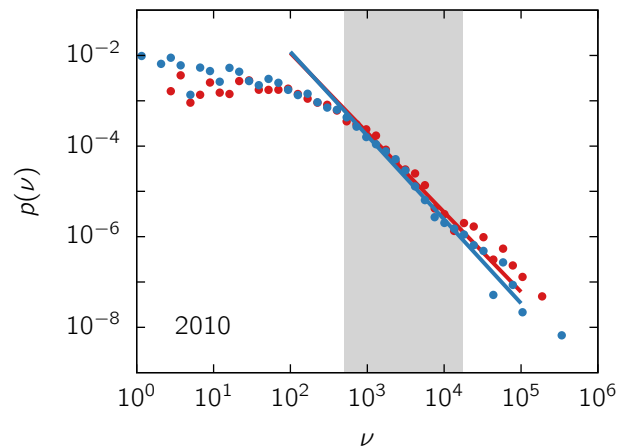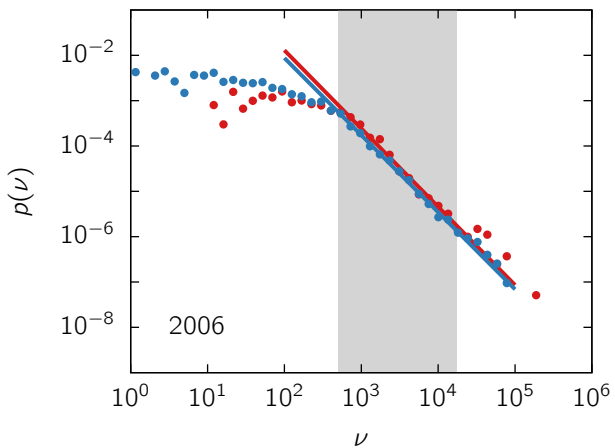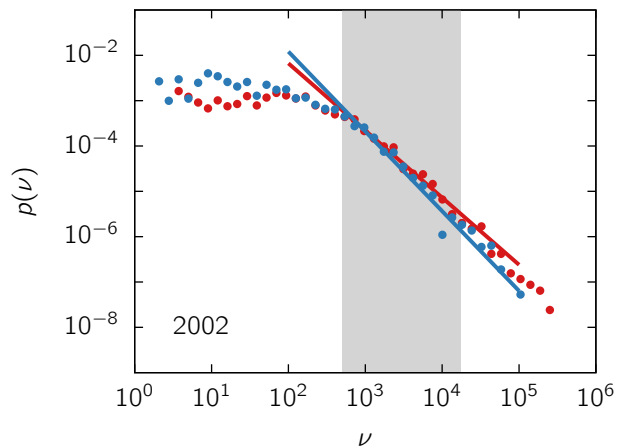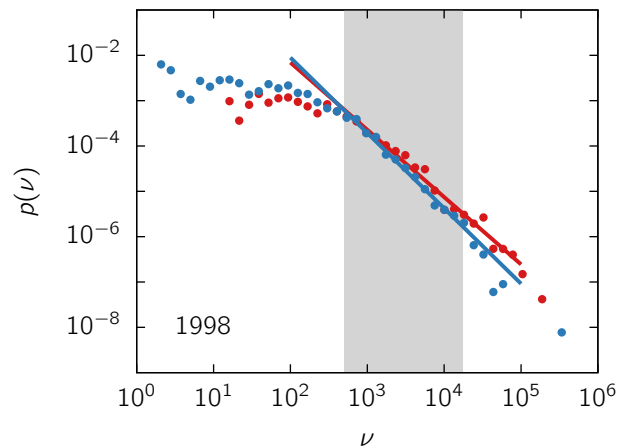

Supplement: S12 Fig — (PDF) [file pone.0137732.s016.pdf]

SP - Others  
• Federal Deputies  
• State Deputies

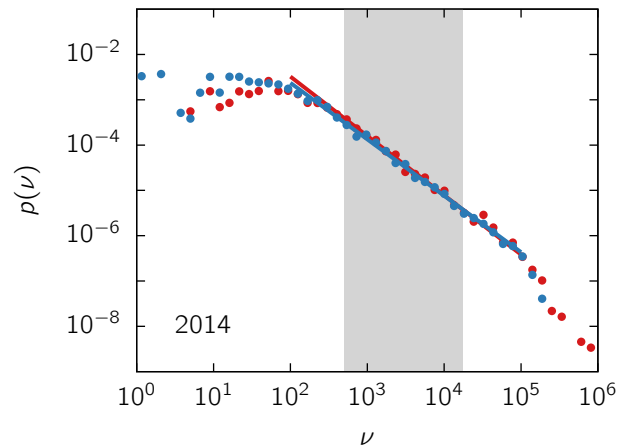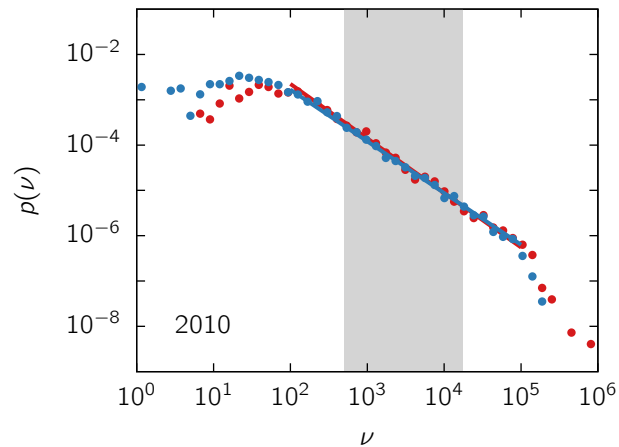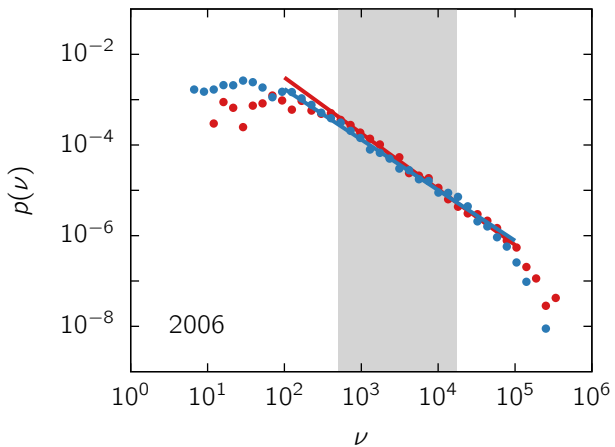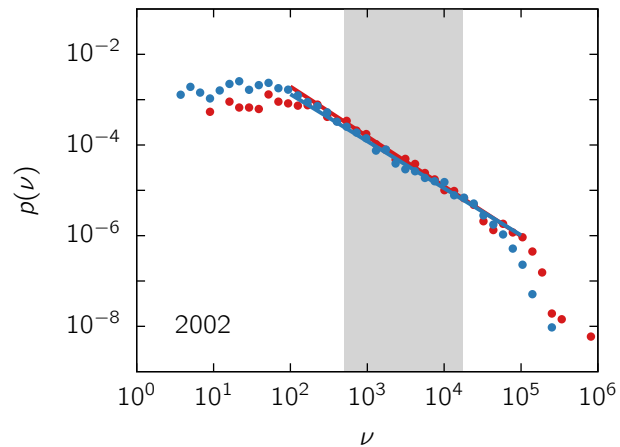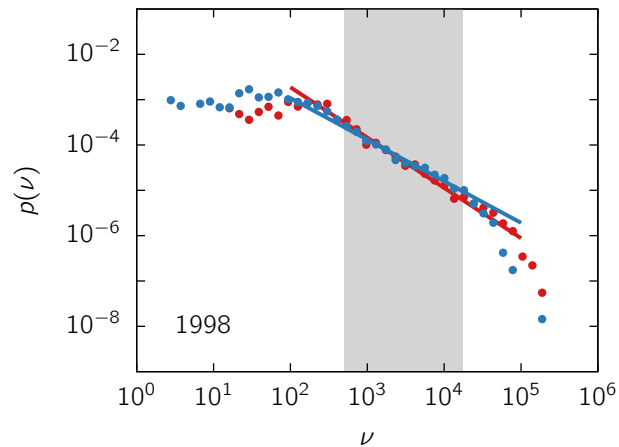

Supplement: S13 Fig — (PDF) [file pone.0137732.s017.pdf]

- RJ - Others
- Federal Deputies
  - State Deputies

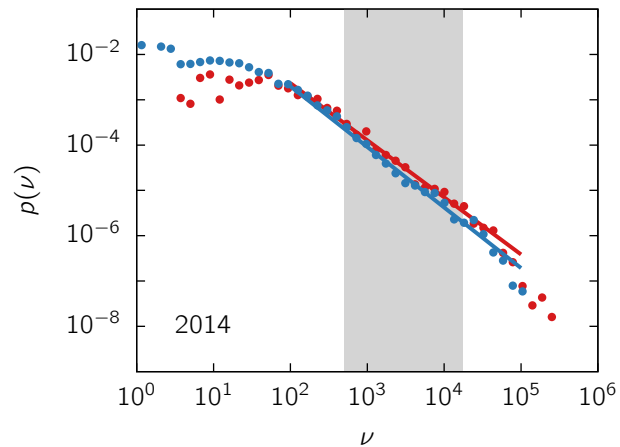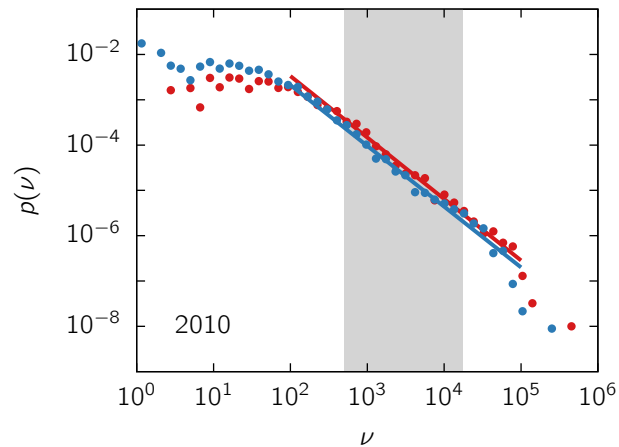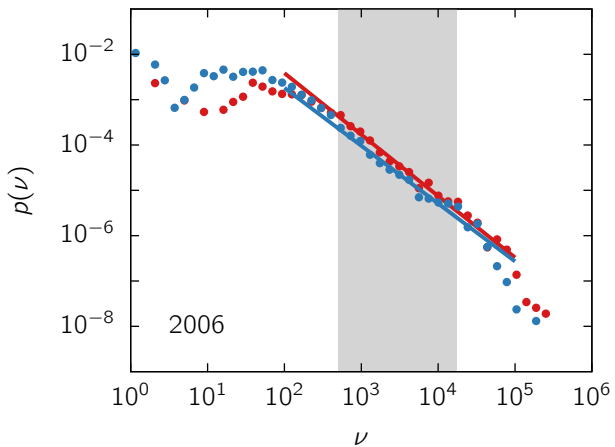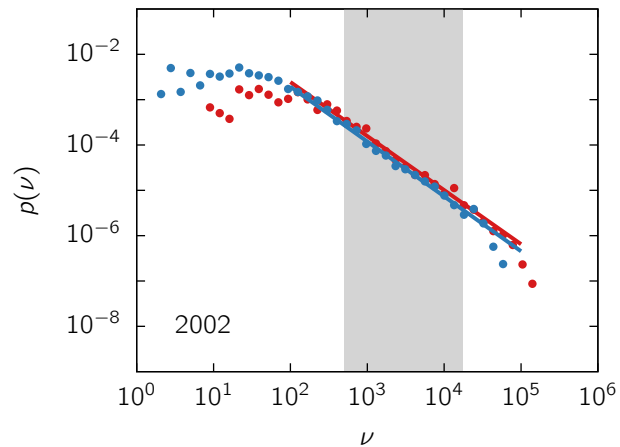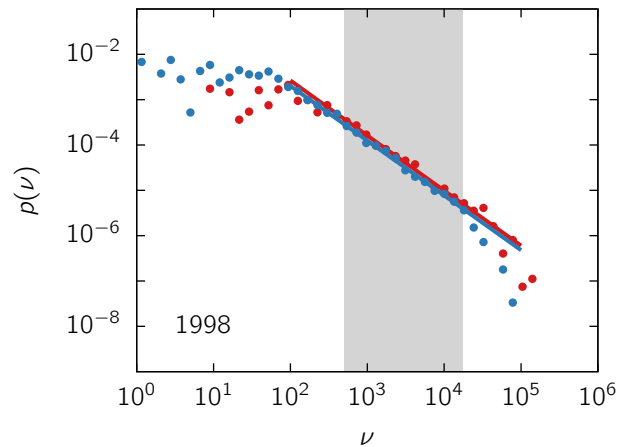

Supplement: S14 Fig — (PDF) [file pone.0137732.s018.pdf]

AC  
• Federal Deputies

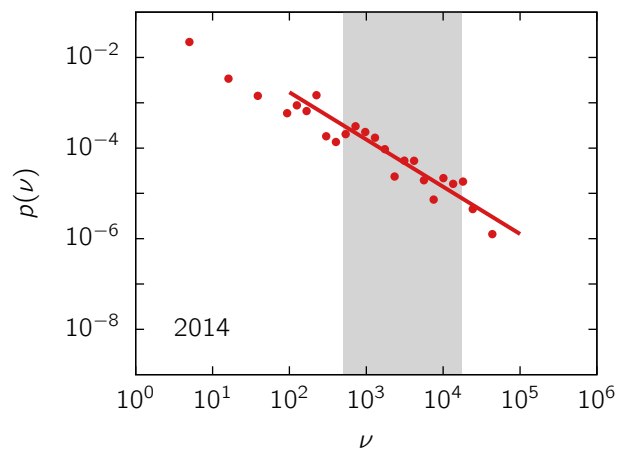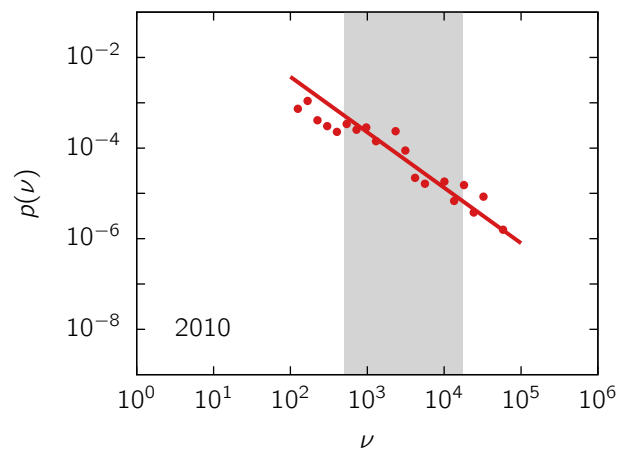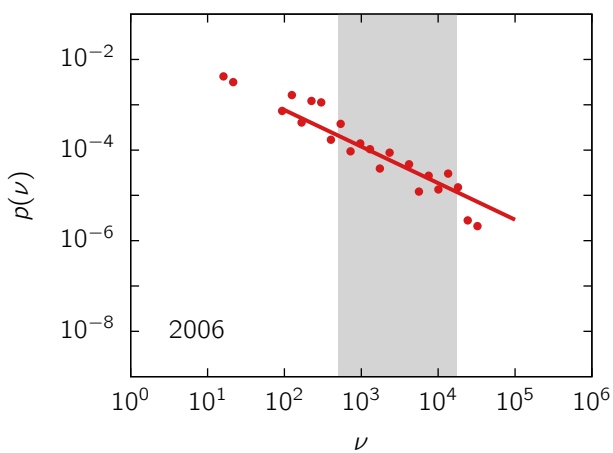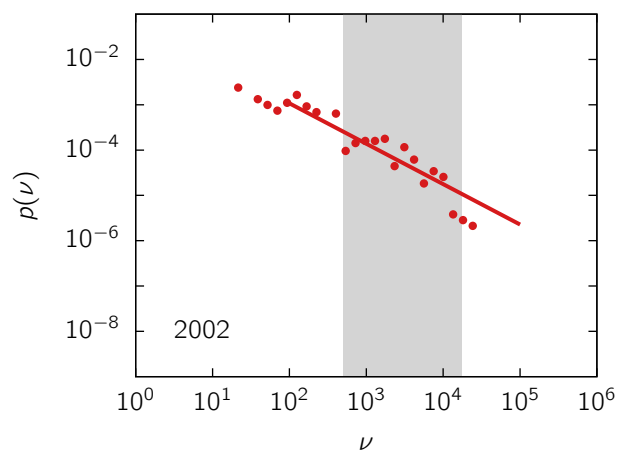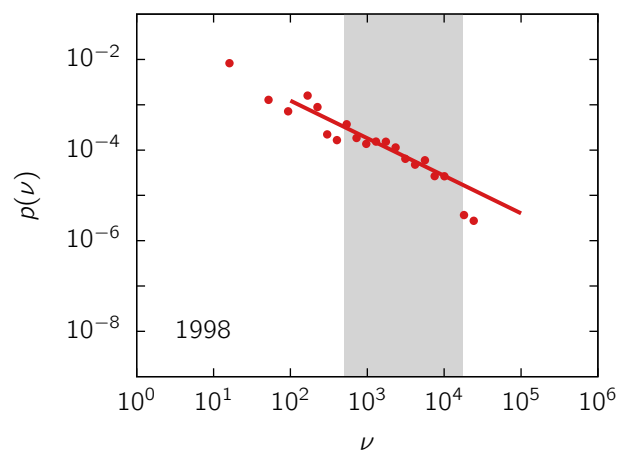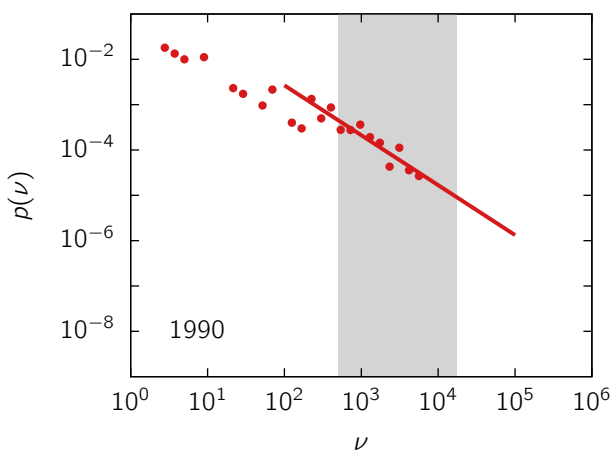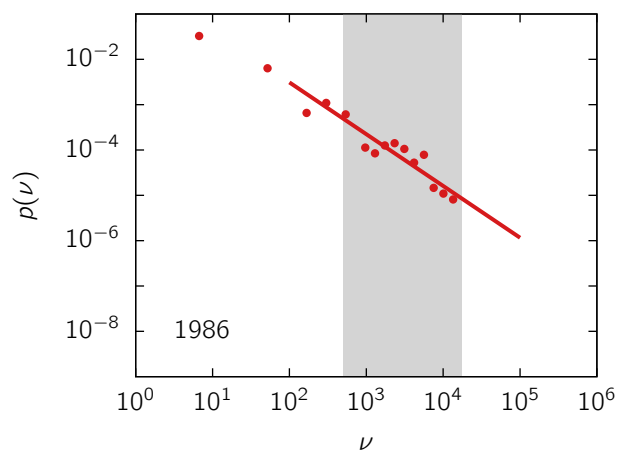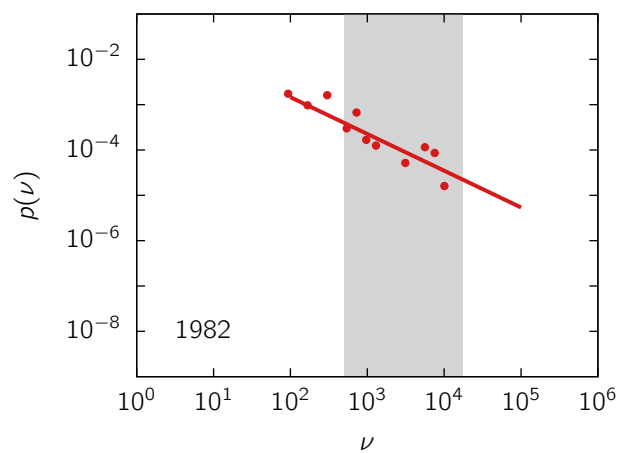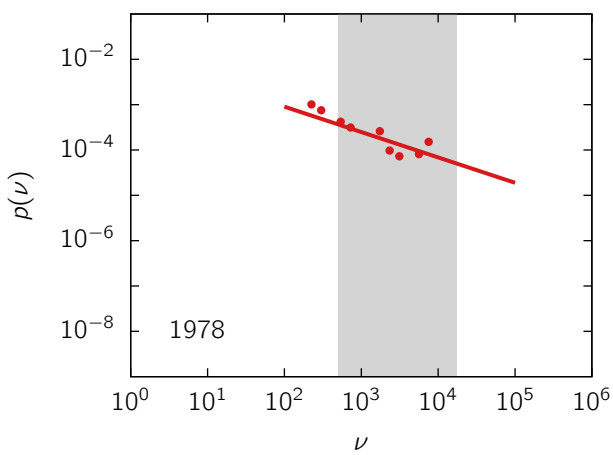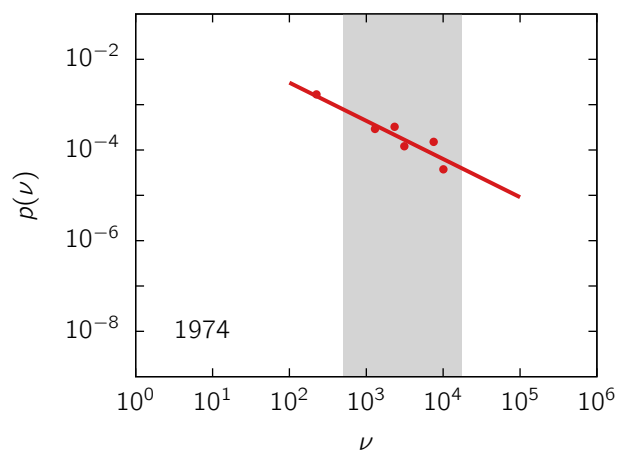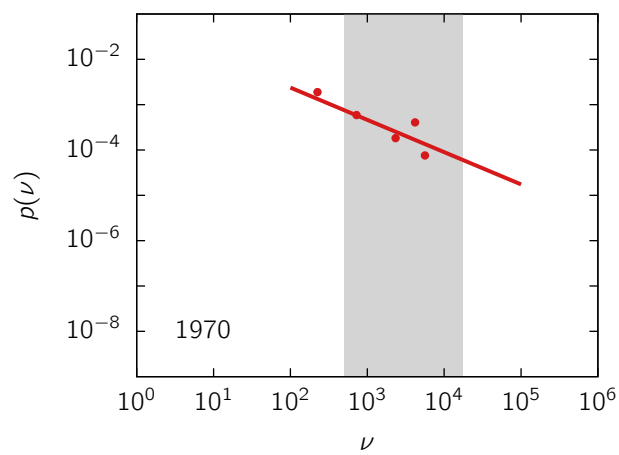

Supplement: S15 Fig — (PDF) [file pone.0137732.s021.pdf]

AL  
• Federal Deputies

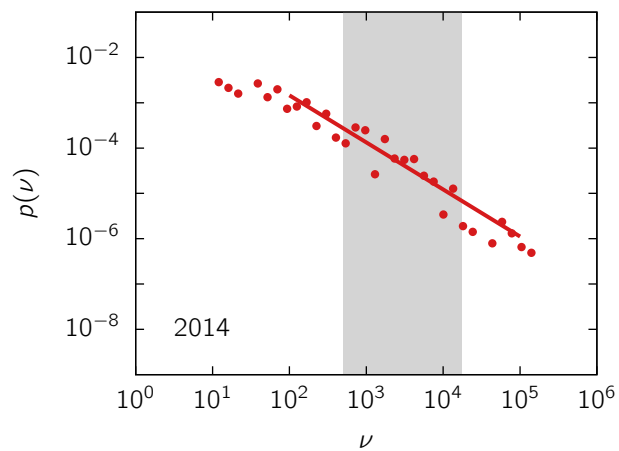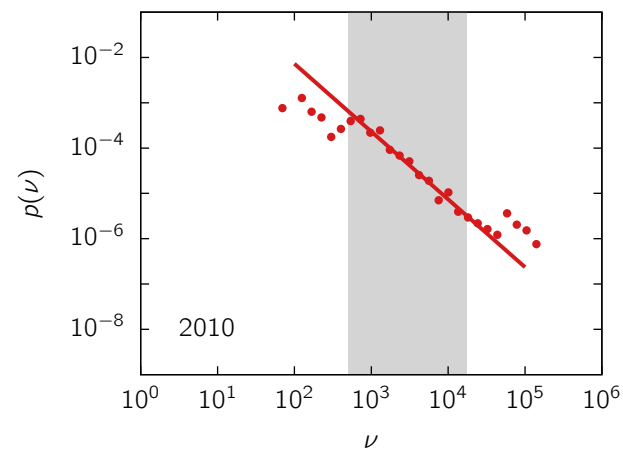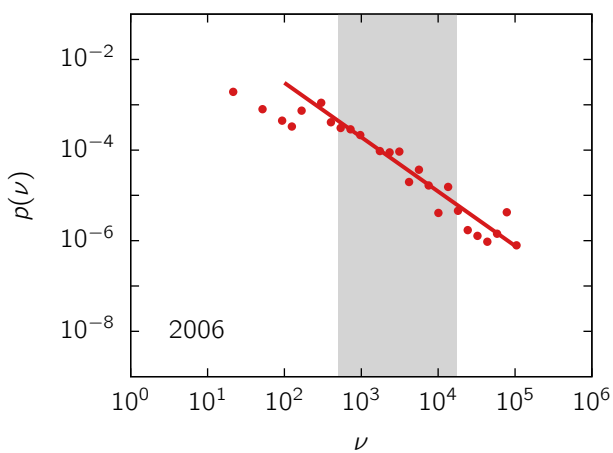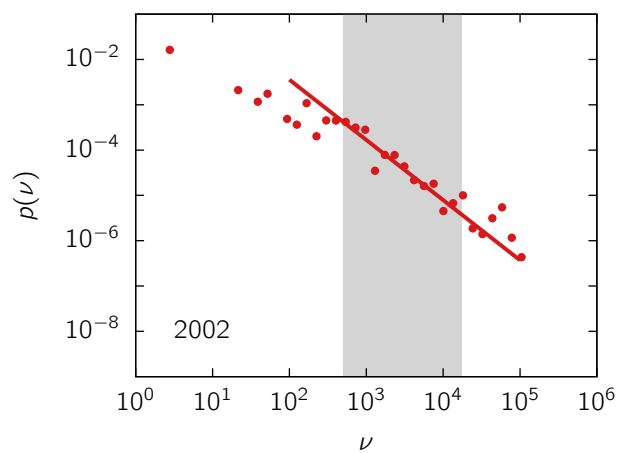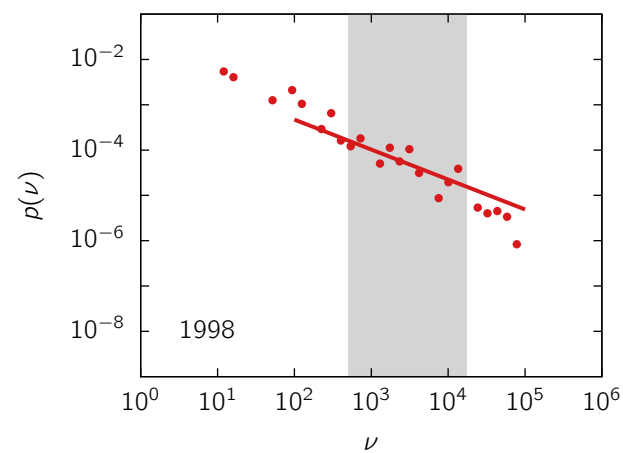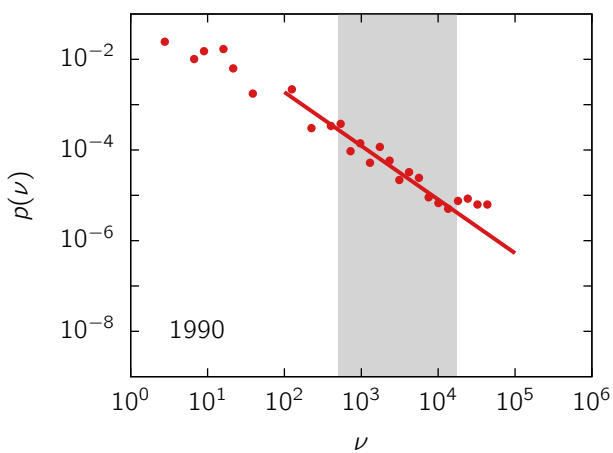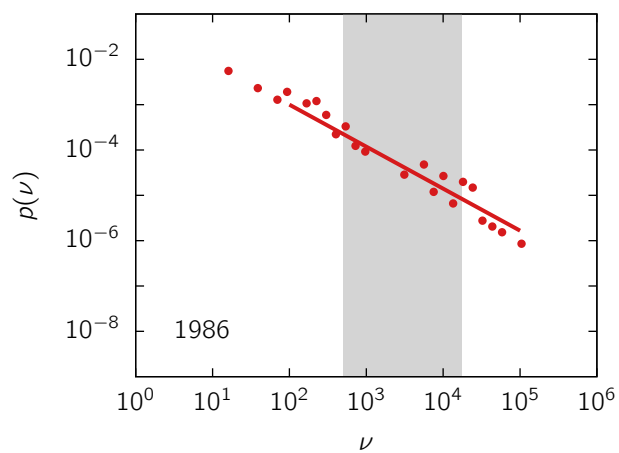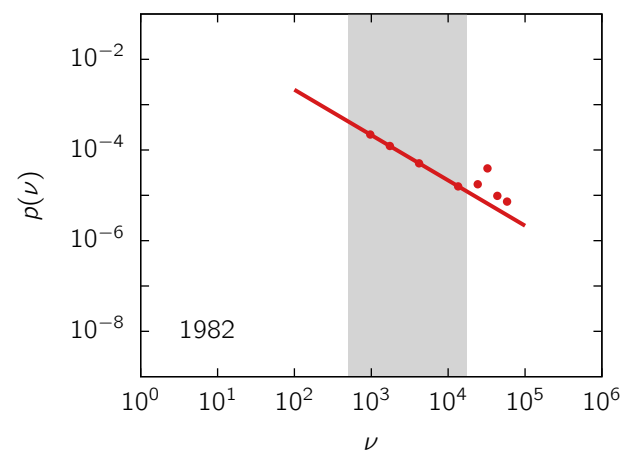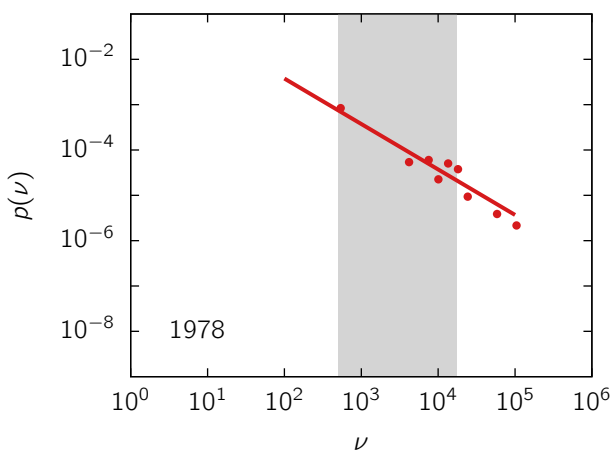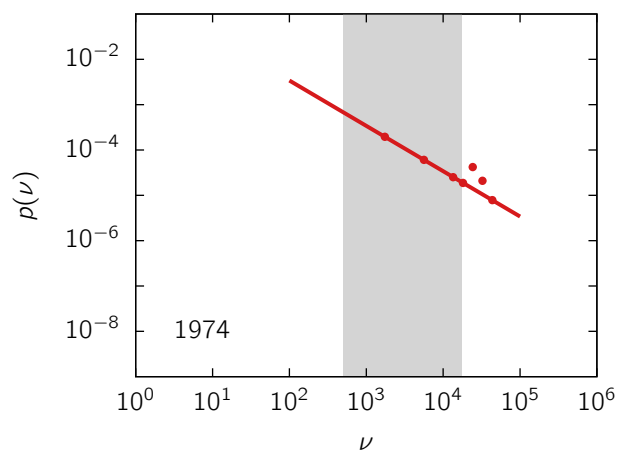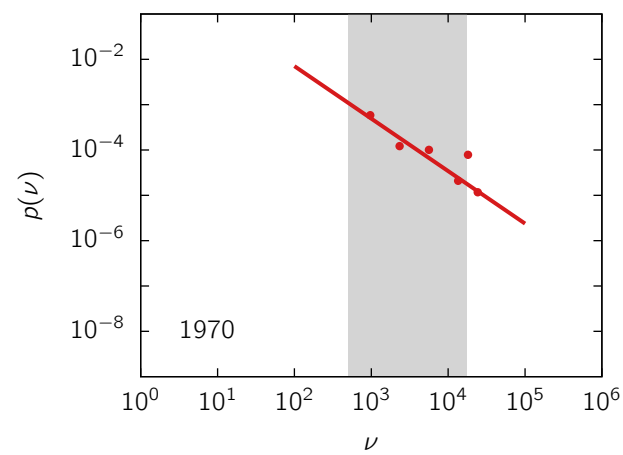

Supplement: S16 Fig — (PDF) [file pone.0137732.s022.pdf]

AP  
• Federal Deputies

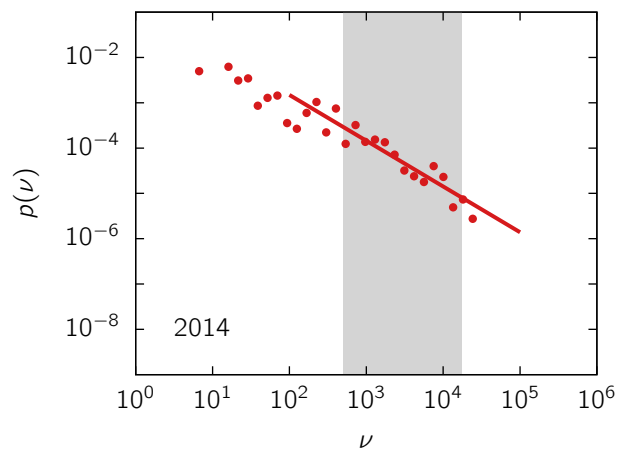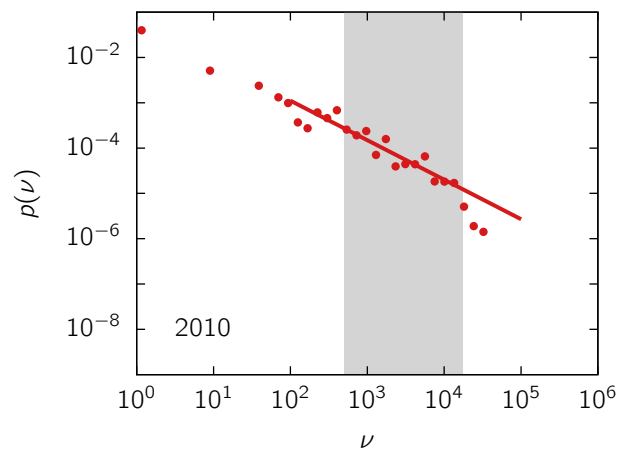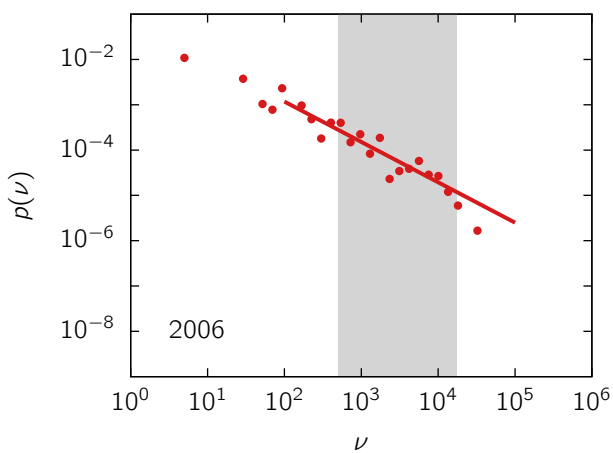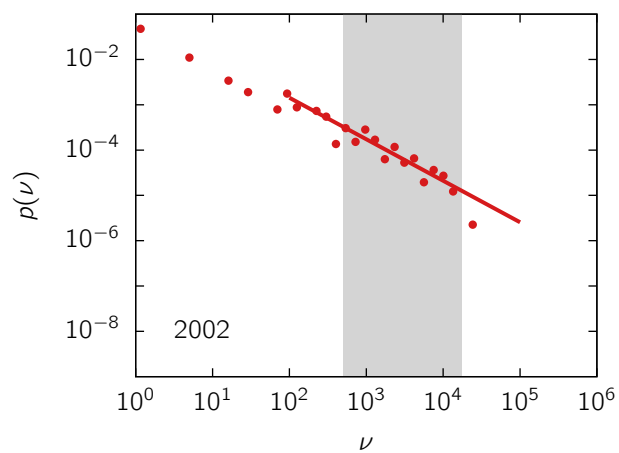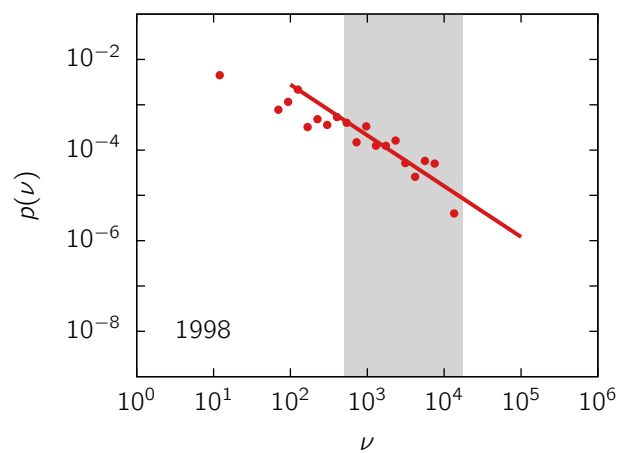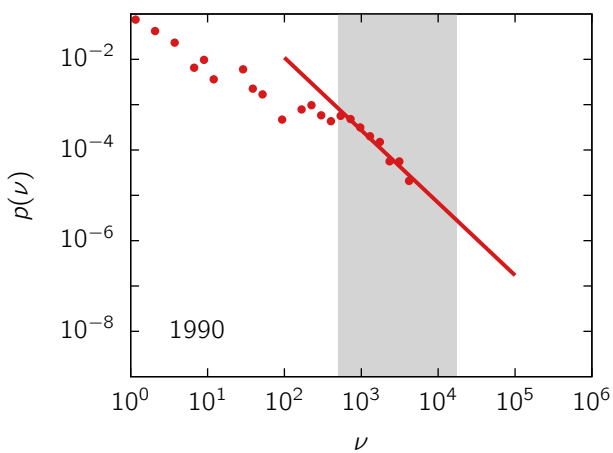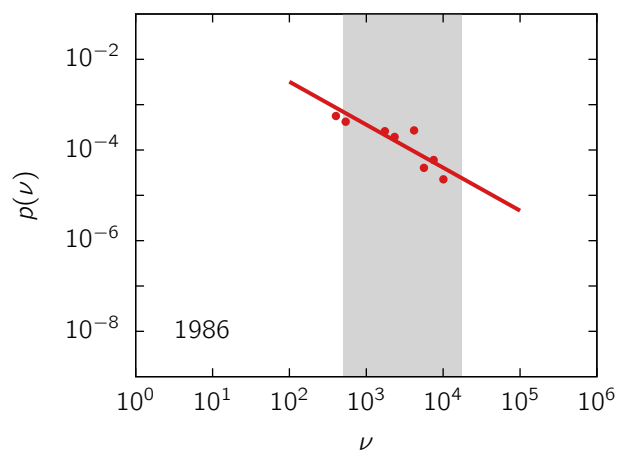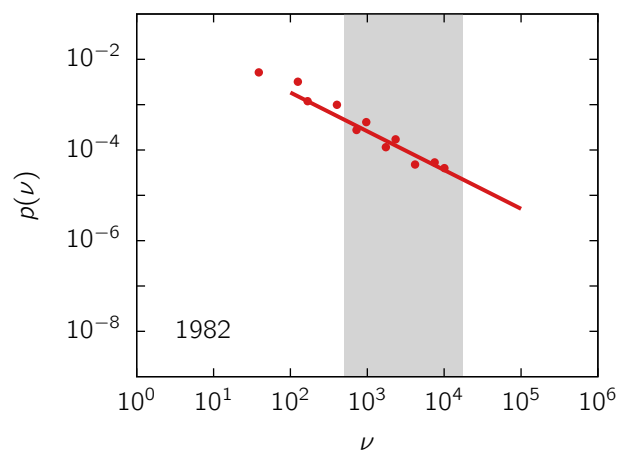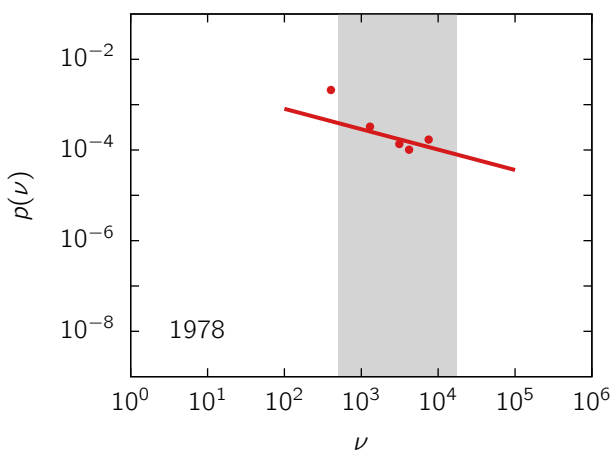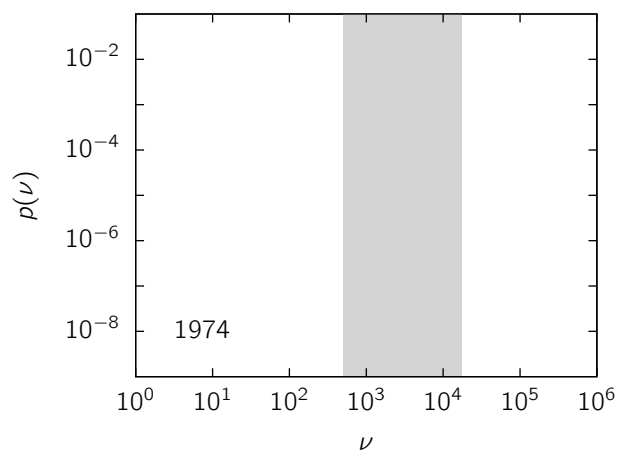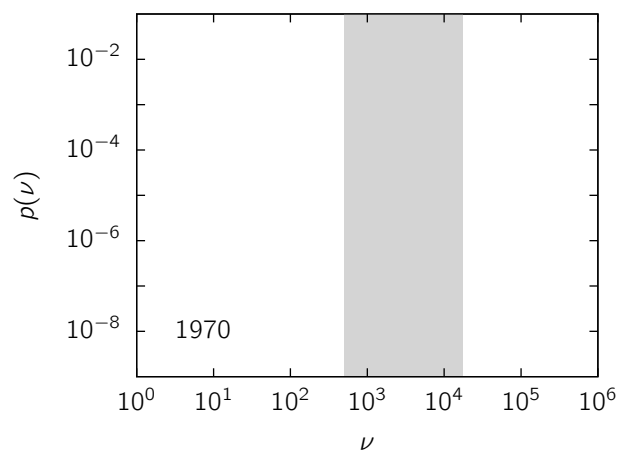

Supplement: S17 Fig — (PDF) [file pone.0137732.s023.pdf]

AM  
• Federal Deputies

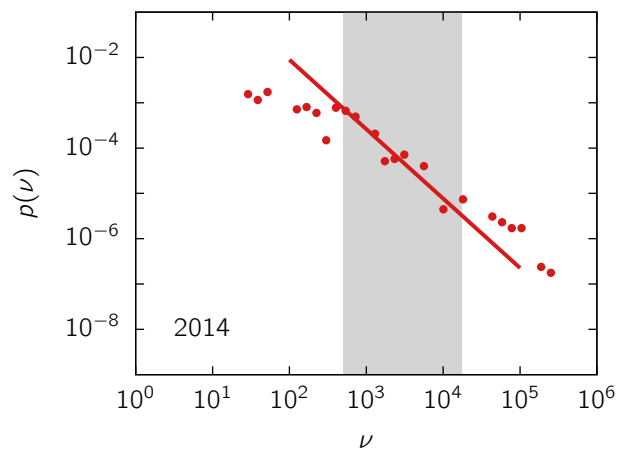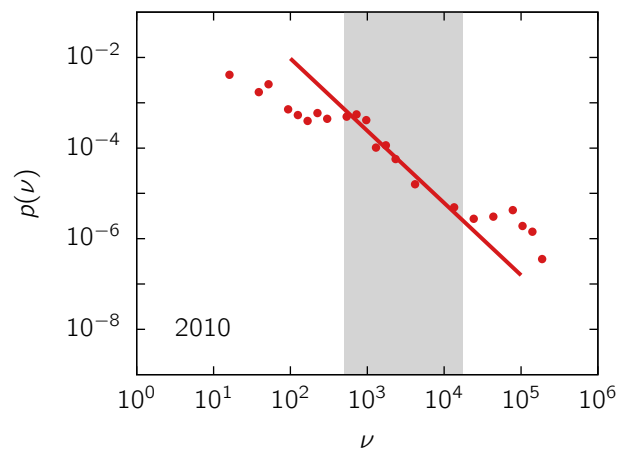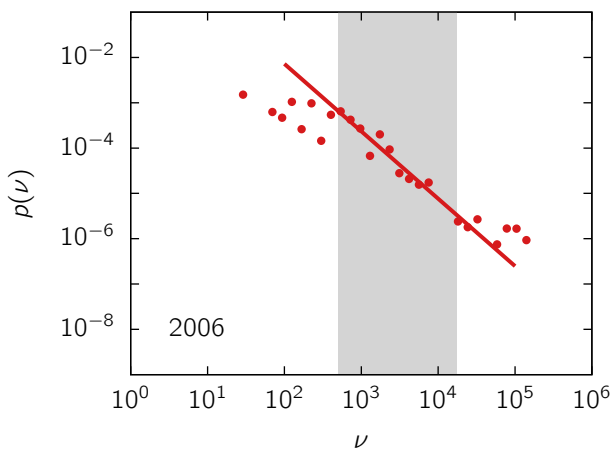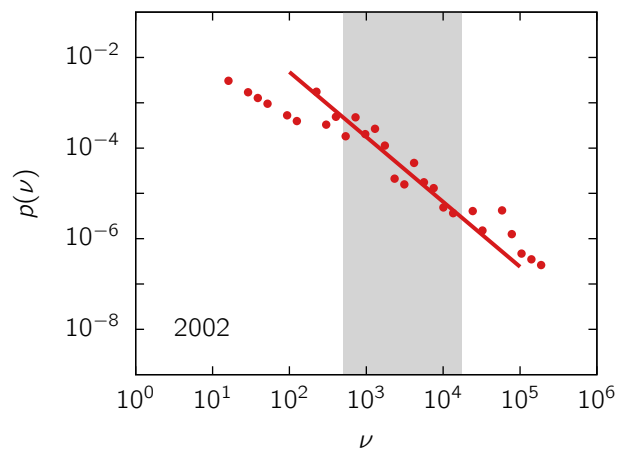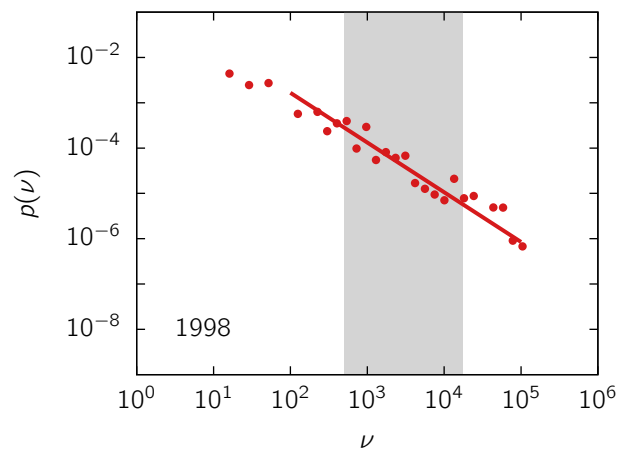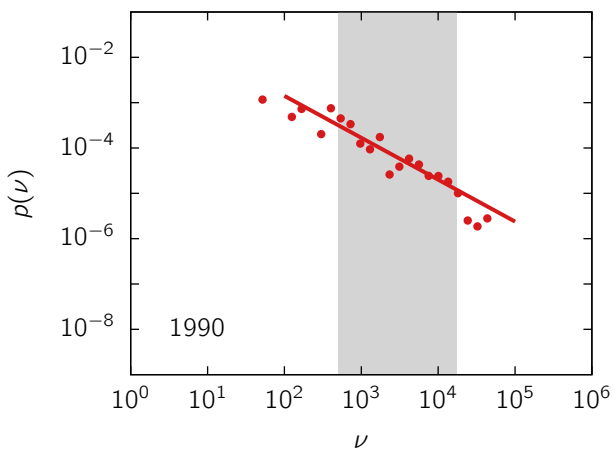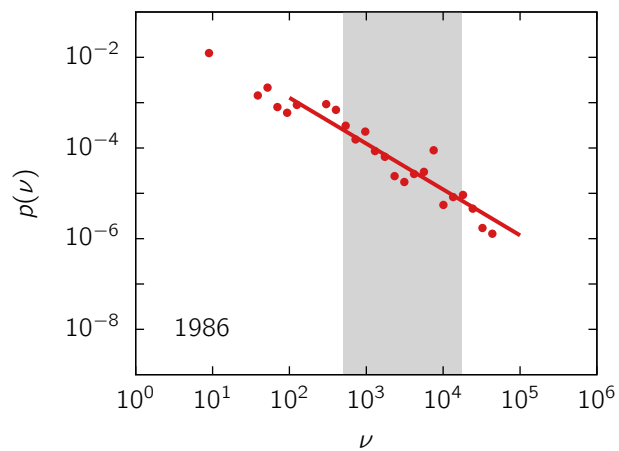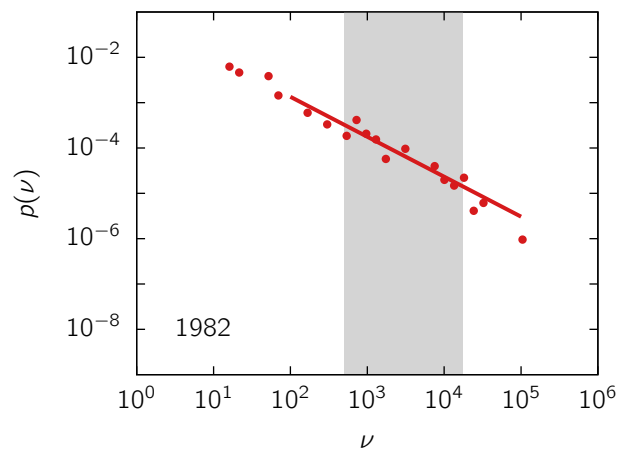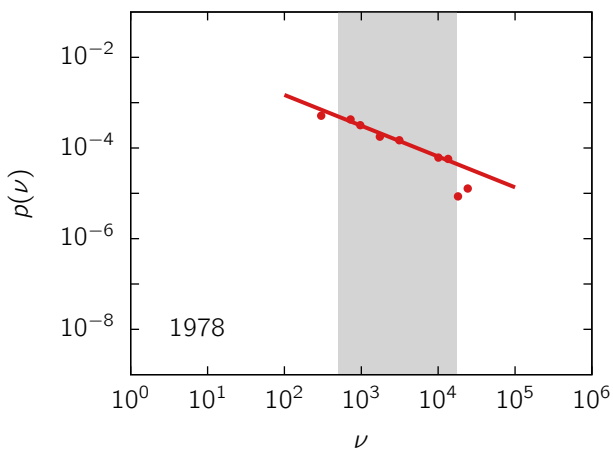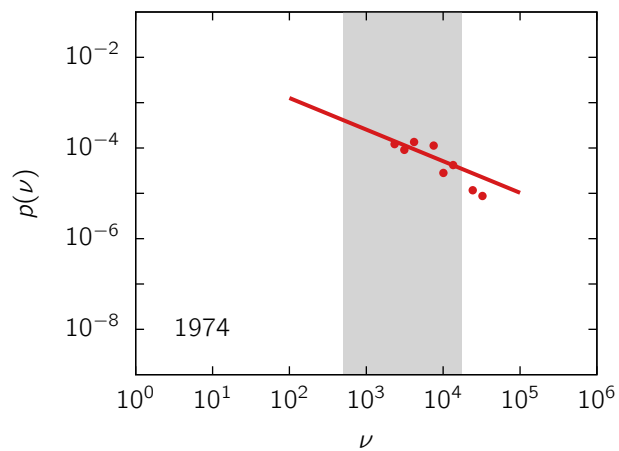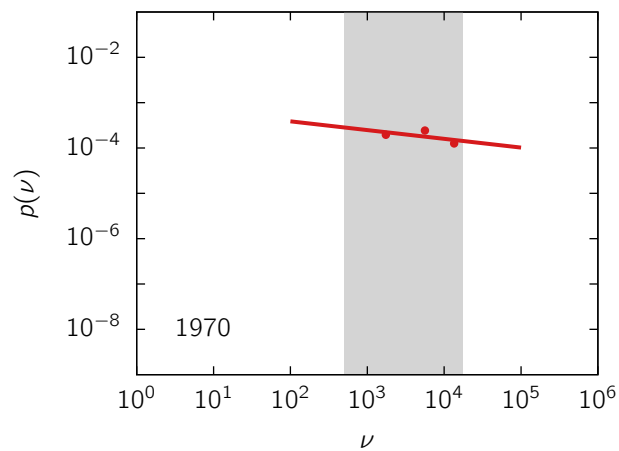

Supplement: S18 Fig — (PDF) [file pone.0137732.s024.pdf]

CE  
• Federal Deputies

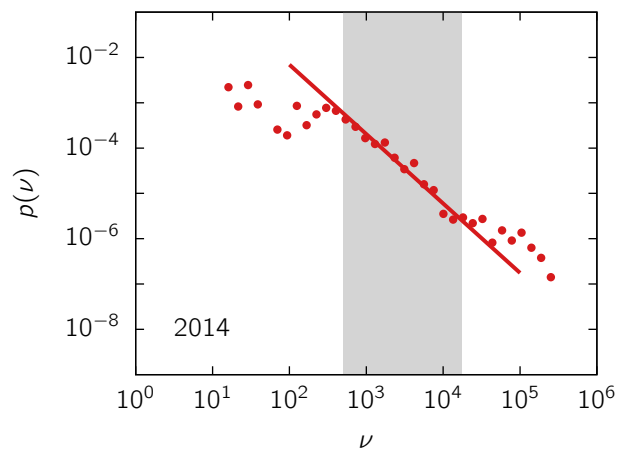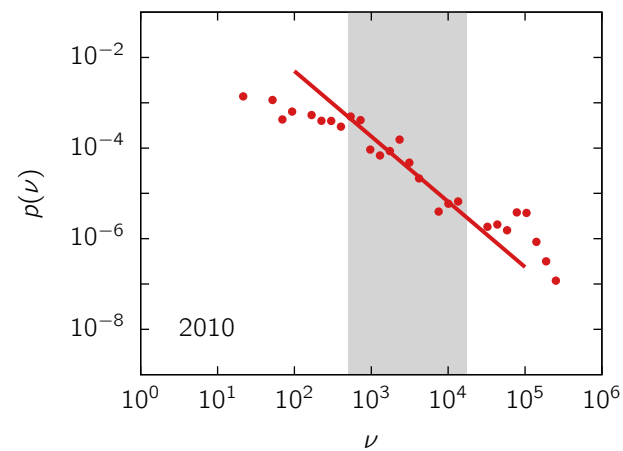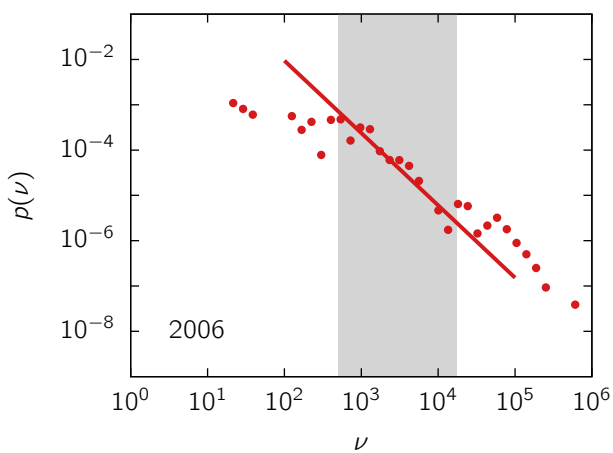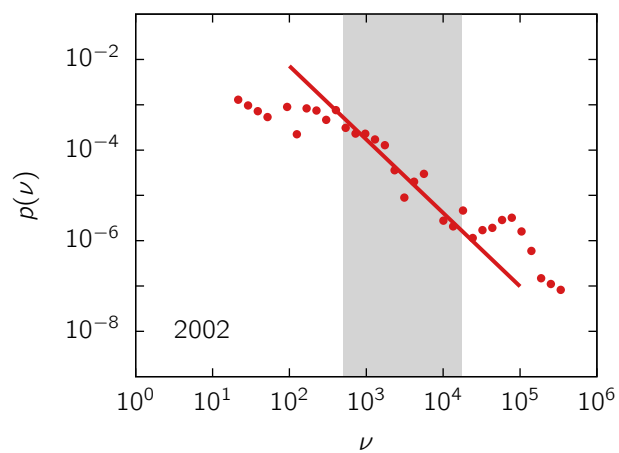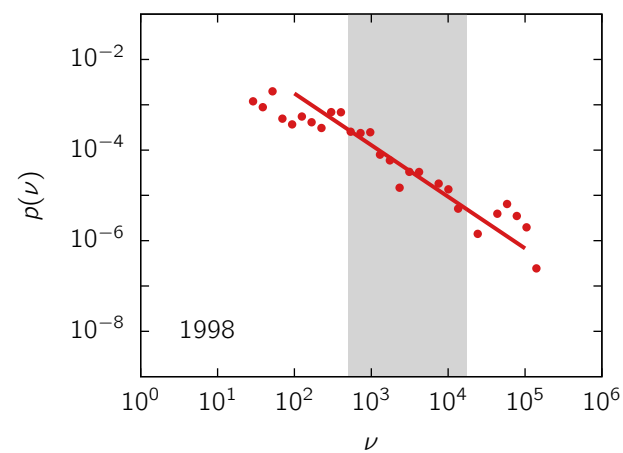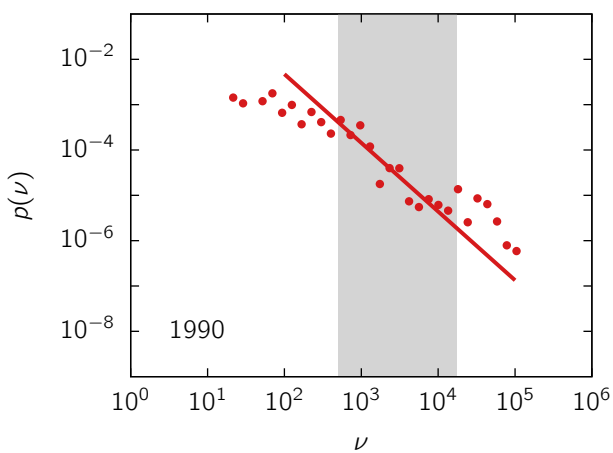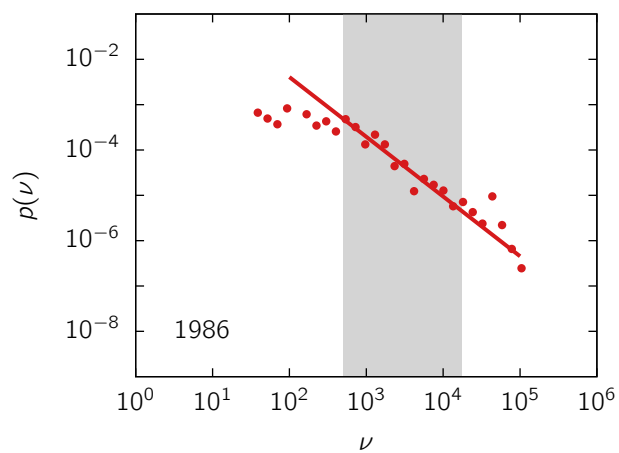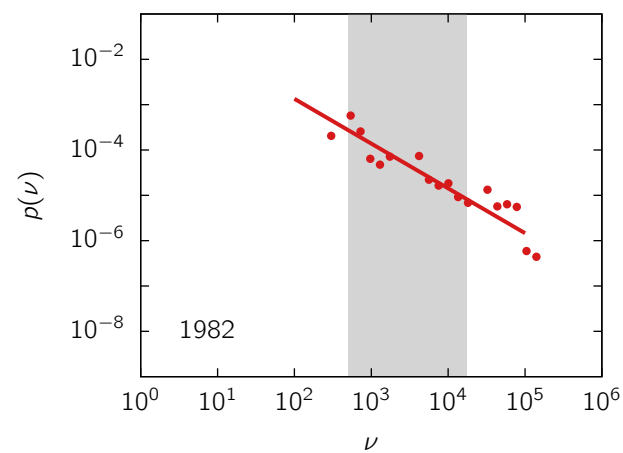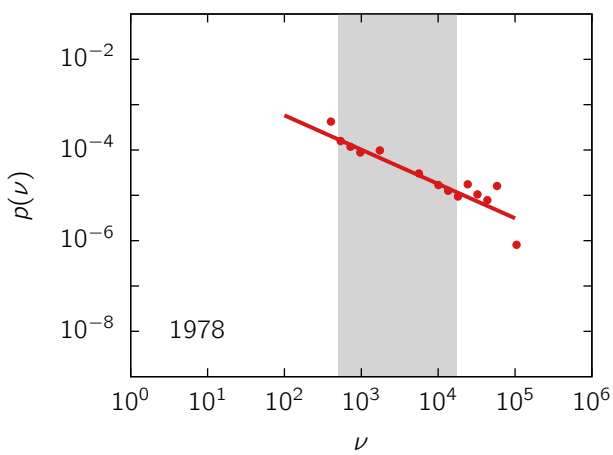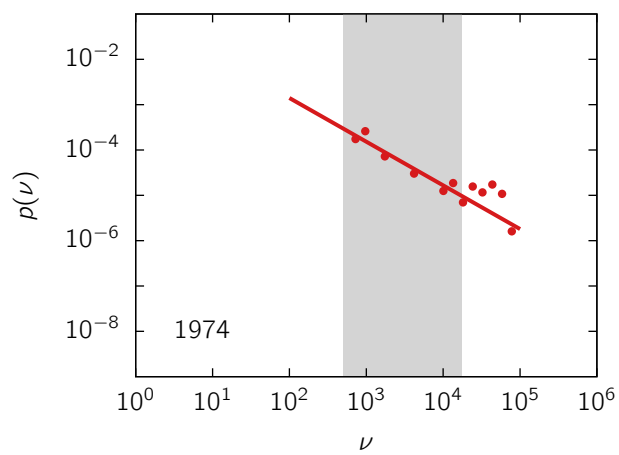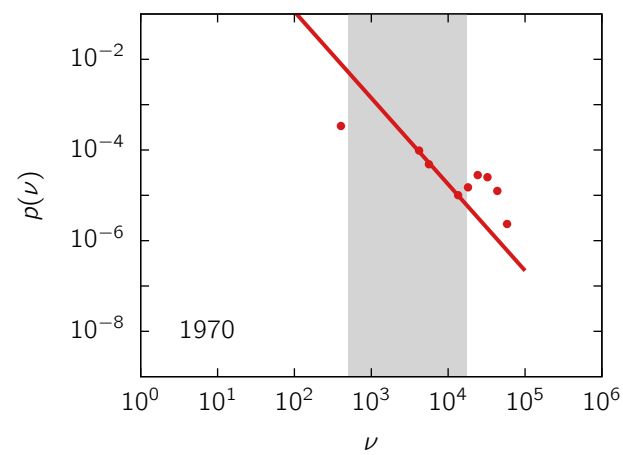

Supplement: S19 Fig — (PDF) [file pone.0137732.s025.pdf]

- ES
- Federal Deputies

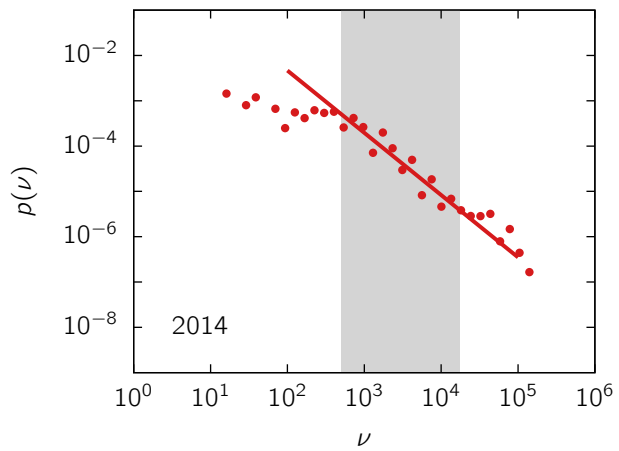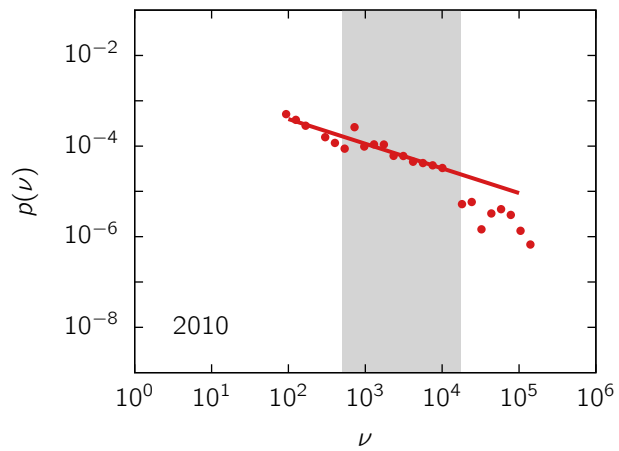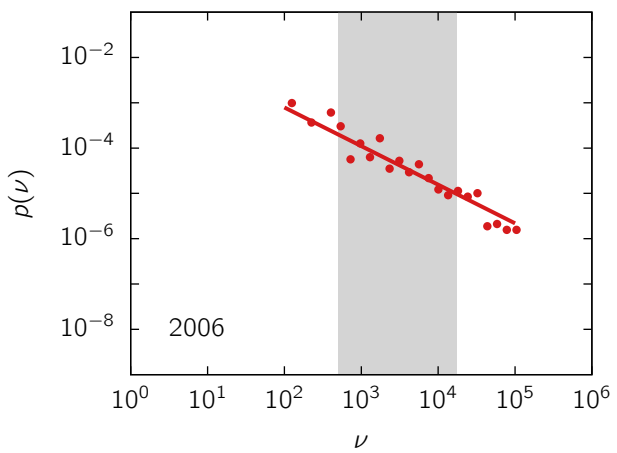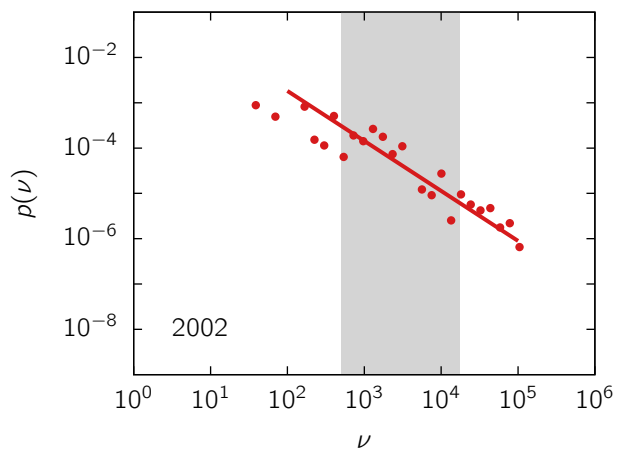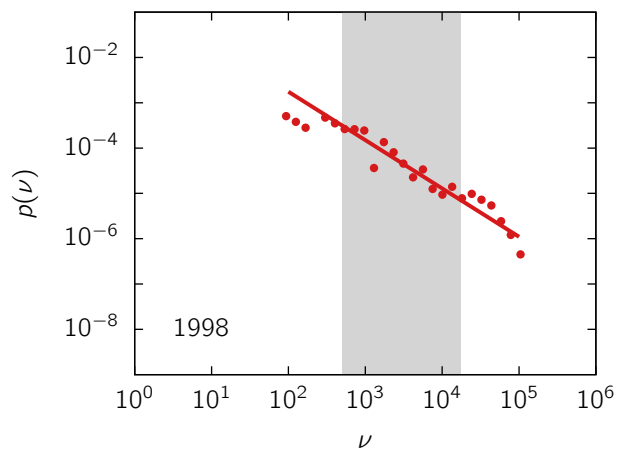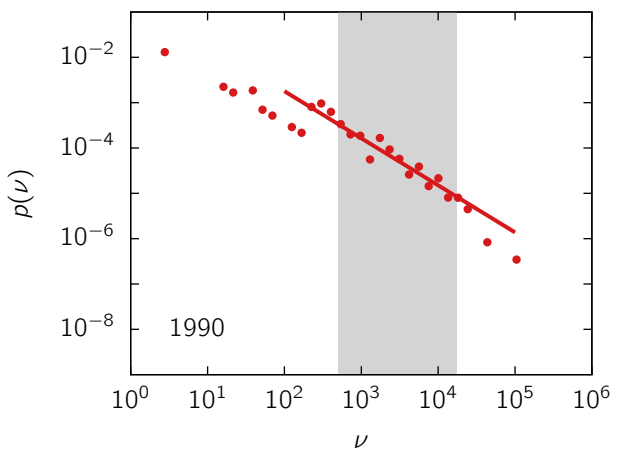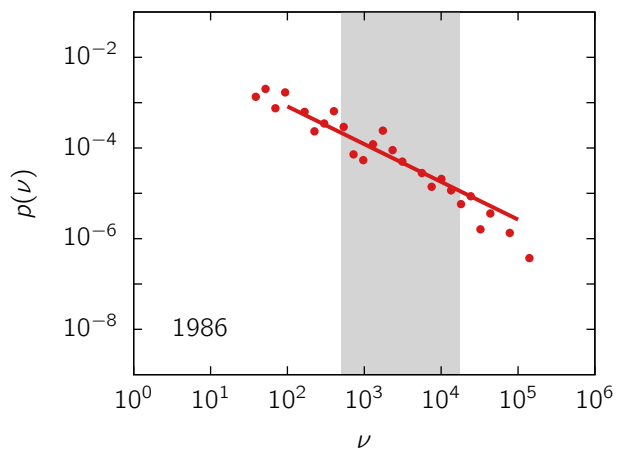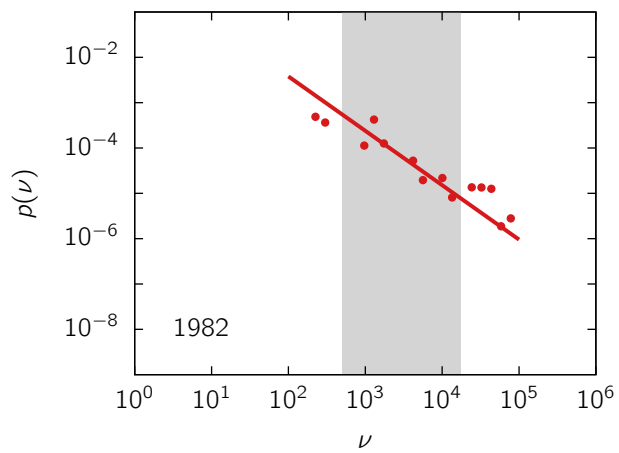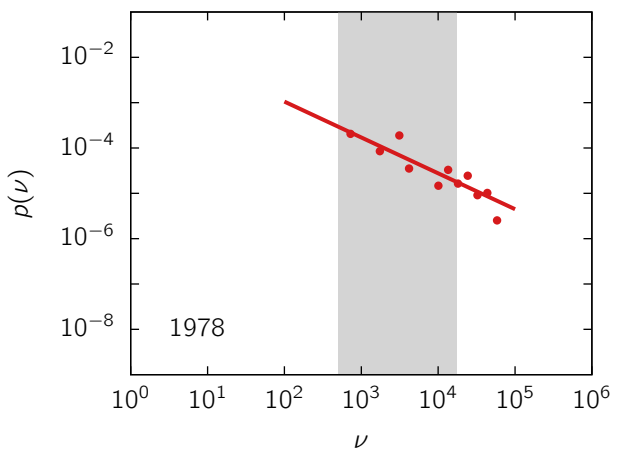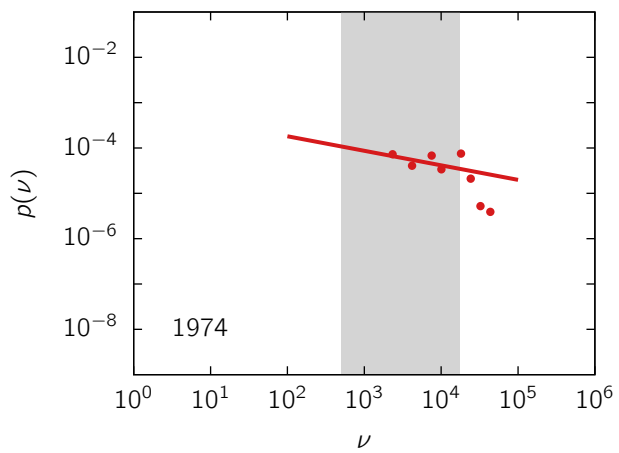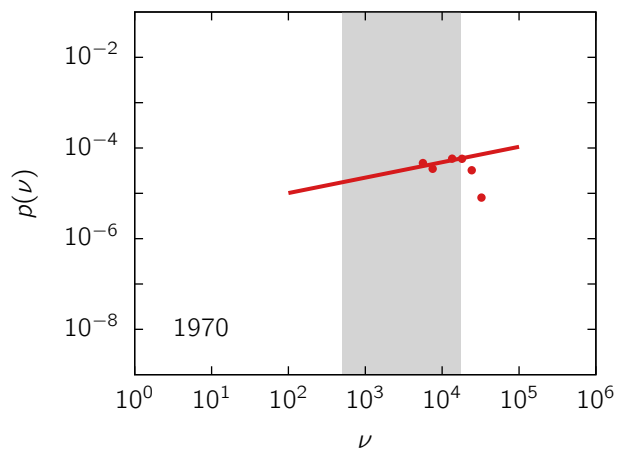

Supplement: S20 Fig — (PDF) [file pone.0137732.s026.pdf]

GO  
• Federal Deputies

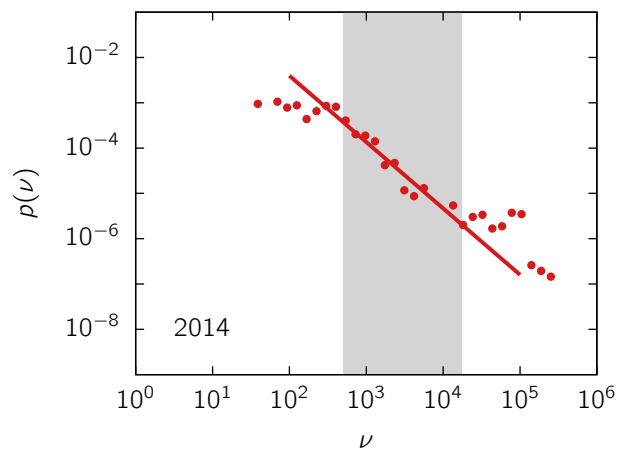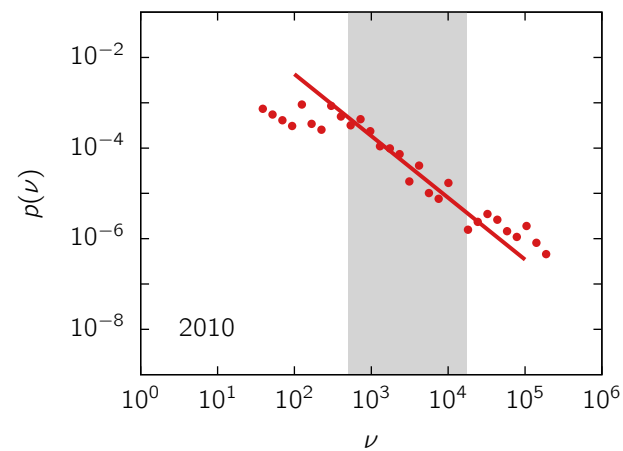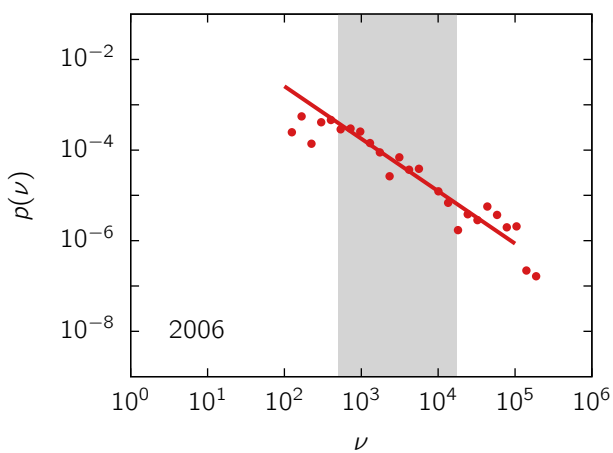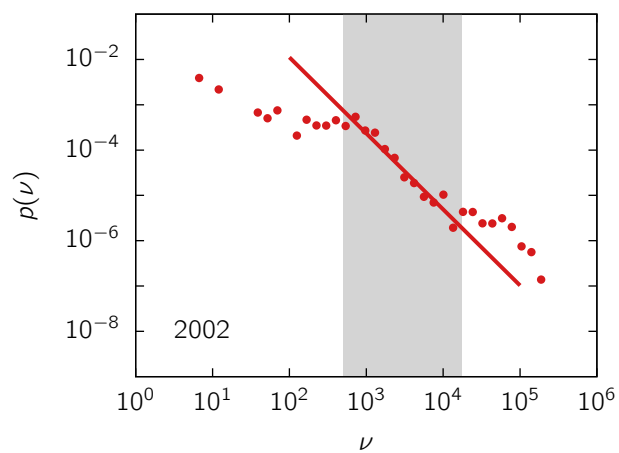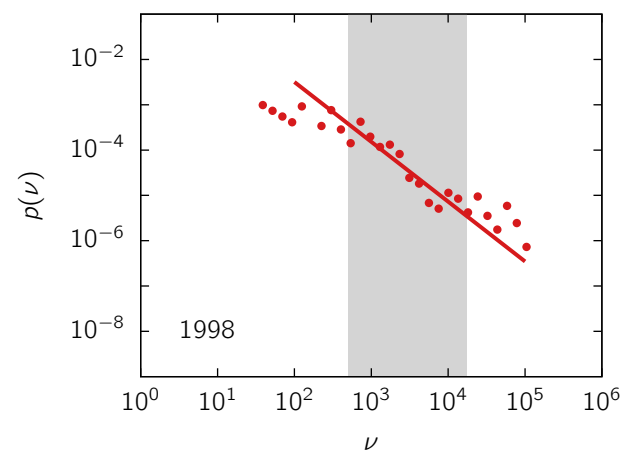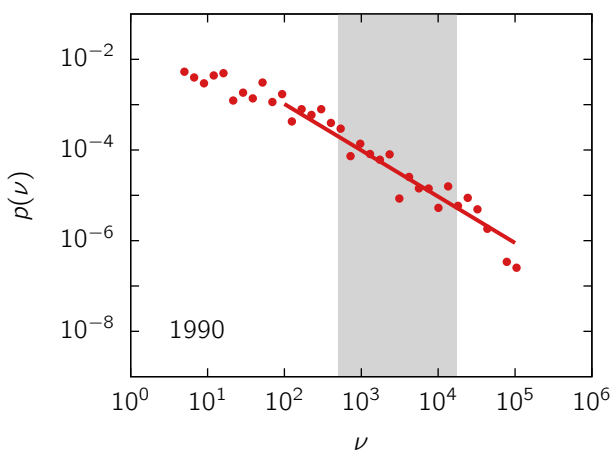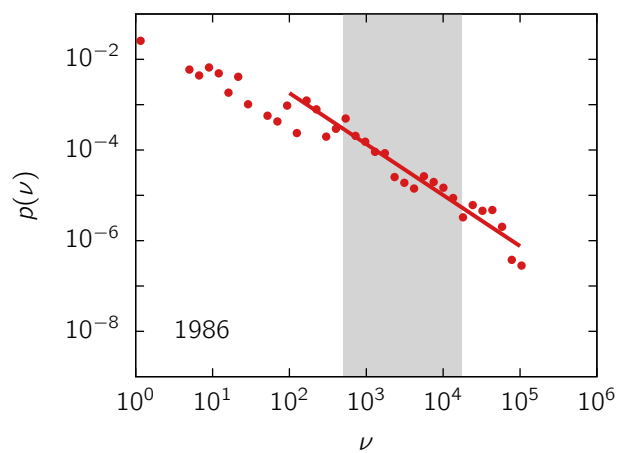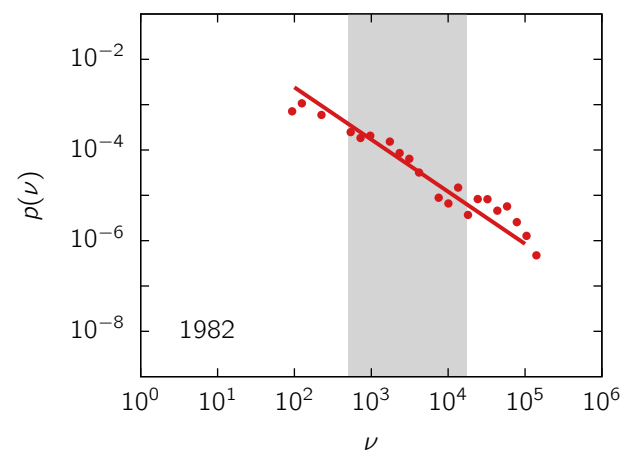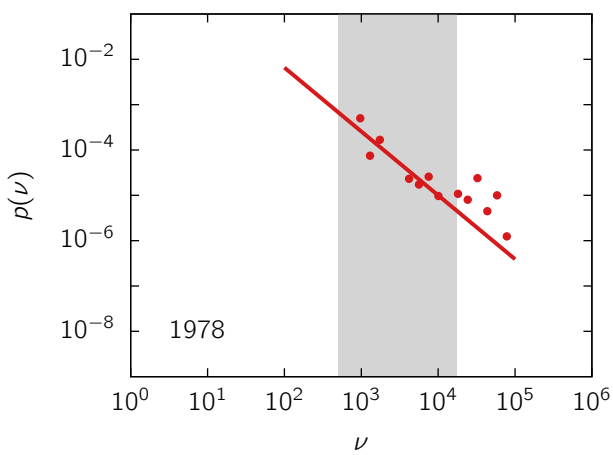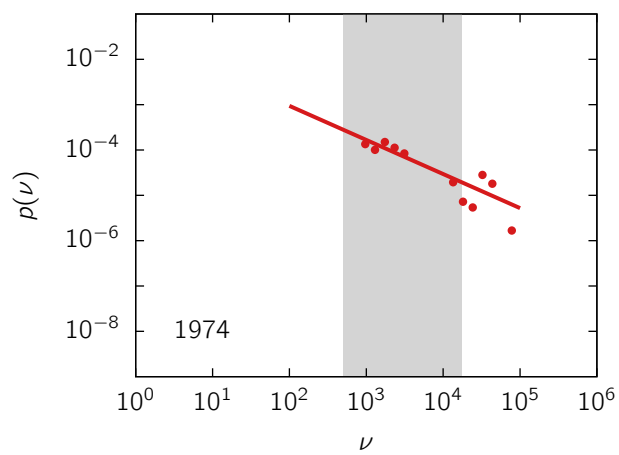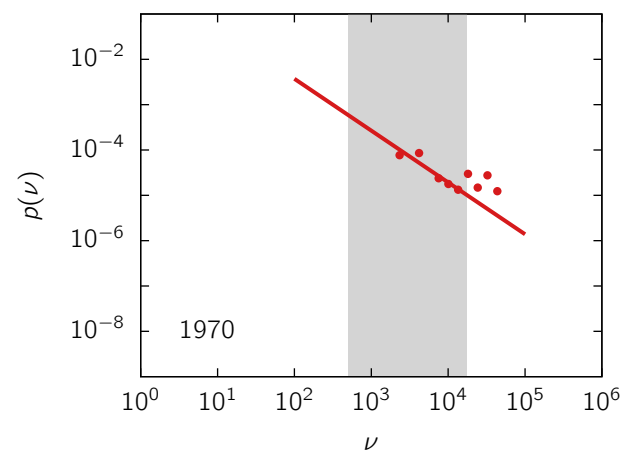

Supplement: S21 Fig — (PDF) [file pone.0137732.s027.pdf]

MA  
• Federal Deputies

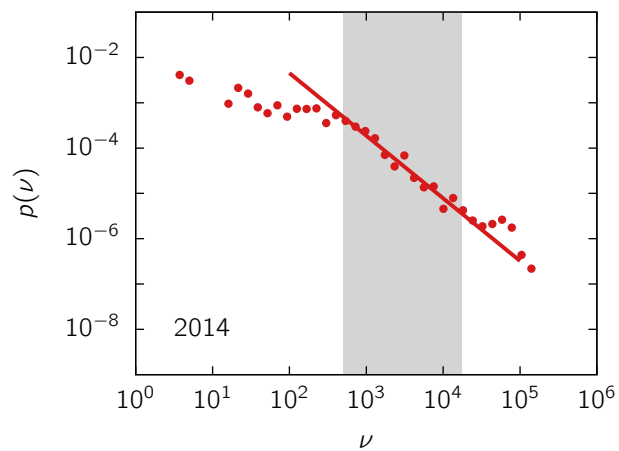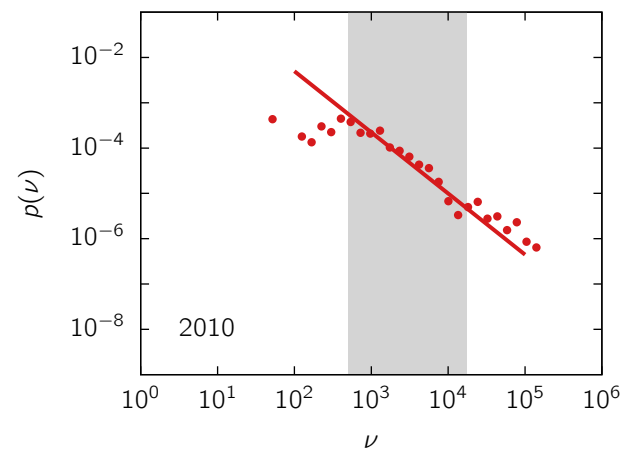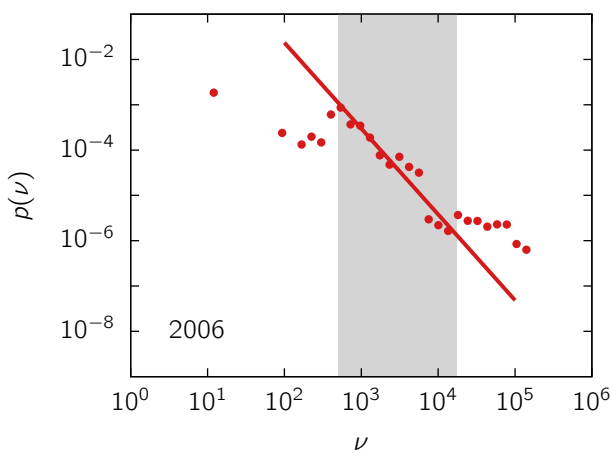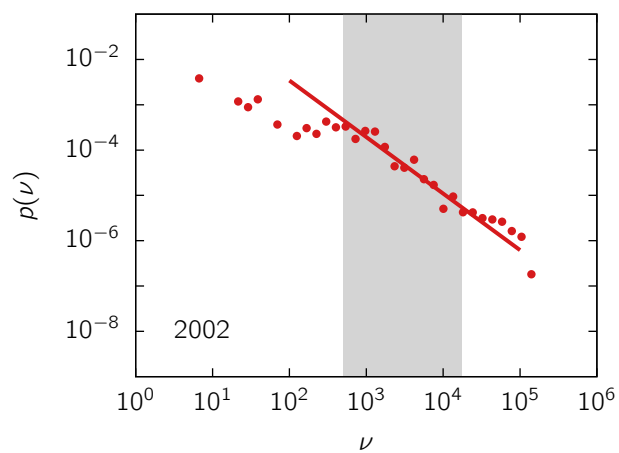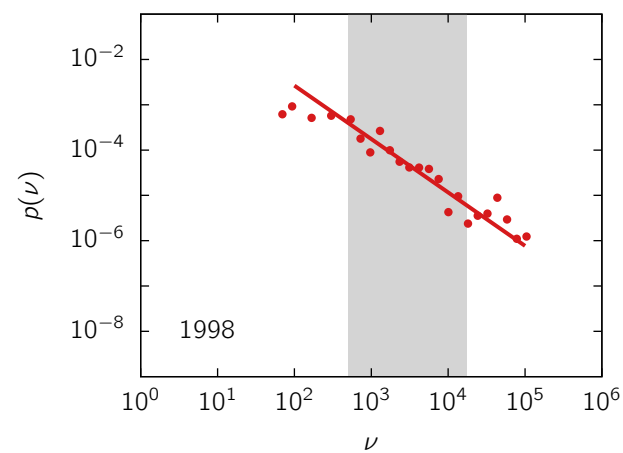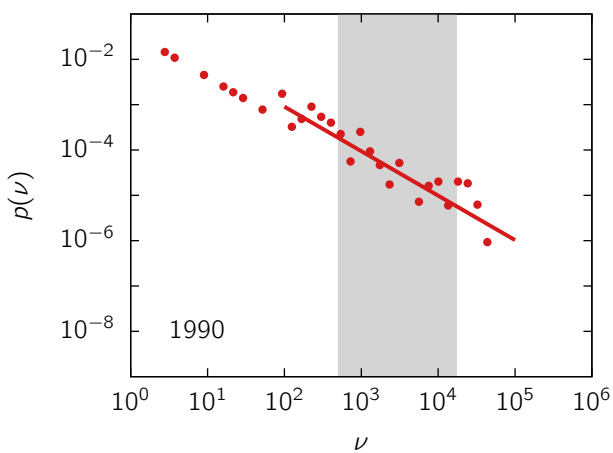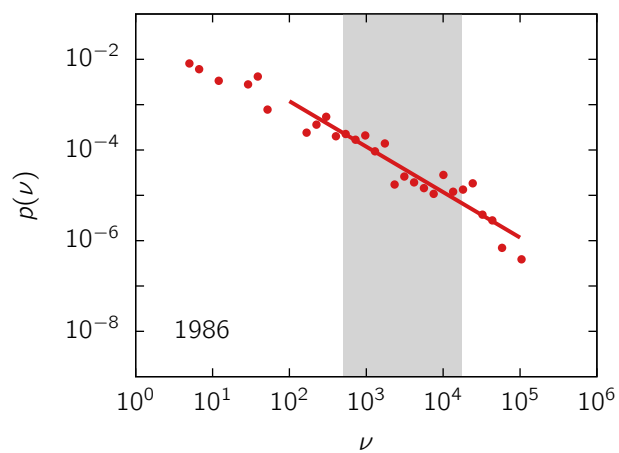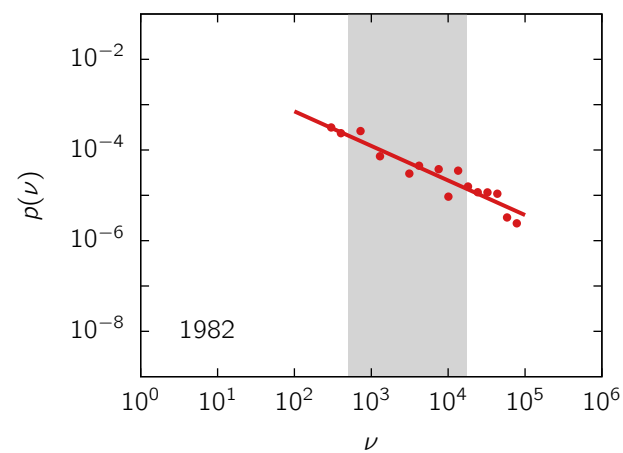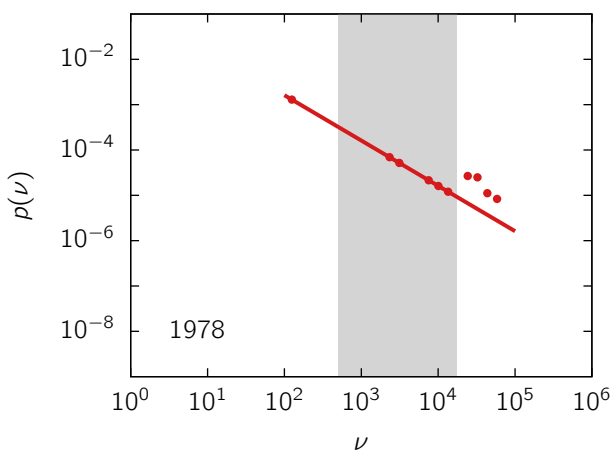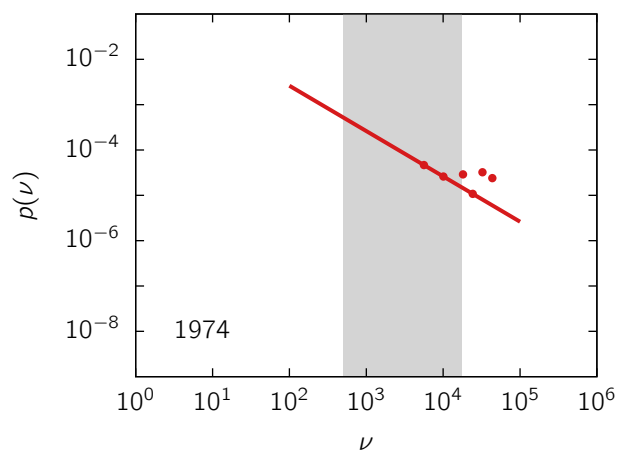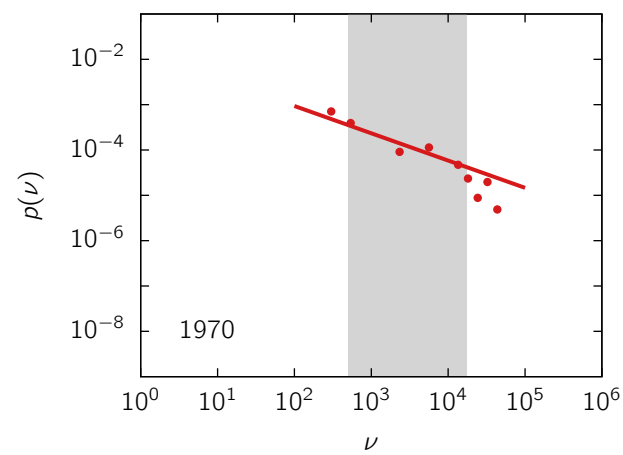

Supplement: S22 Fig — (PDF) [file pone.0137732.s028.pdf]

MT  
• Federal Deputies

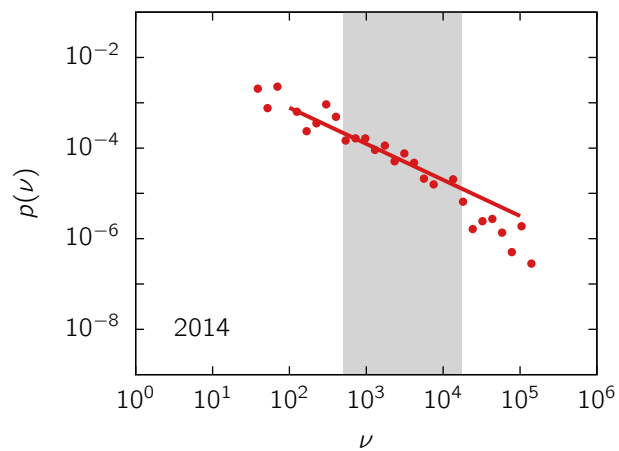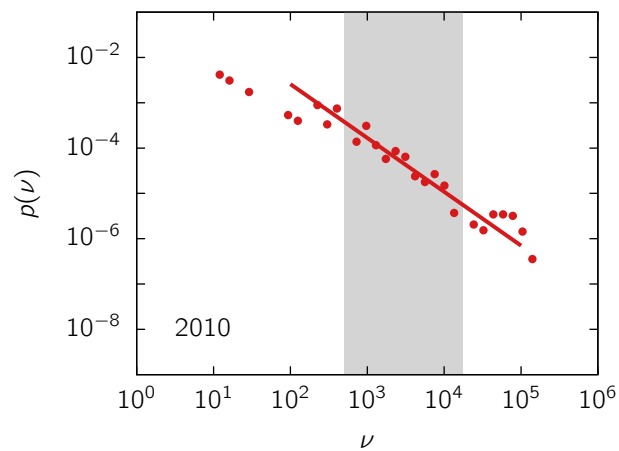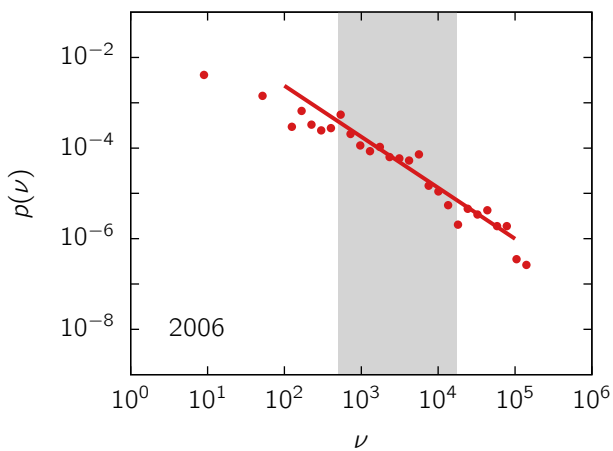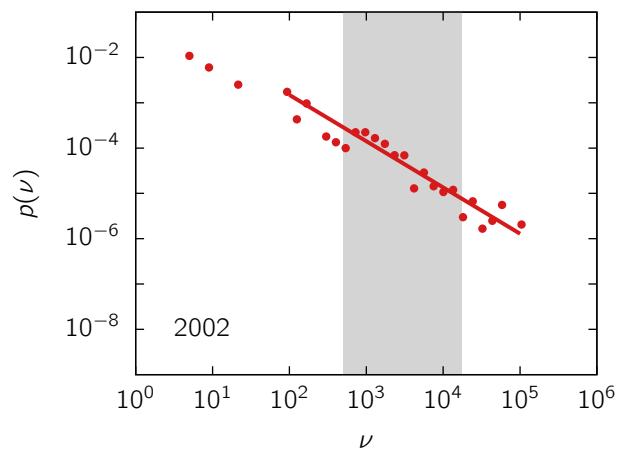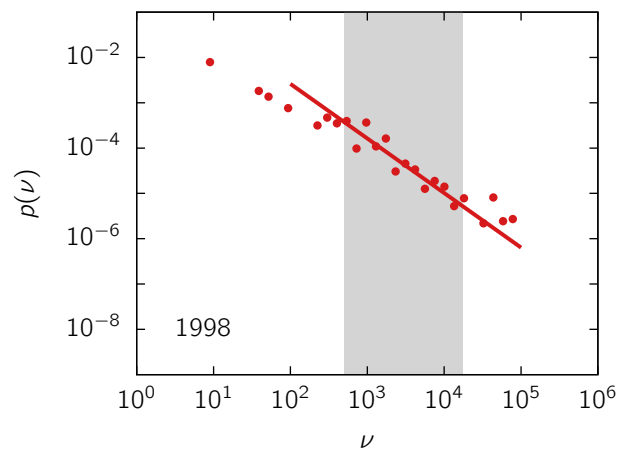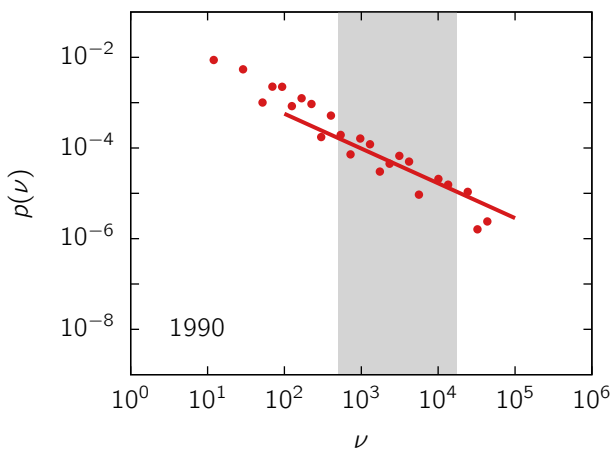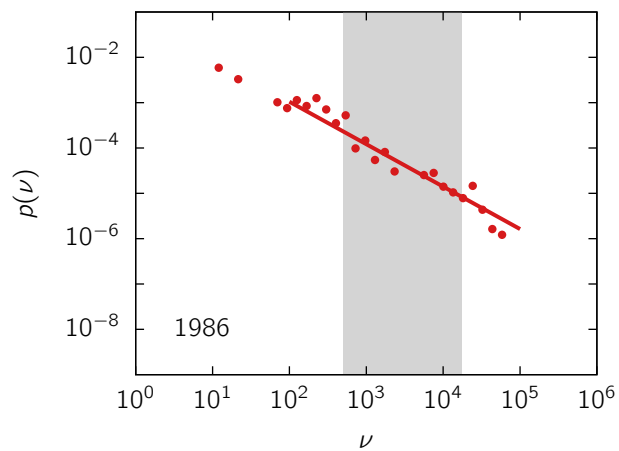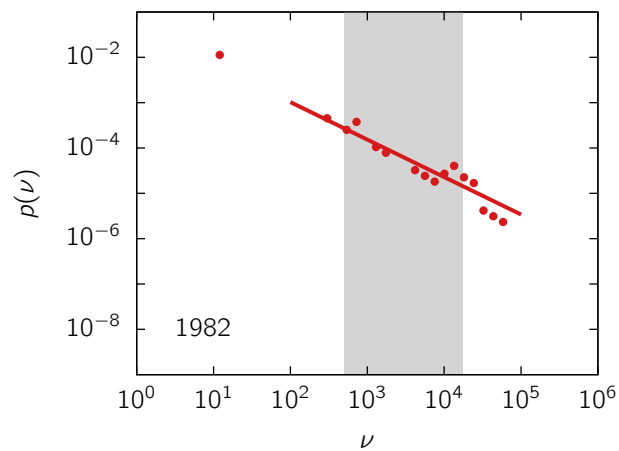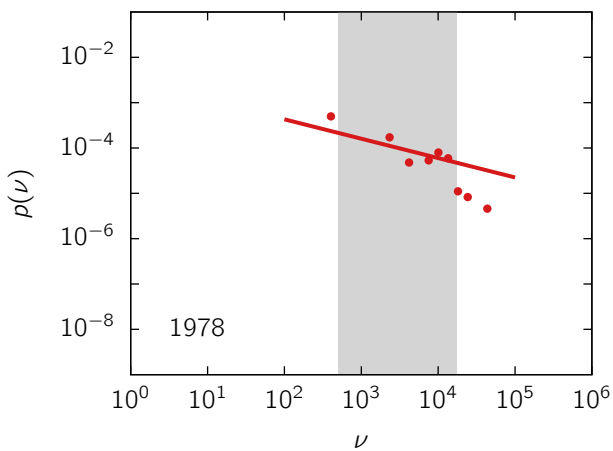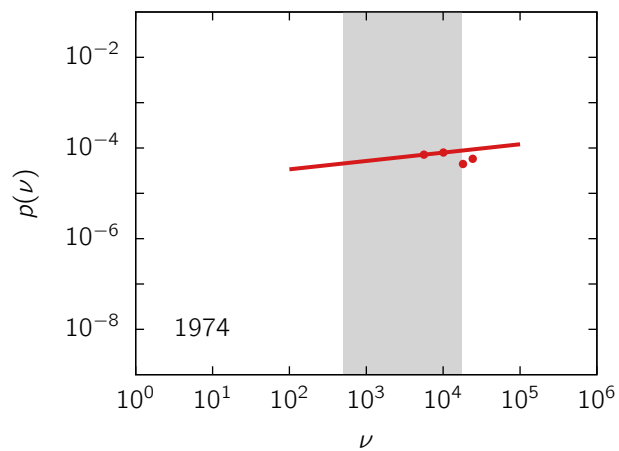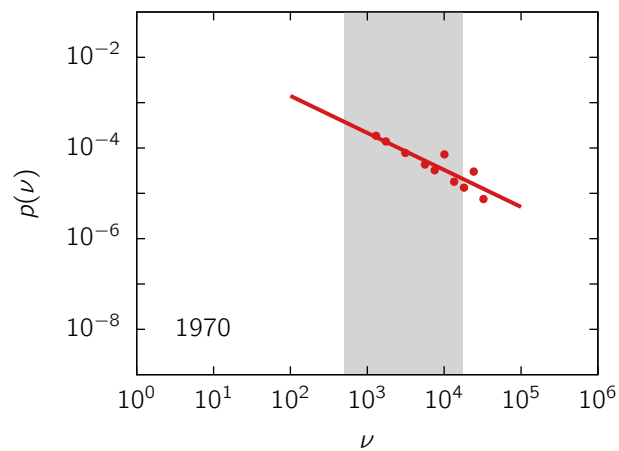

Supplement: S23 Fig — (PDF) [file pone.0137732.s029.pdf]

●

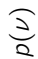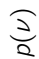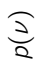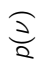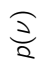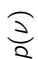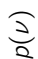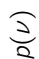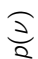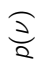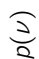

Supplement: S24 Fig — (PDF) [file pone.0137732.s030.pdf]

PA  
• Federal Deputies

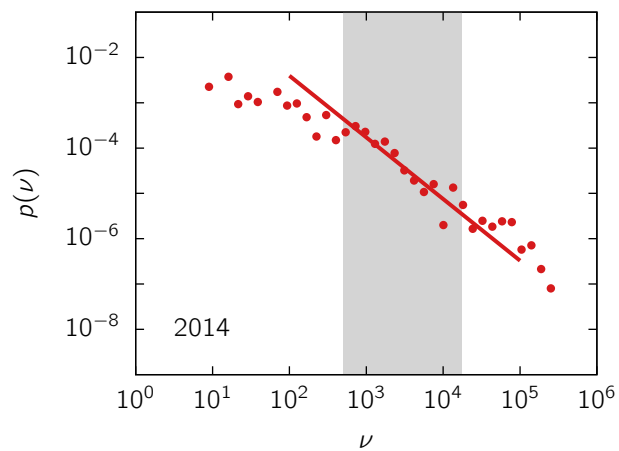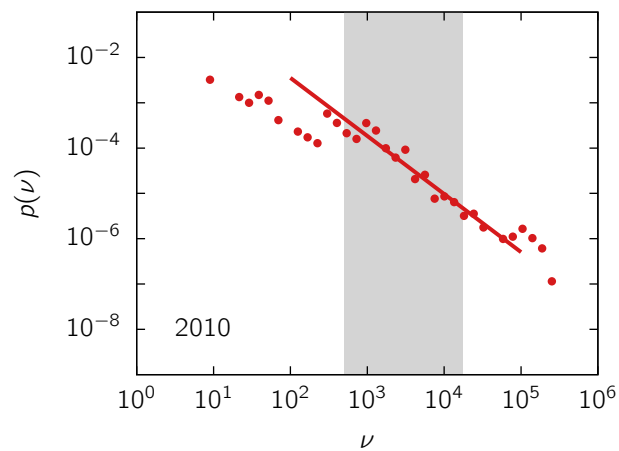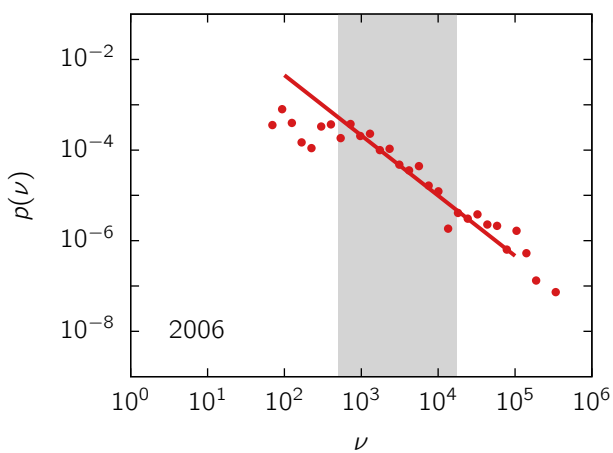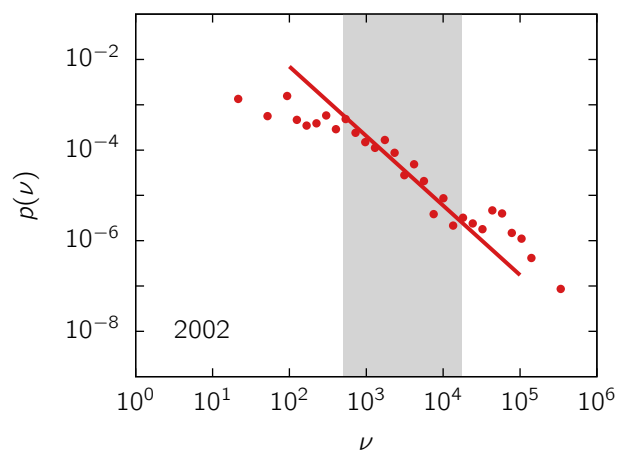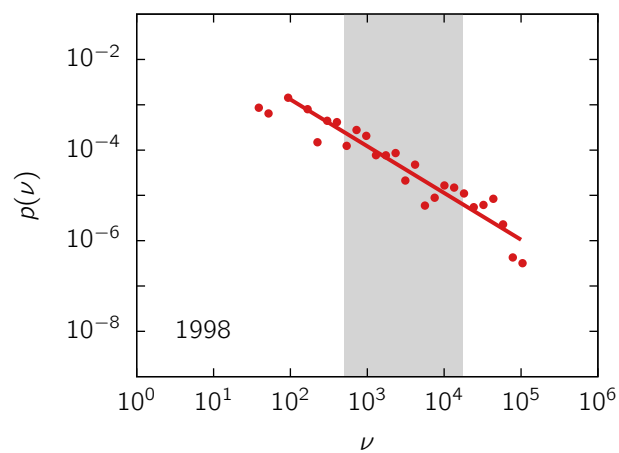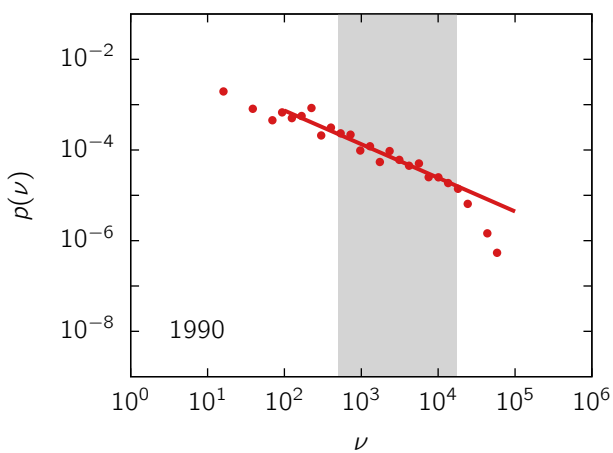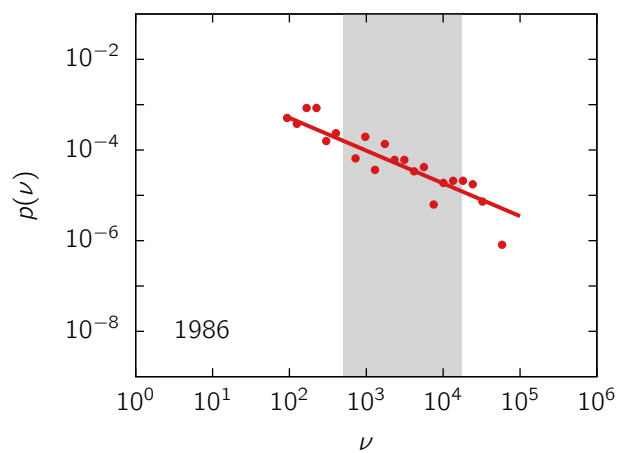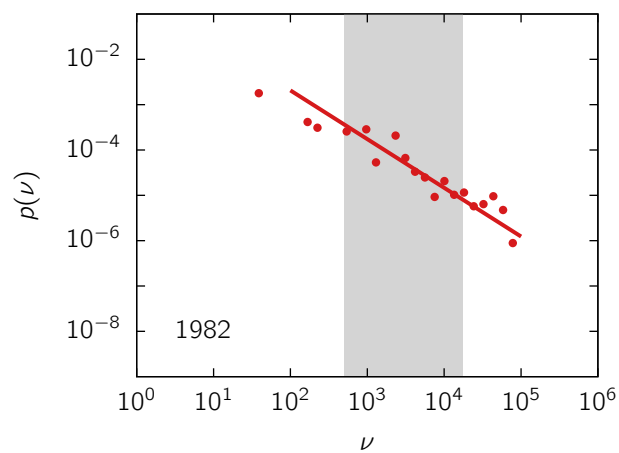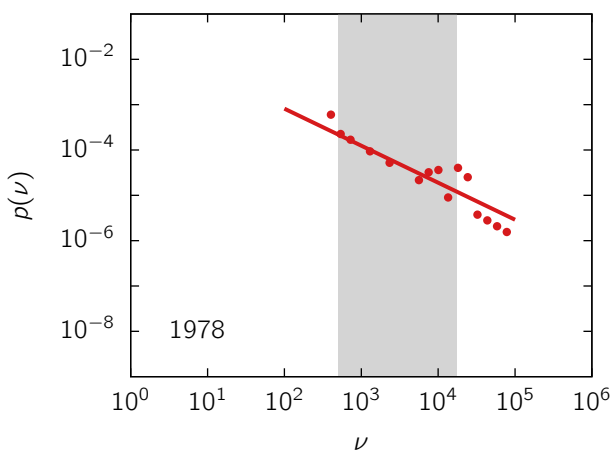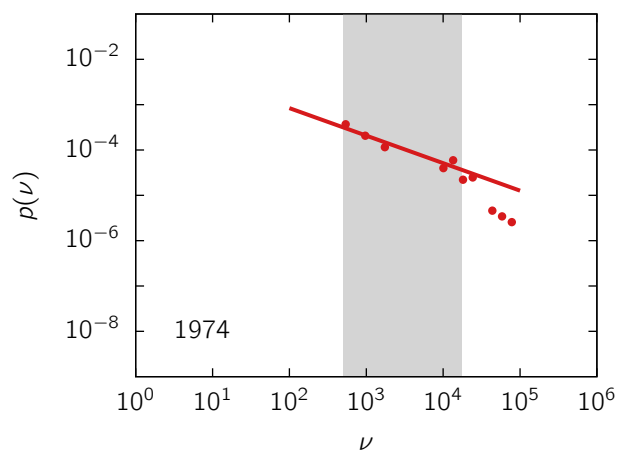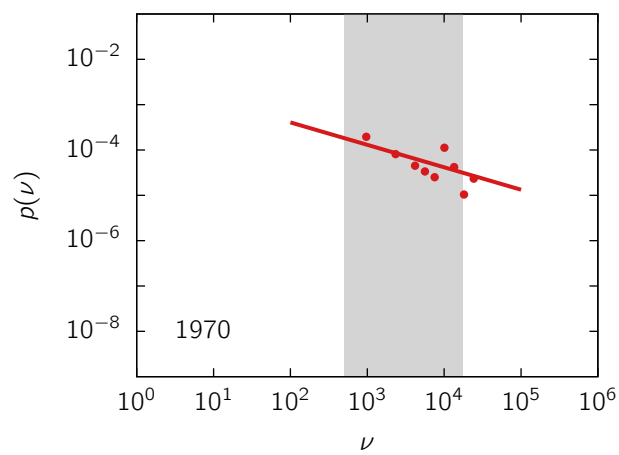

Supplement: S25 Fig — (PDF) [file pone.0137732.s031.pdf]

PB  
• Federal Deputies

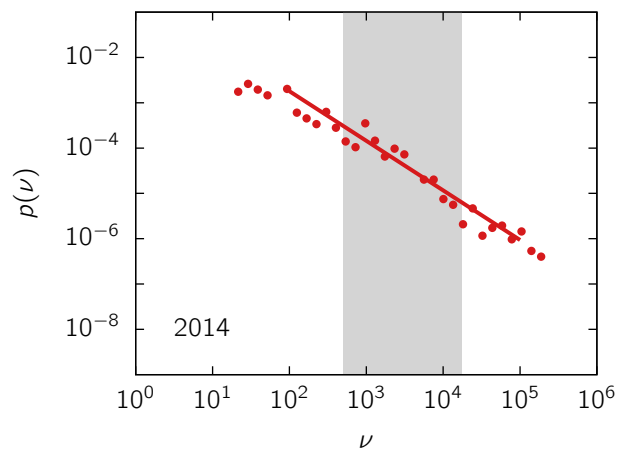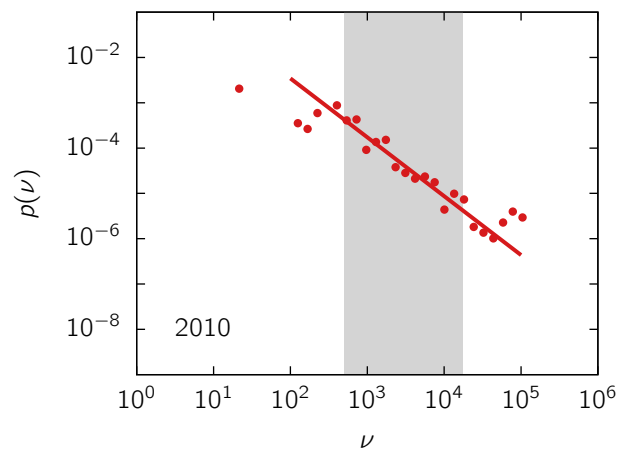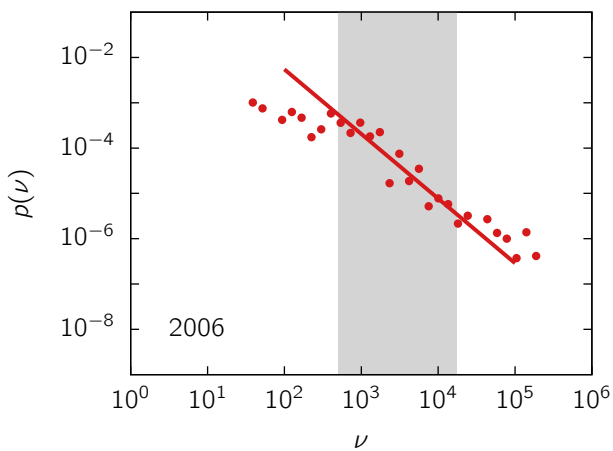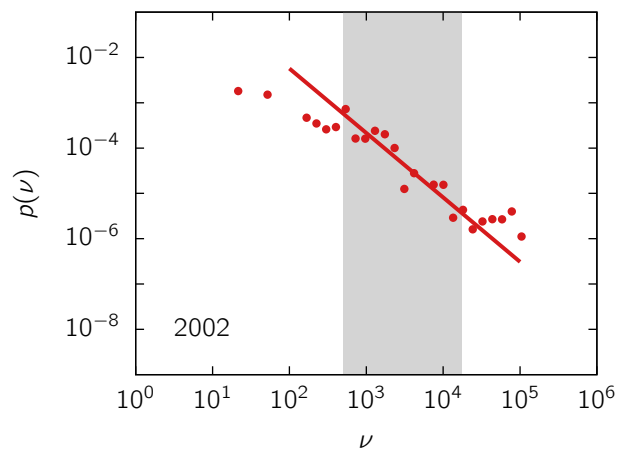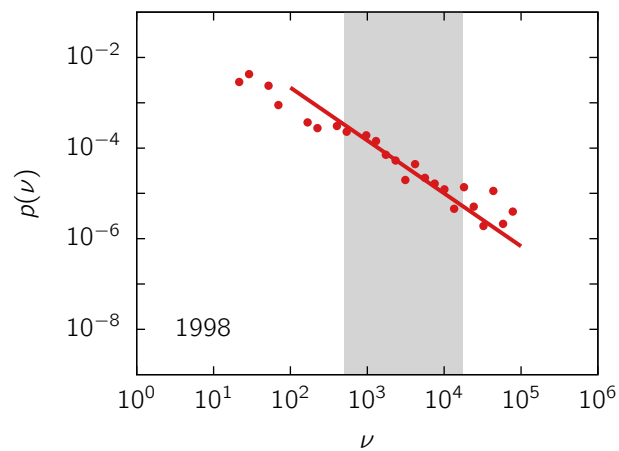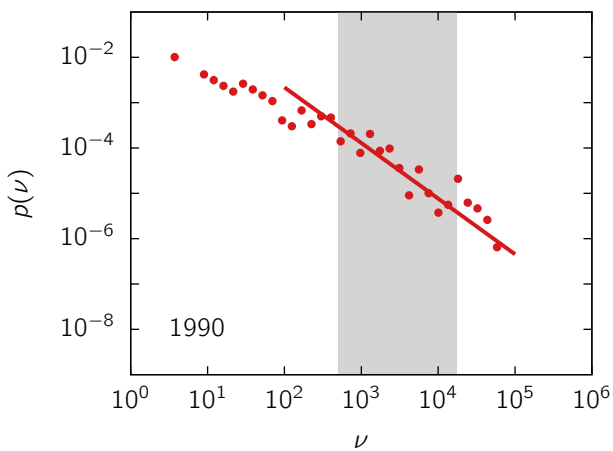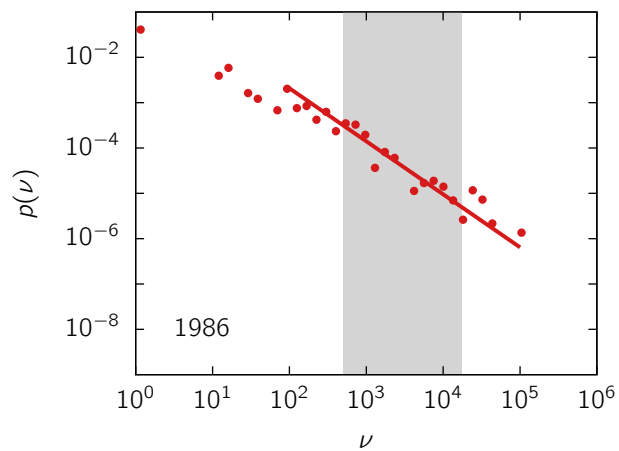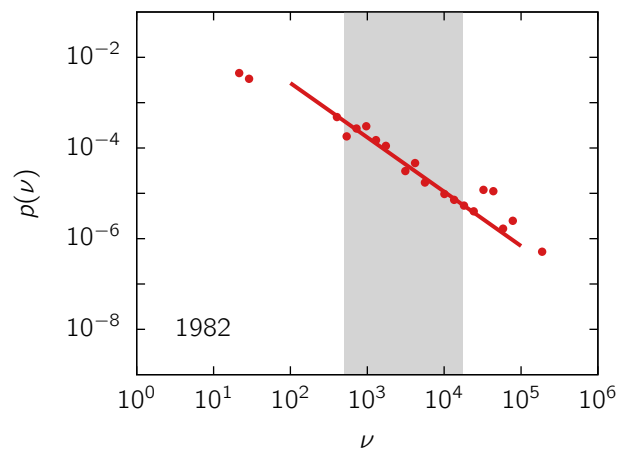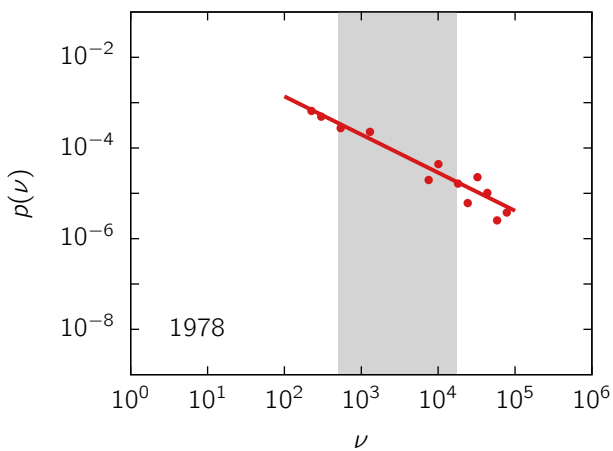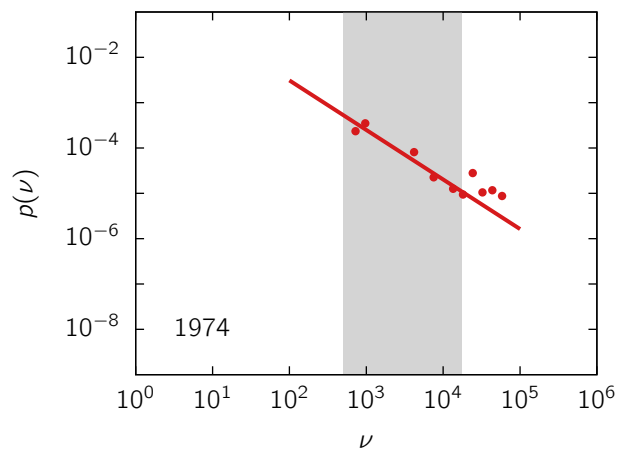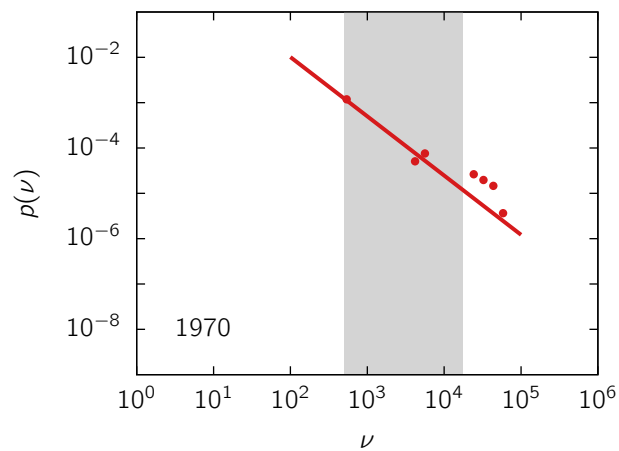

Supplement: S26 Fig — (PDF) [file pone.0137732.s032.pdf]

PR  
• Federal Deputies

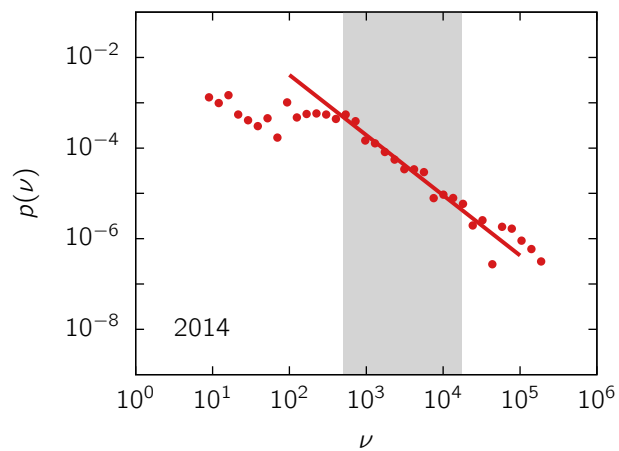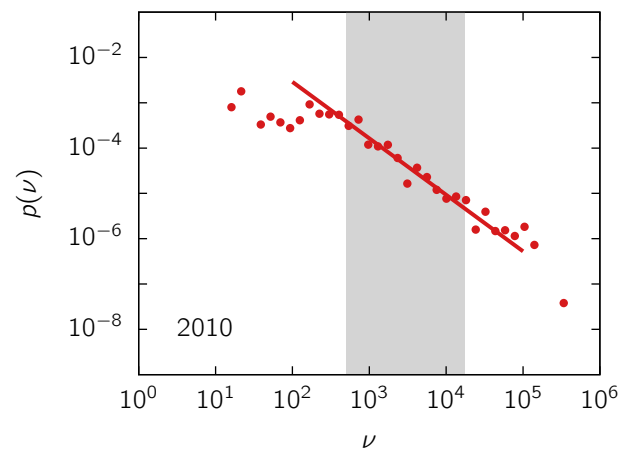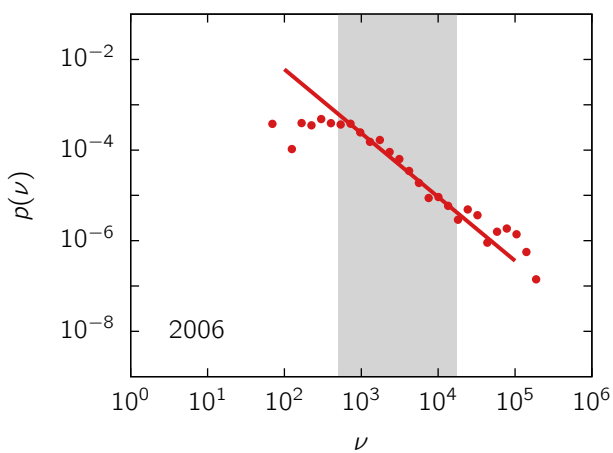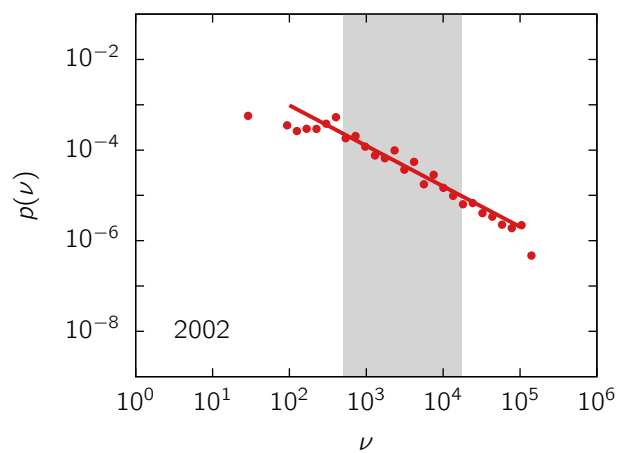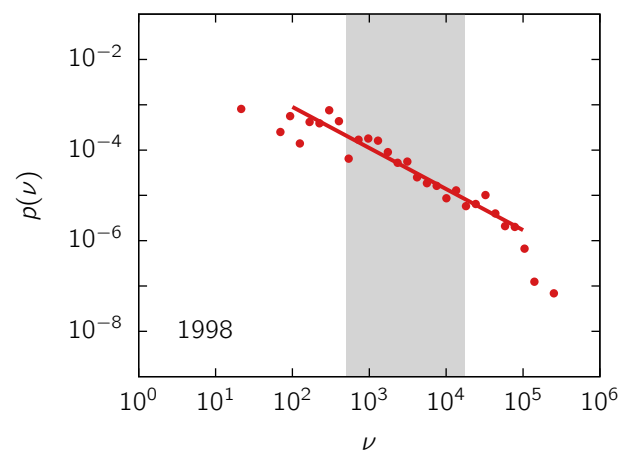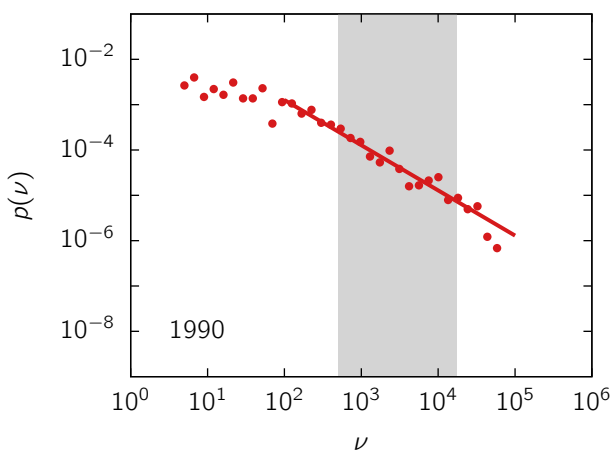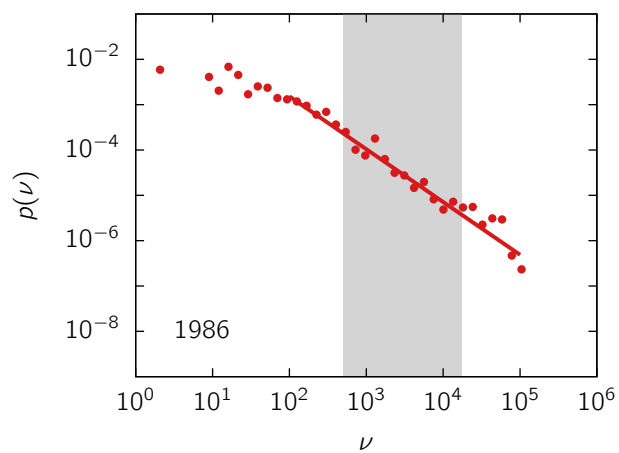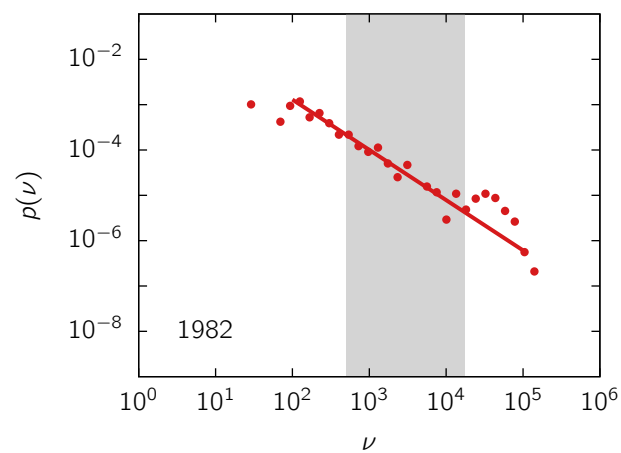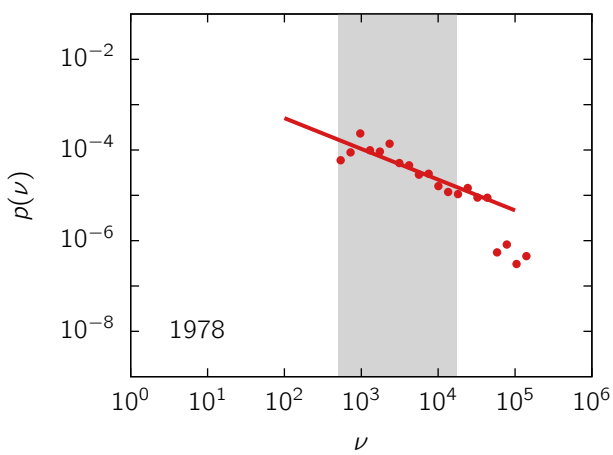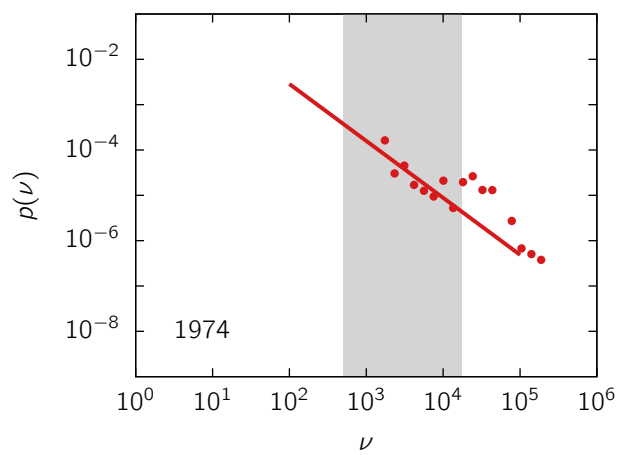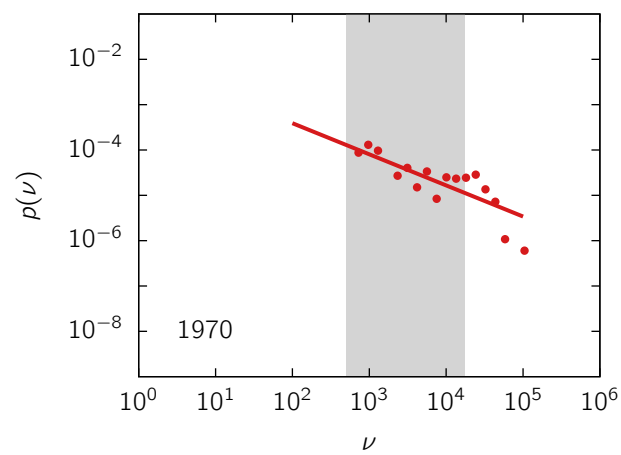

Supplement: S27 Fig — (PDF) [file pone.0137732.s033.pdf]

PE  
• Federal Deputies

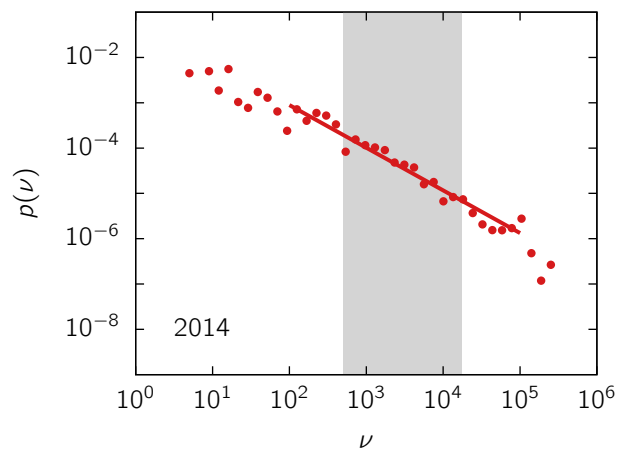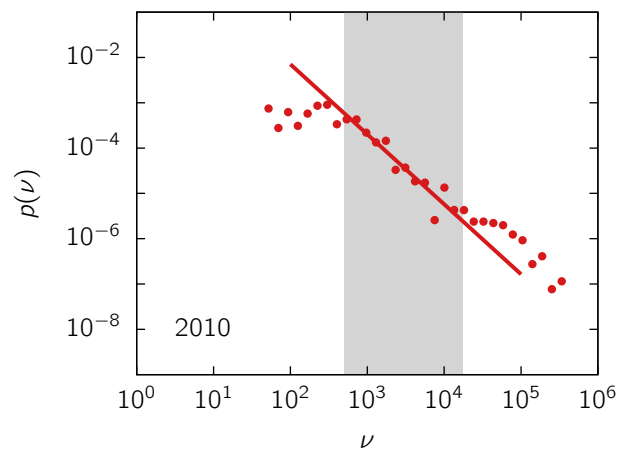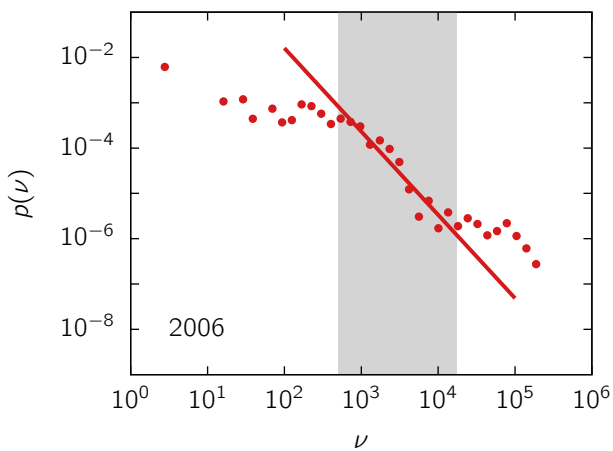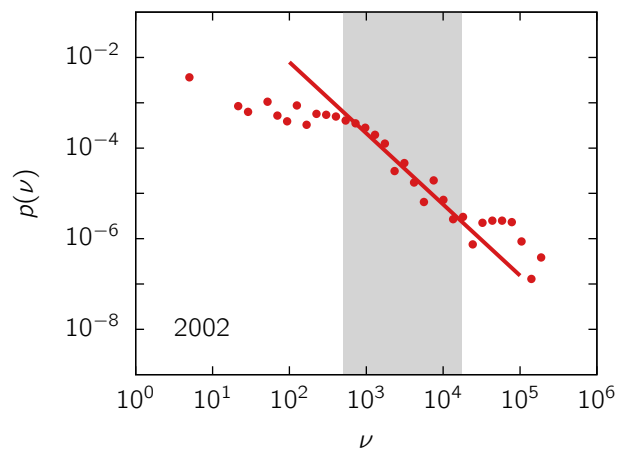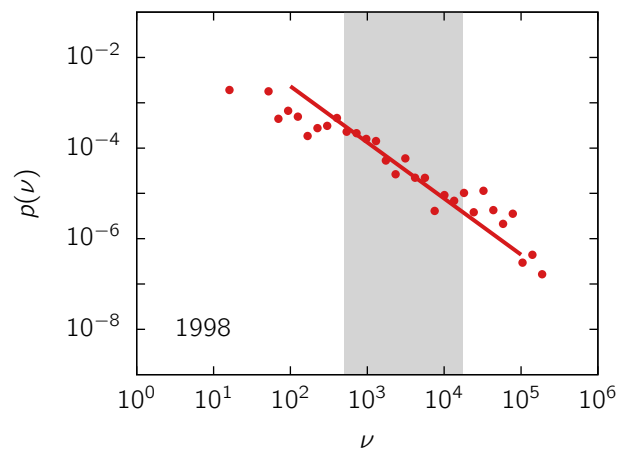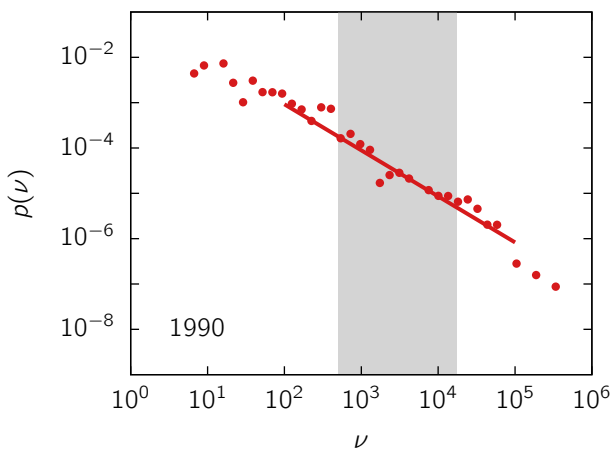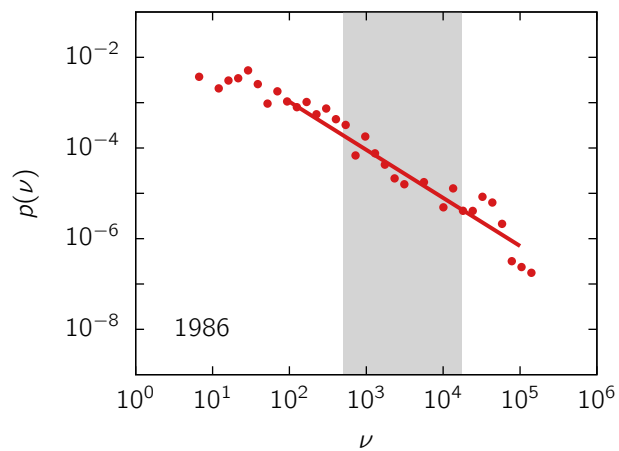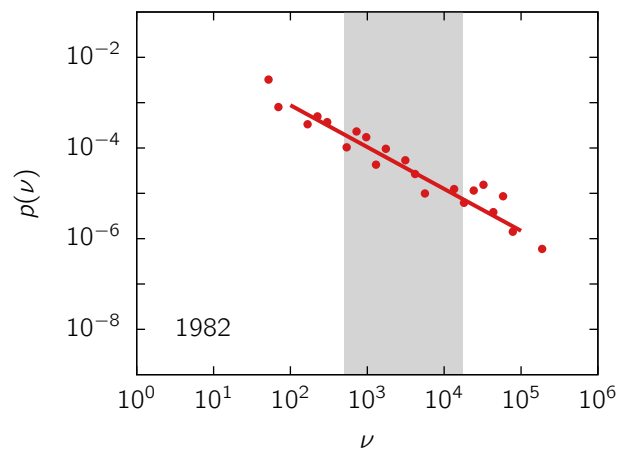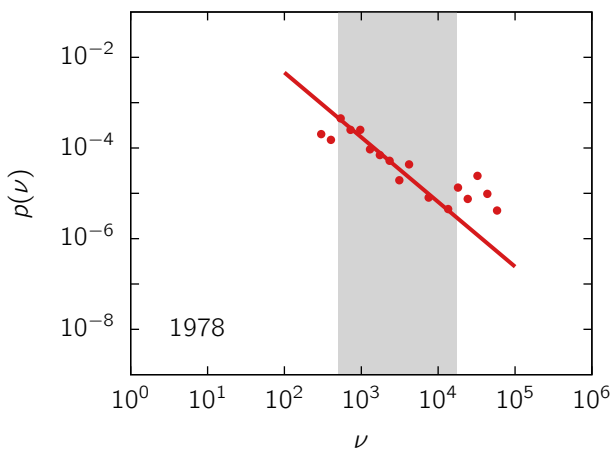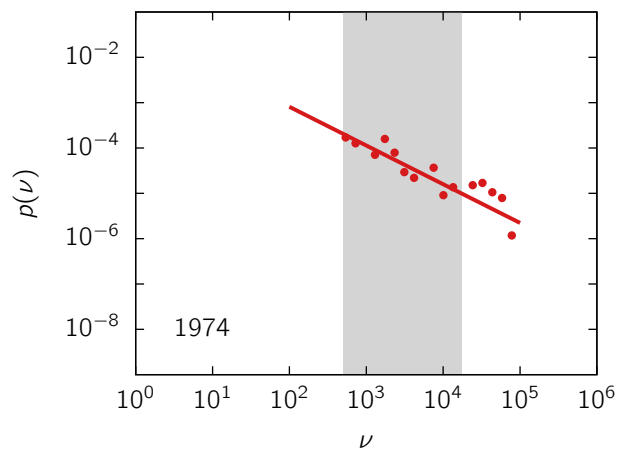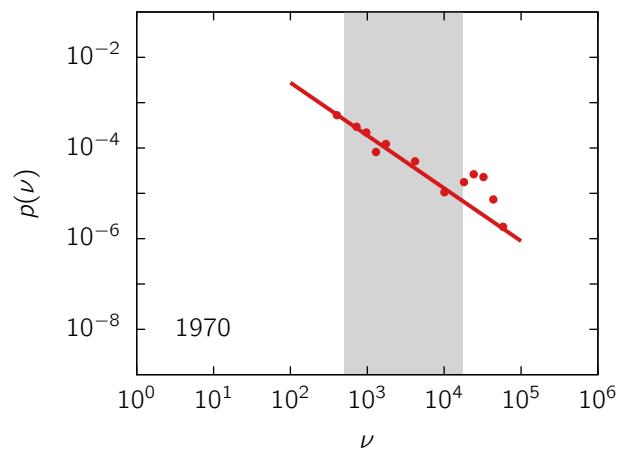

Supplement: S28 Fig — (PDF) [file pone.0137732.s034.pdf]

RN  
• Federal Deputies

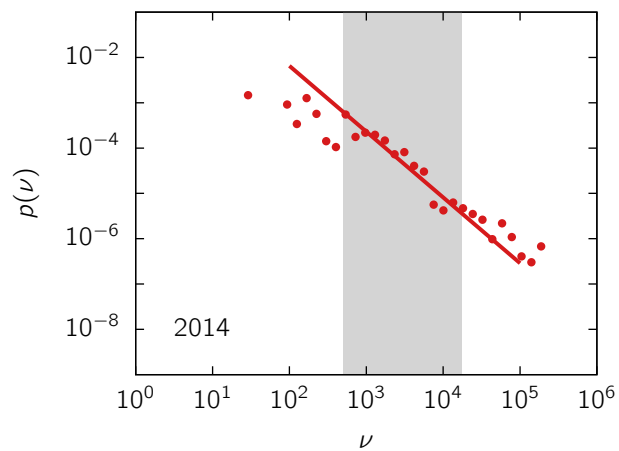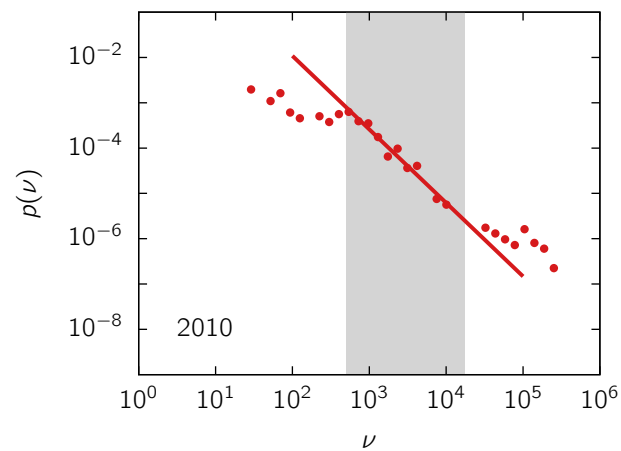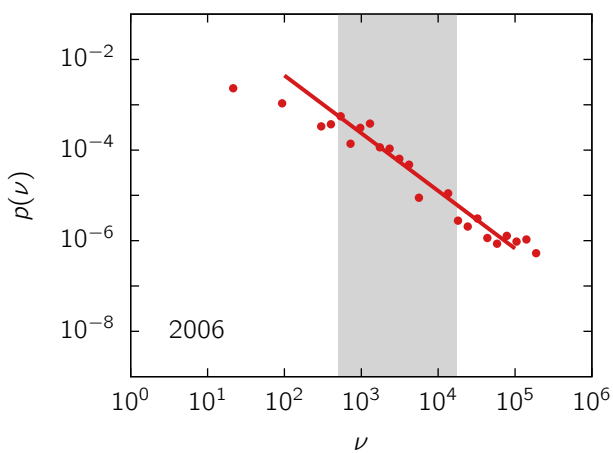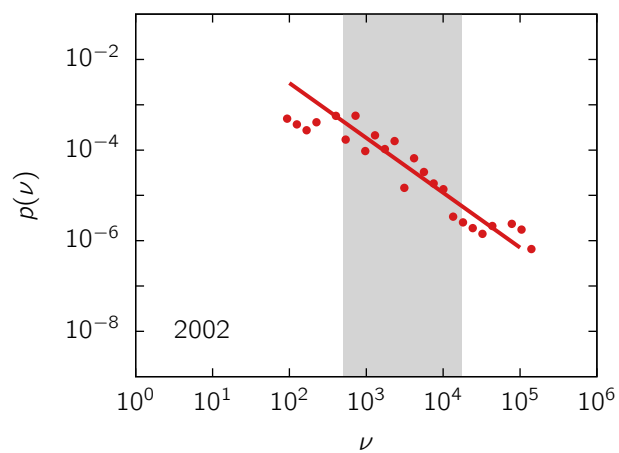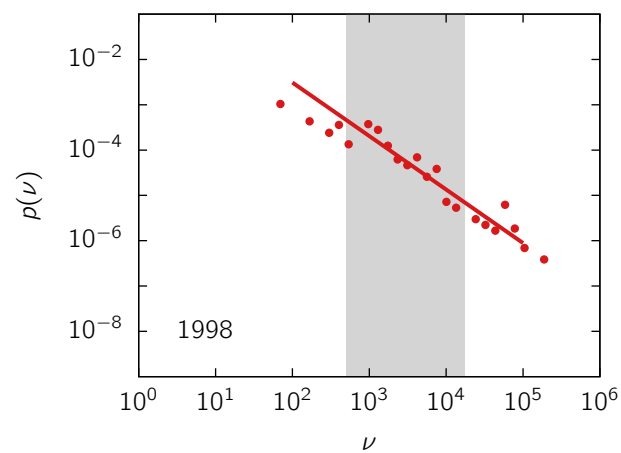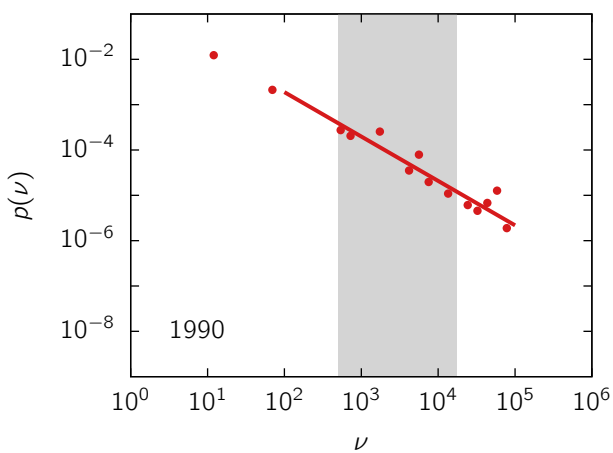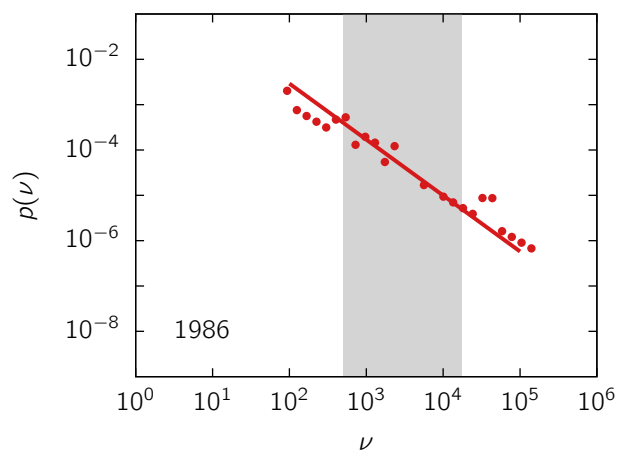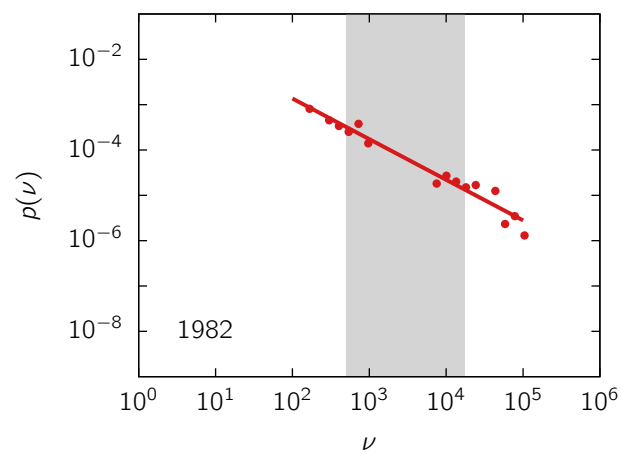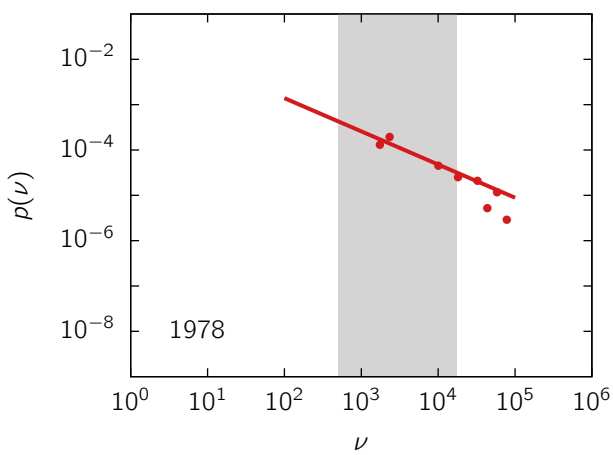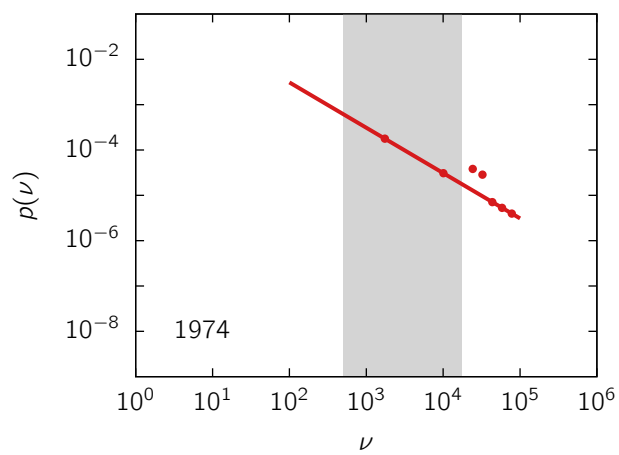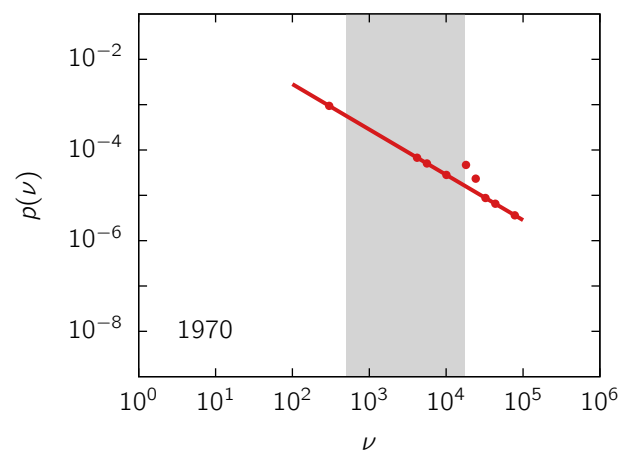

Supplement: S29 Fig — (PDF) [file pone.0137732.s035.pdf]

RS  
• Federal Deputies

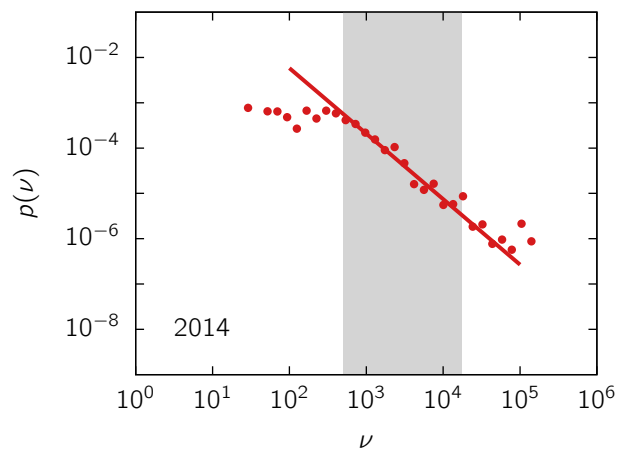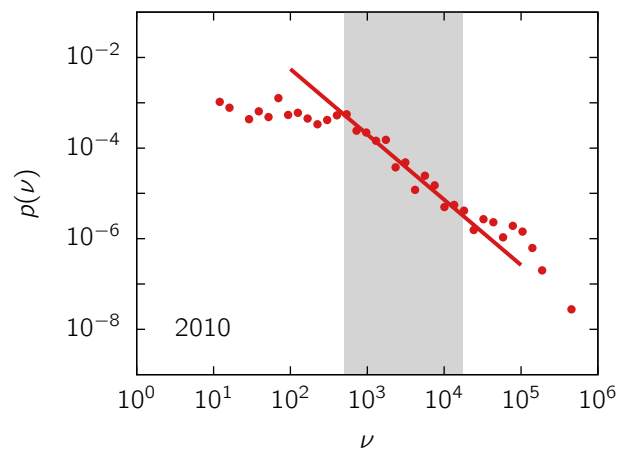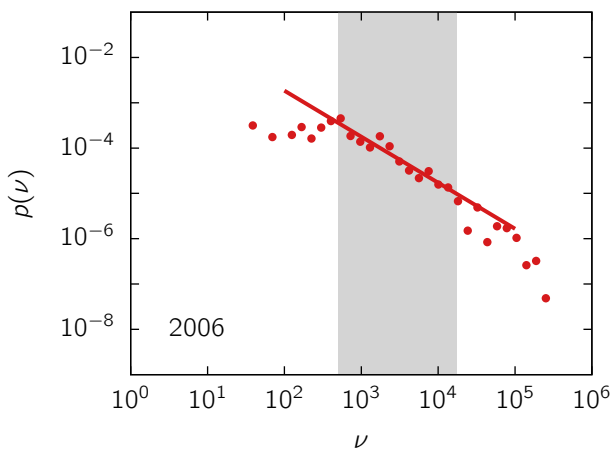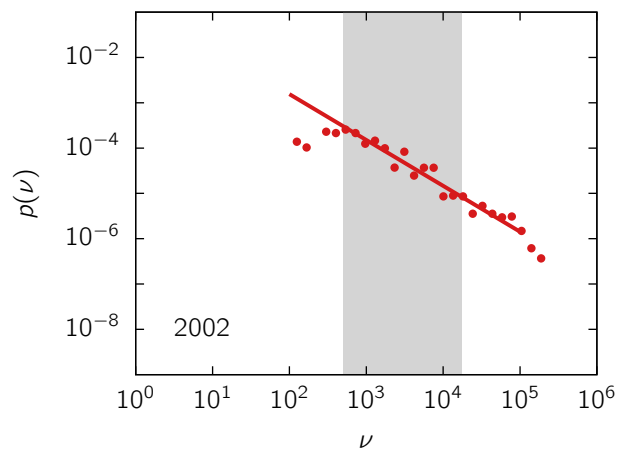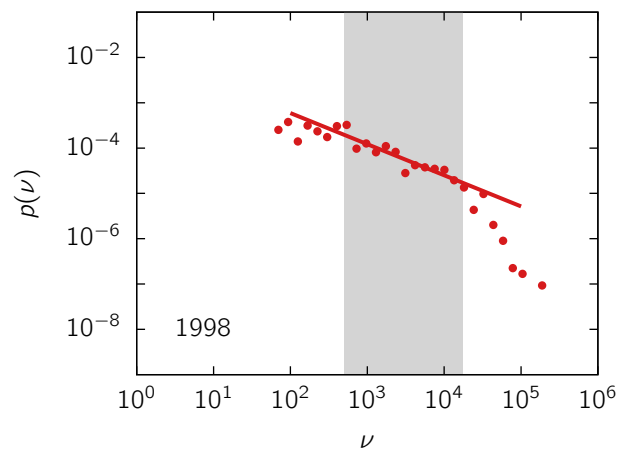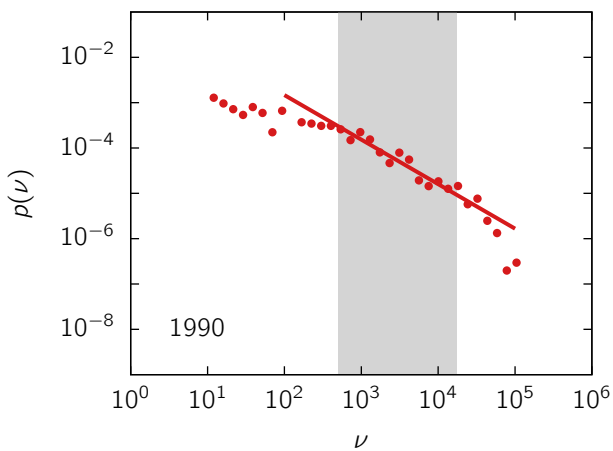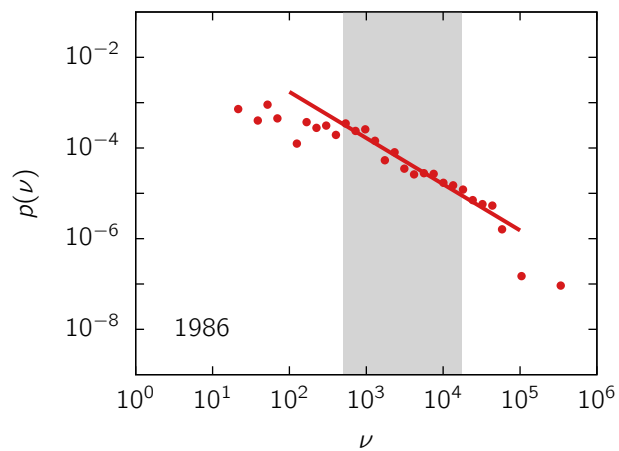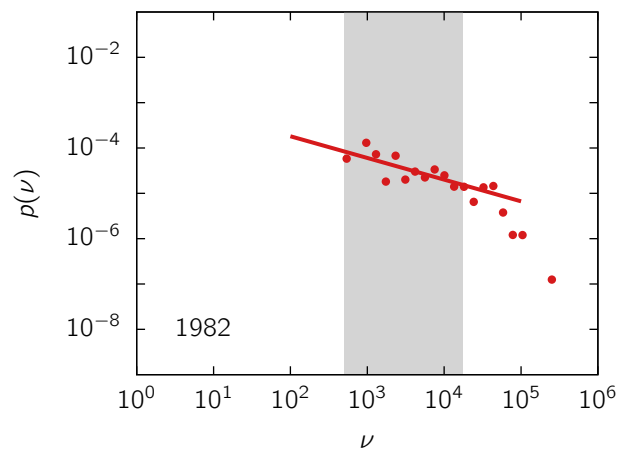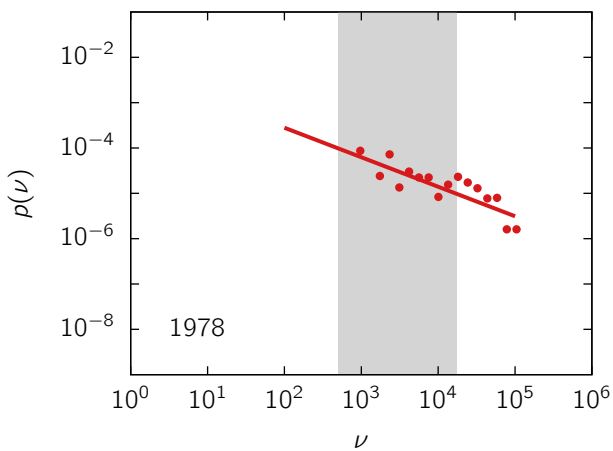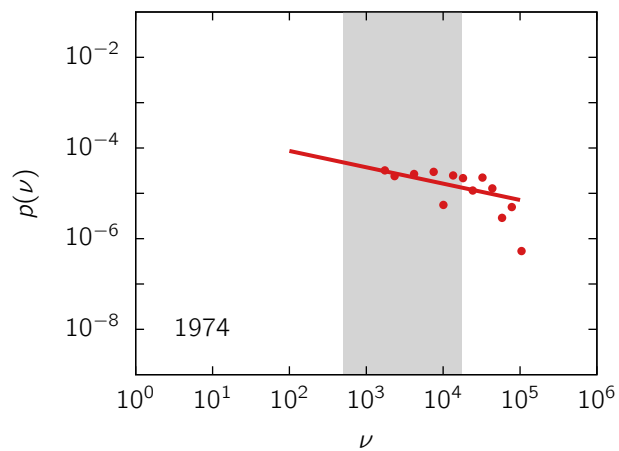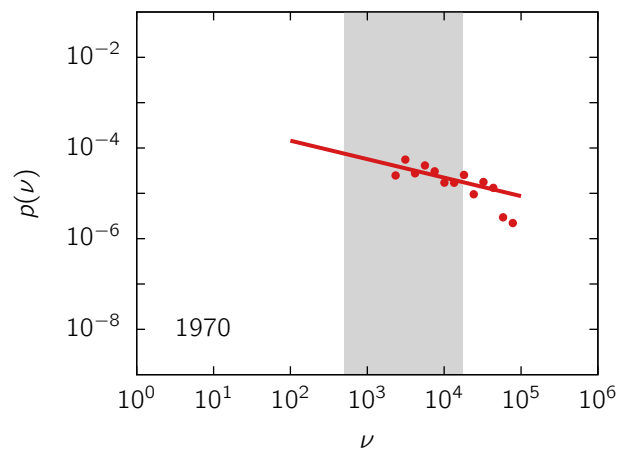

Supplement: S30 Fig — (PDF) [file pone.0137732.s036.pdf]

RO  
• Federal Deputies

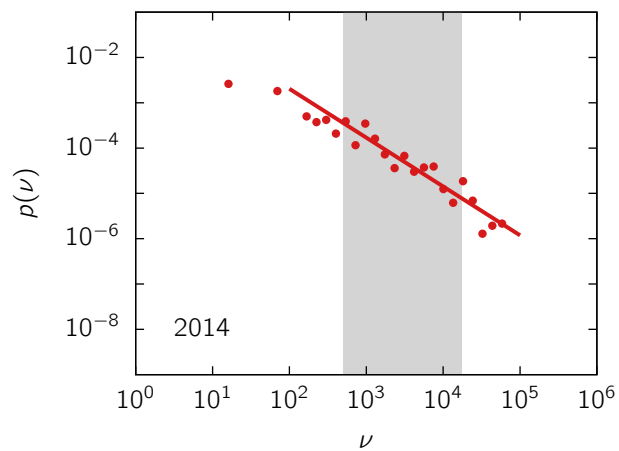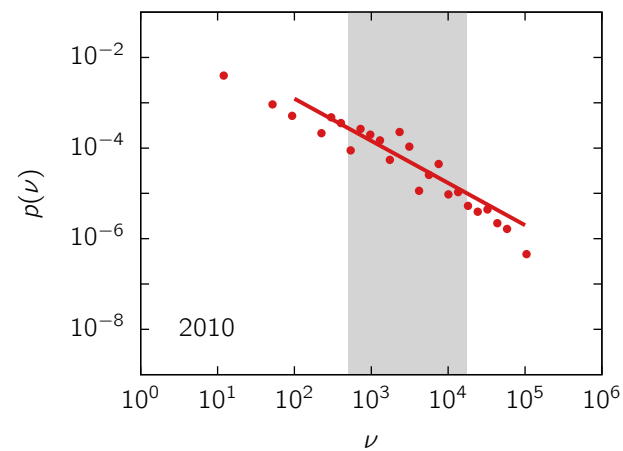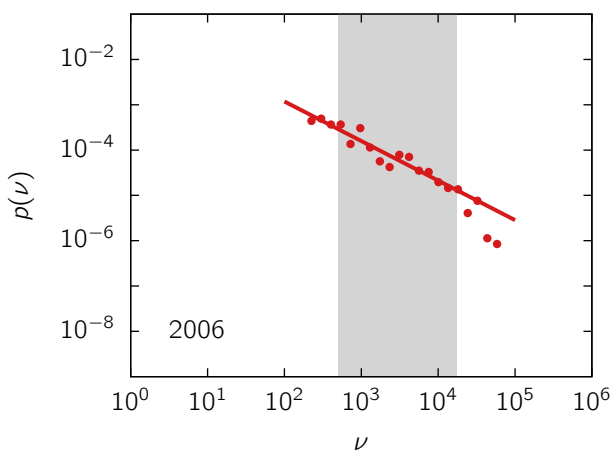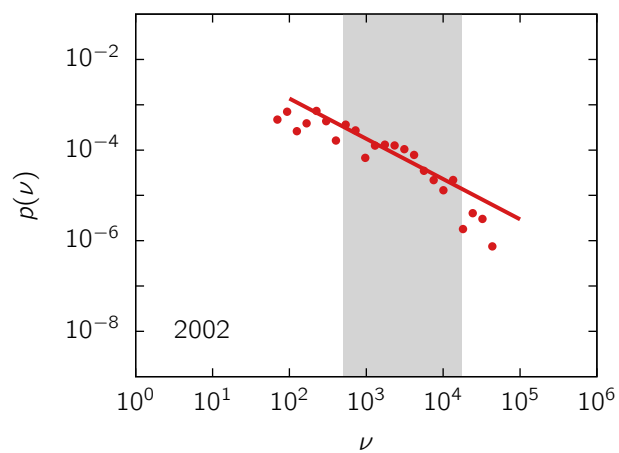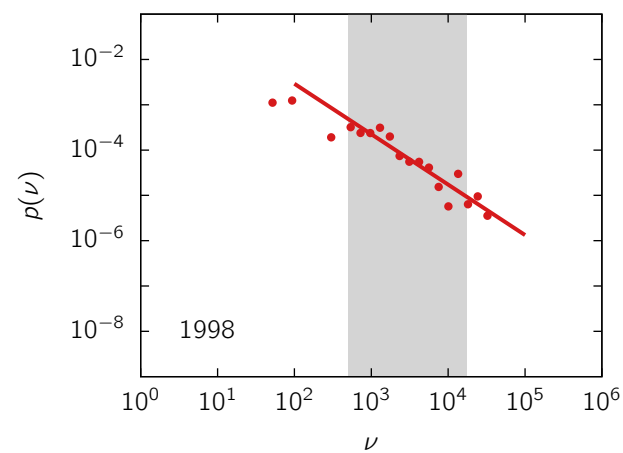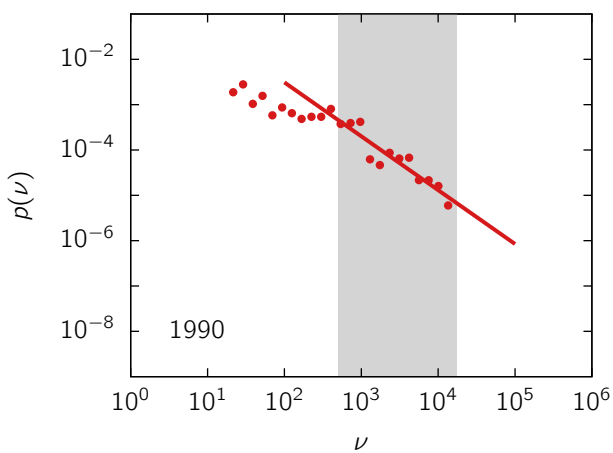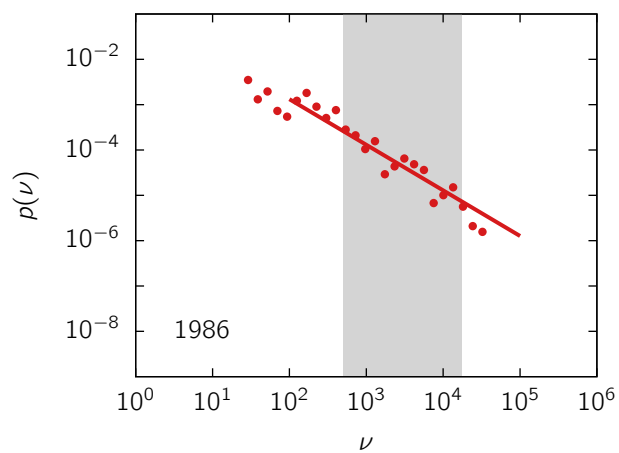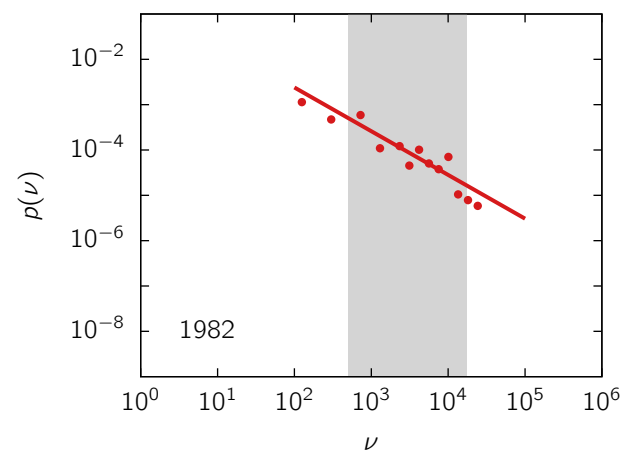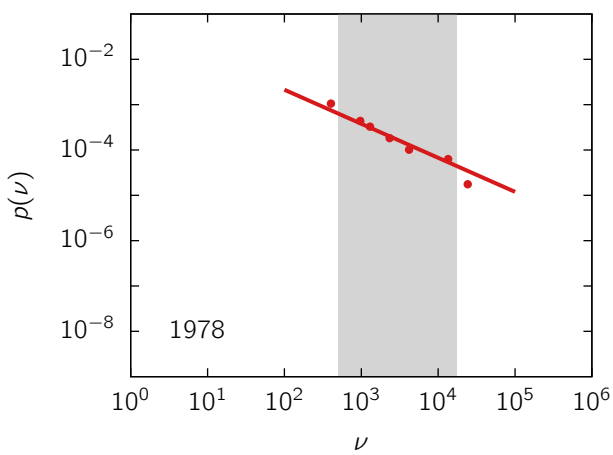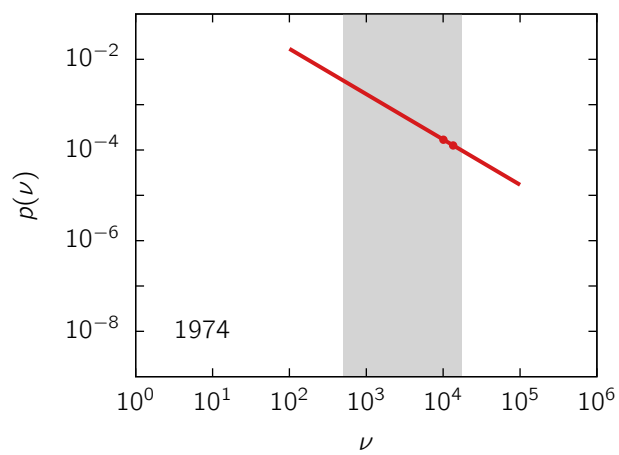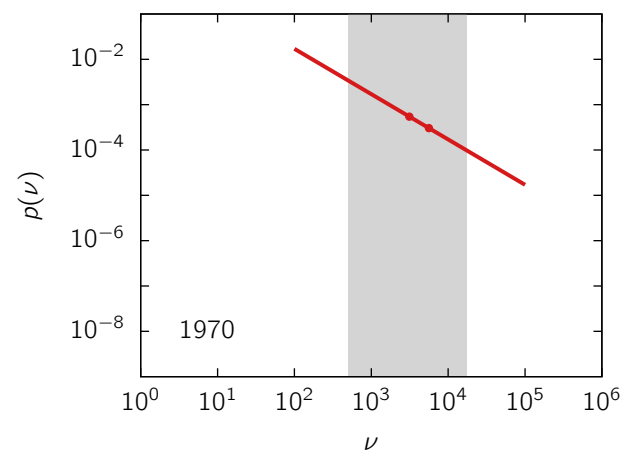

Supplement: S31 Fig — (PDF) [file pone.0137732.s037.pdf]

RR  
• Federal Deputies

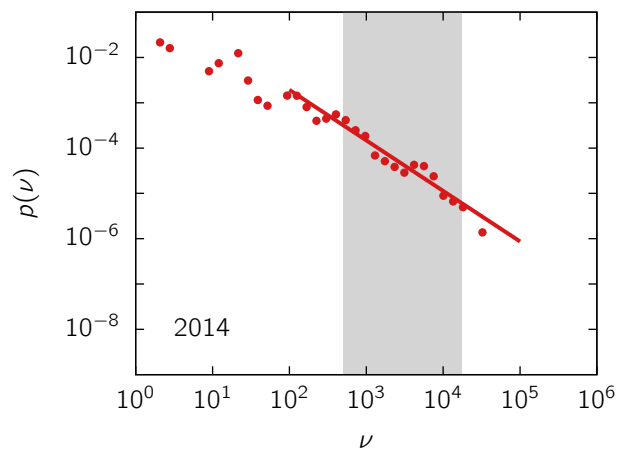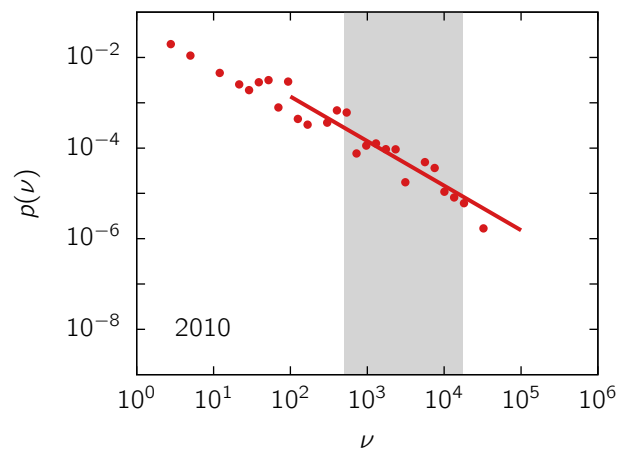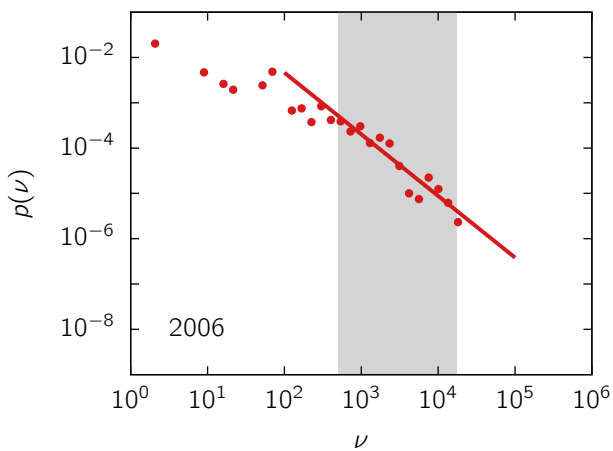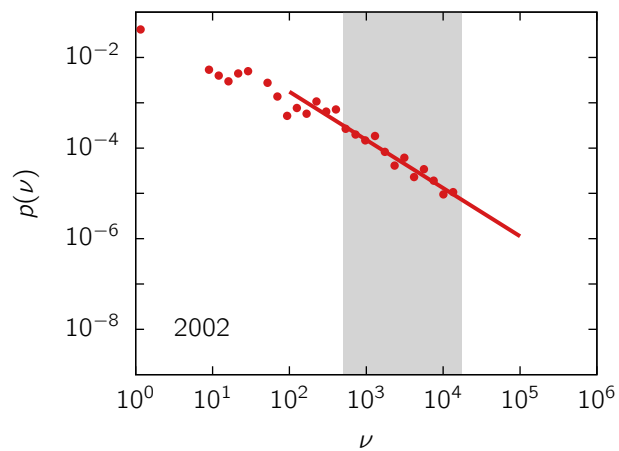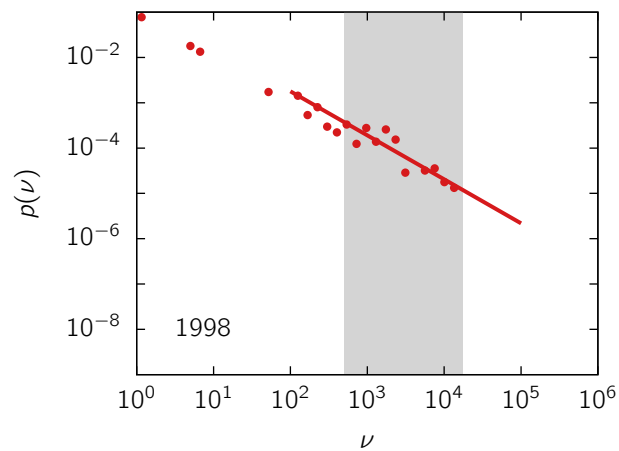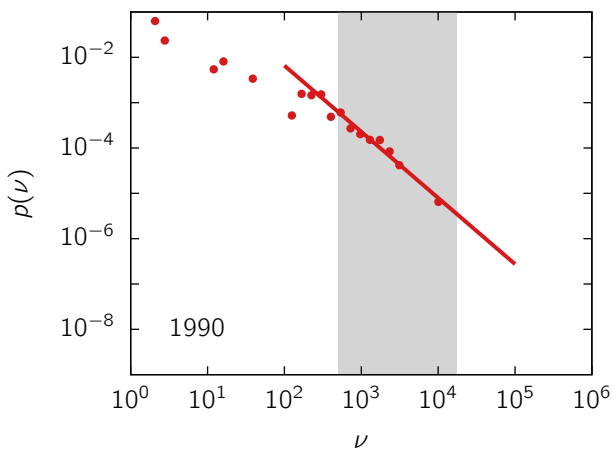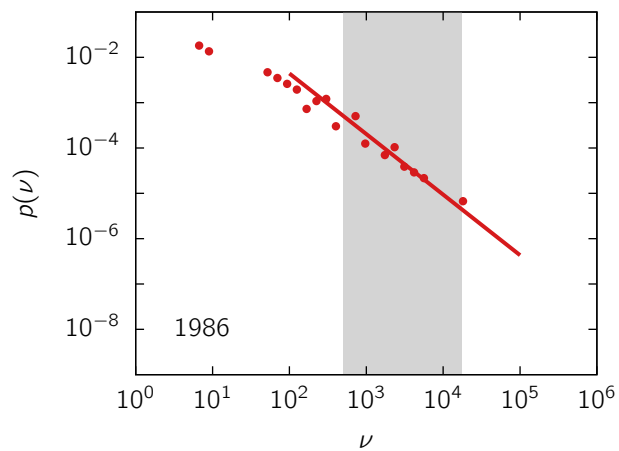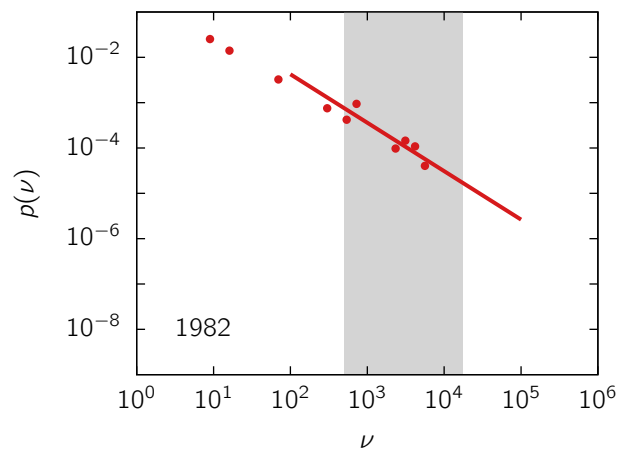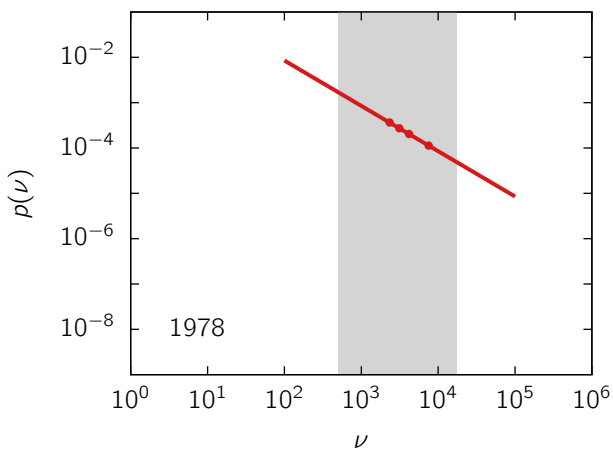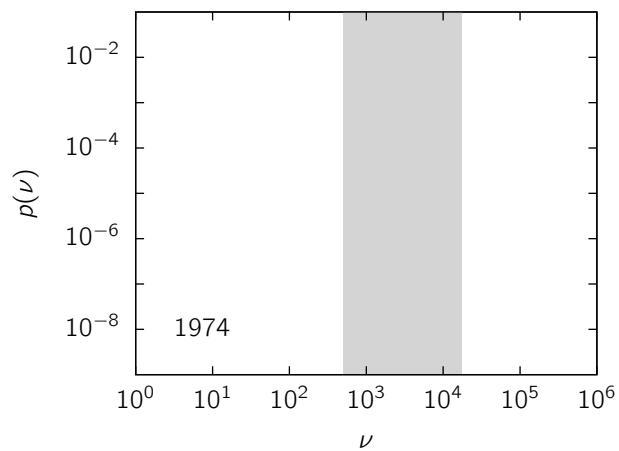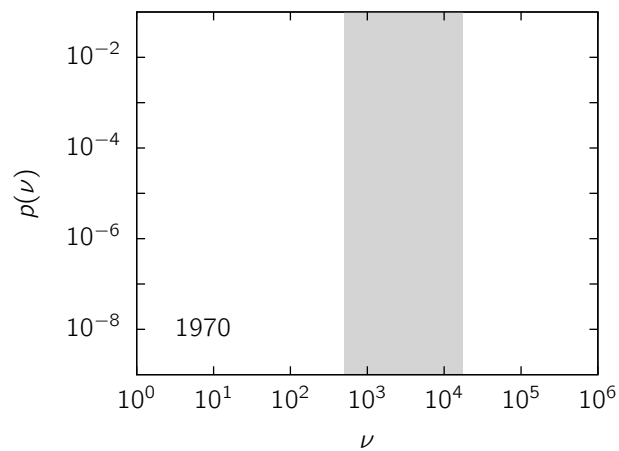

Supplement: S32 Fig — (PDF) [file pone.0137732.s038.pdf]

SC  
• Federal Deputies

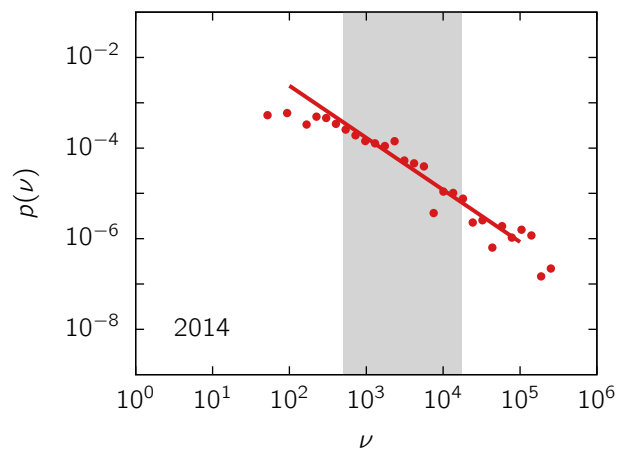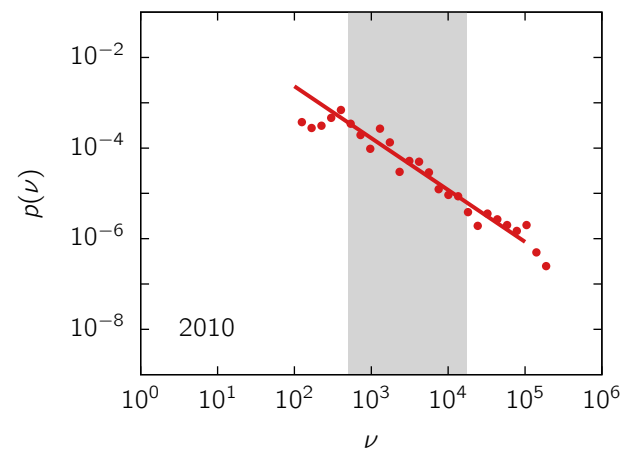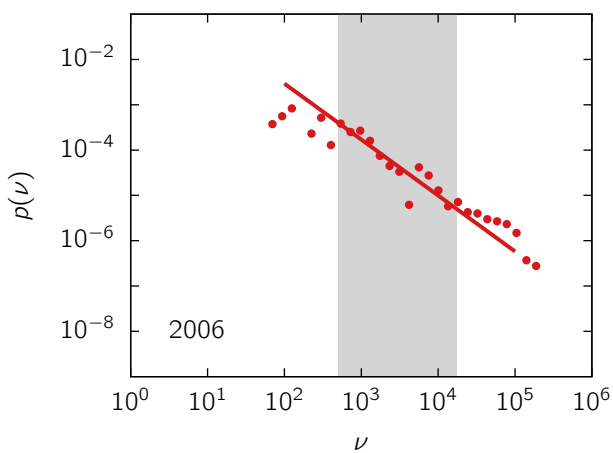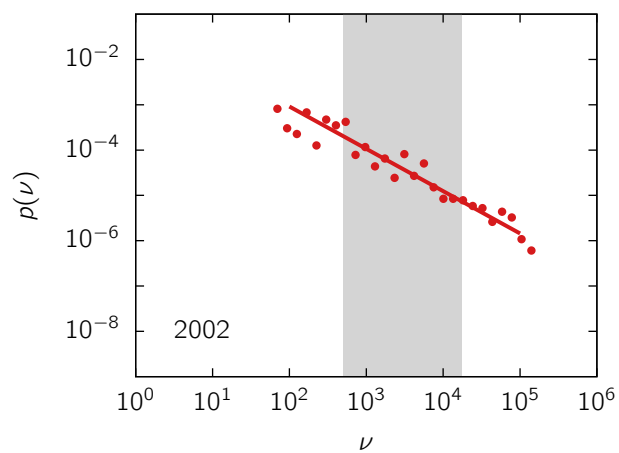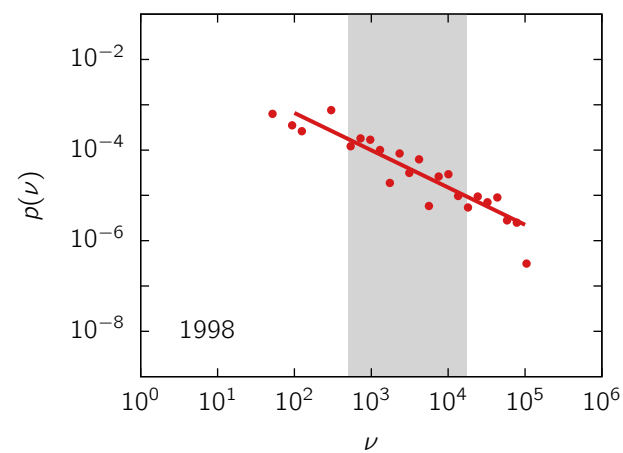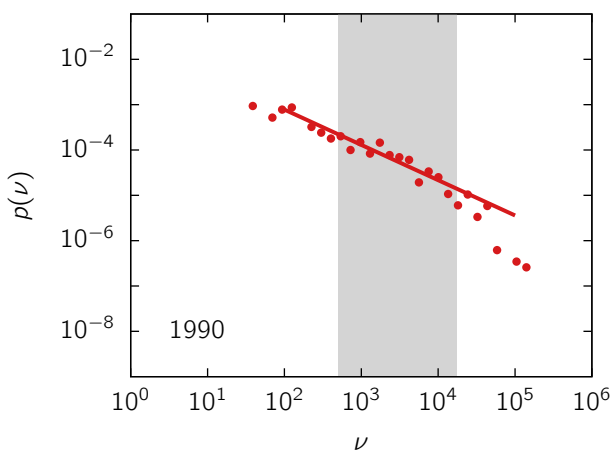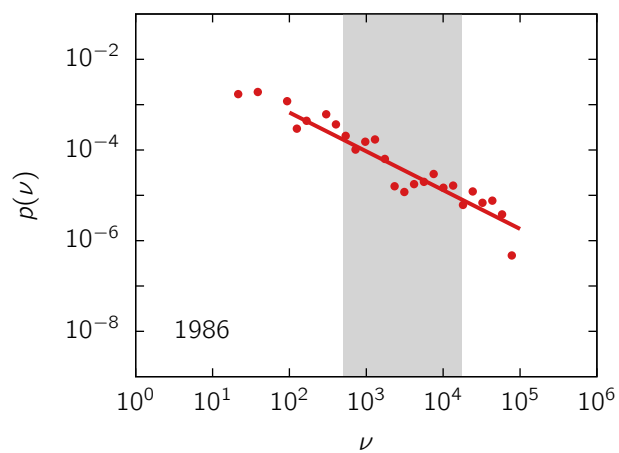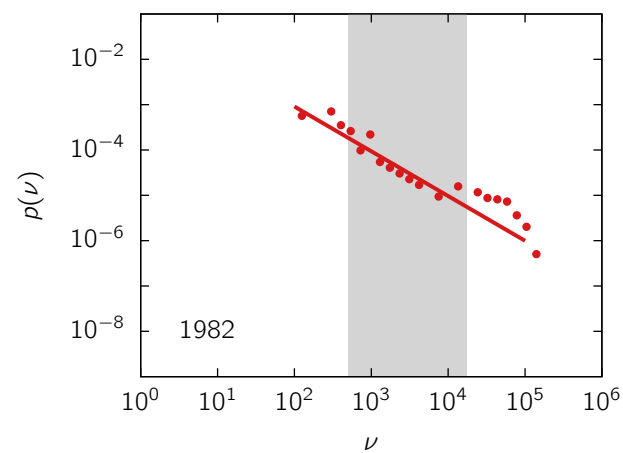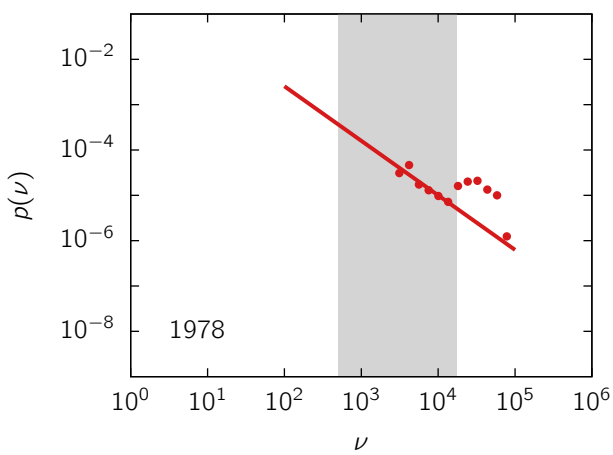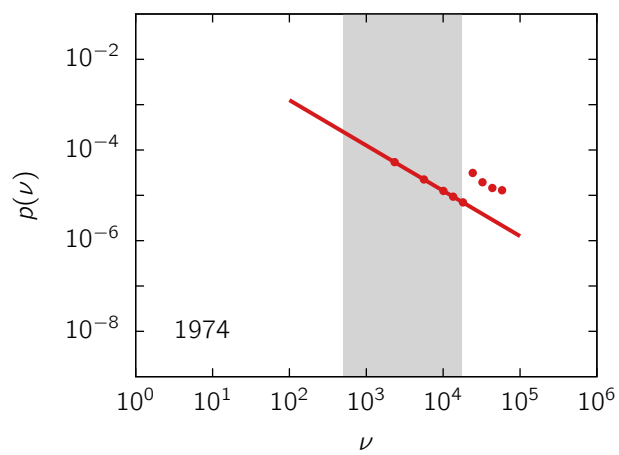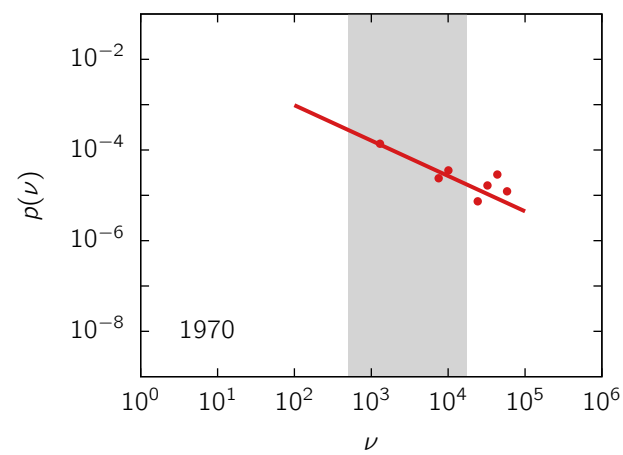

Supplement: S33 Fig — (PDF) [file pone.0137732.s039.pdf]

●

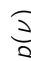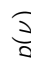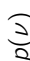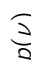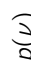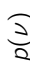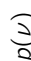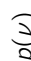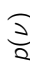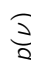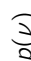

Supplement: S34 Fig — (PDF) [file pone.0137732.s040.pdf]

TO  
• Federal Deputies

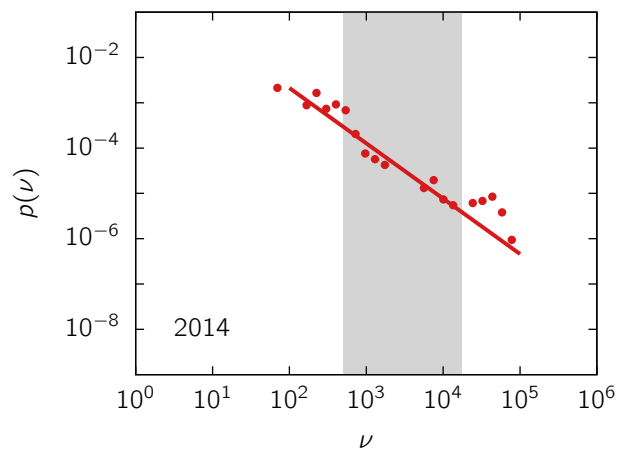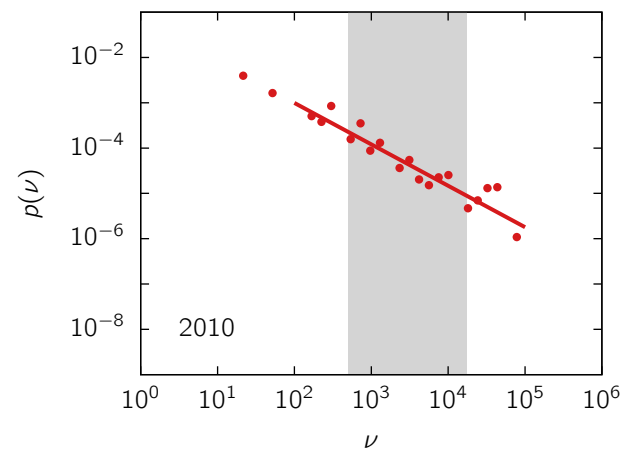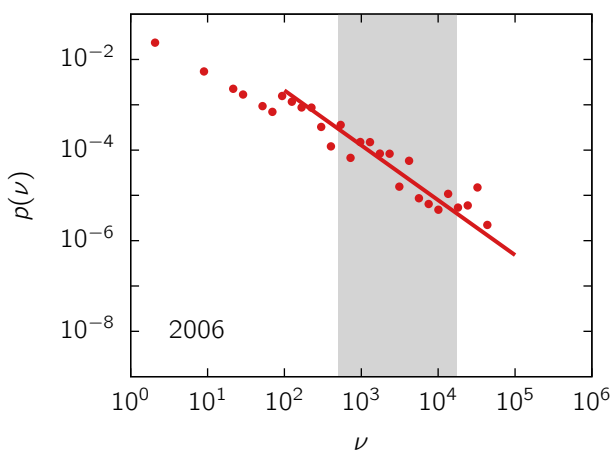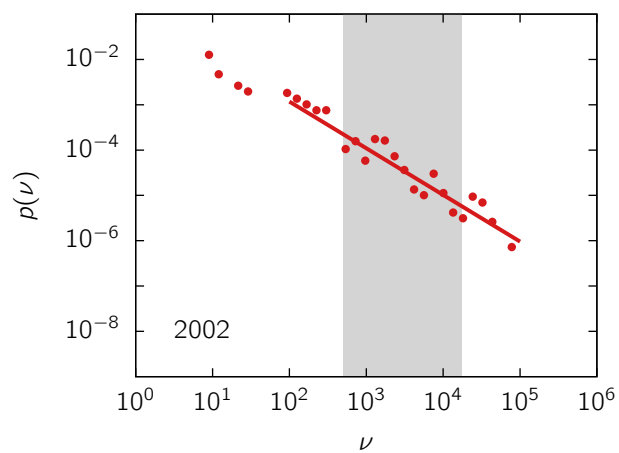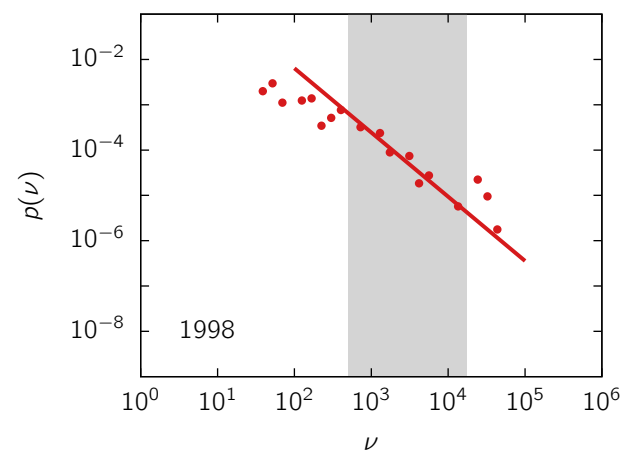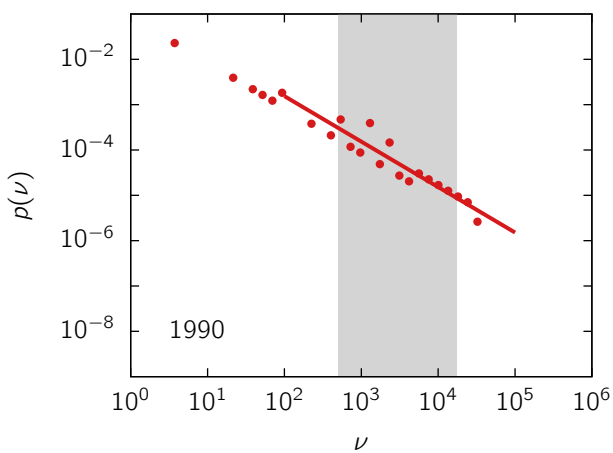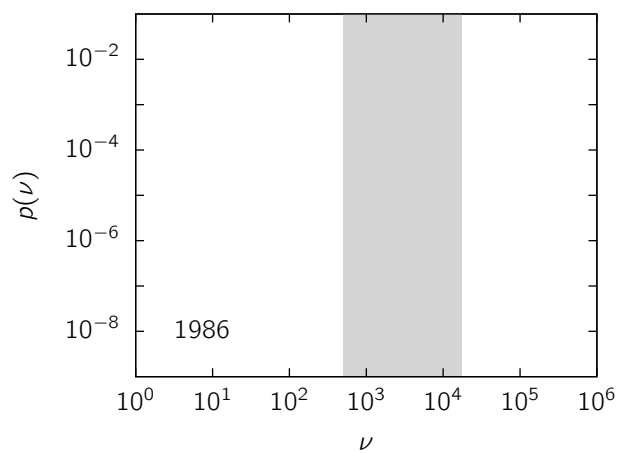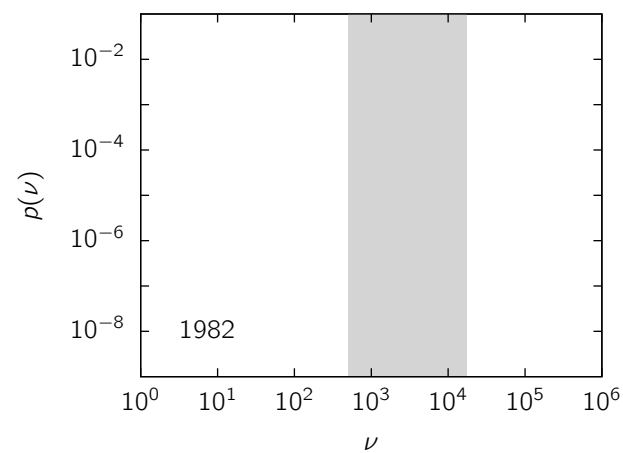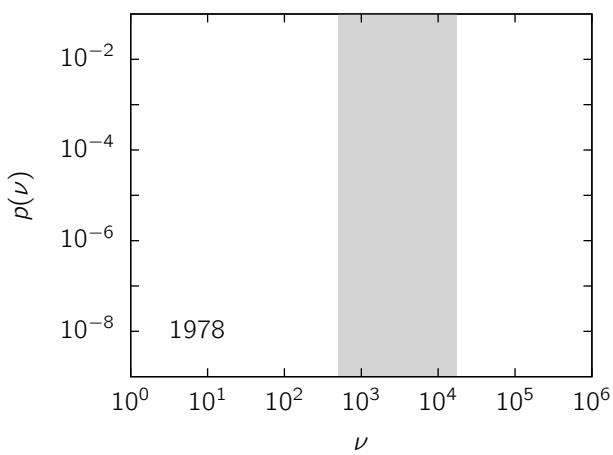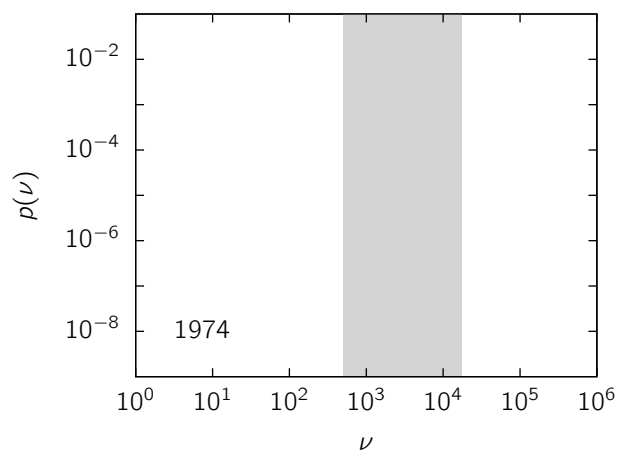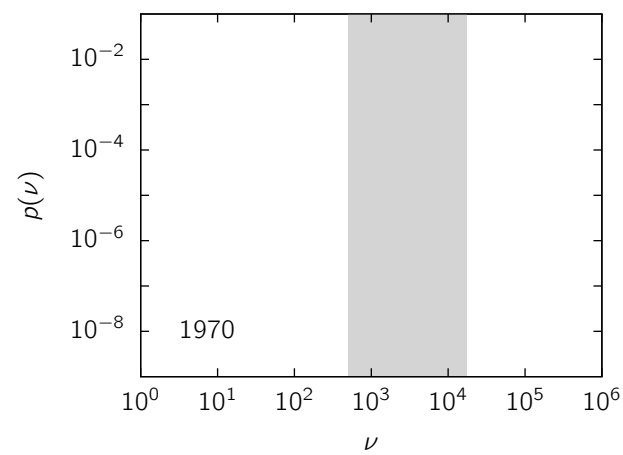

Supplement: S35 Fig — (PDF) [file pone.0137732.s041.pdf]
