# Supplementary material for: Stylized Facts in Brazilian Vote Distributions
Source: PLoS One. 2015 Sep 29;10(9):e0137732. doi: 10.1371/journal.pone.0137732 (PMC4587976; doi:10.1371/journal.pone.0137732)
Supplement: S2 Text — (DOCX) [file pone.0137732.s006.docx]

**Vote distributions for federal and state deputies.**

We show in S5-S8 Figs. the distributions of votes for federal deputies in the four most populated Brazilian states across the different calendars. For the sake of comparison, they are drawn together with those for state deputies shown previously in S1-S4 Figs.

Notice that the slopes in the shadowed region, for both kinds of deputies, are typically very similar. The uncertainty in the slope is larger for the military period, with worst statistics, however the distributions for both deputies are typically also similar in that period.
