# Supplementary material for: Stylized Facts in Brazilian Vote Distributions
Source: PLoS One. 2015 Sep 29;10(9):e0137732. doi: 10.1371/journal.pone.0137732 (PMC4587976; doi:10.1371/journal.pone.0137732)
Supplement: S6 Fig — (PDF) [file pone.0137732.s008.pdf]

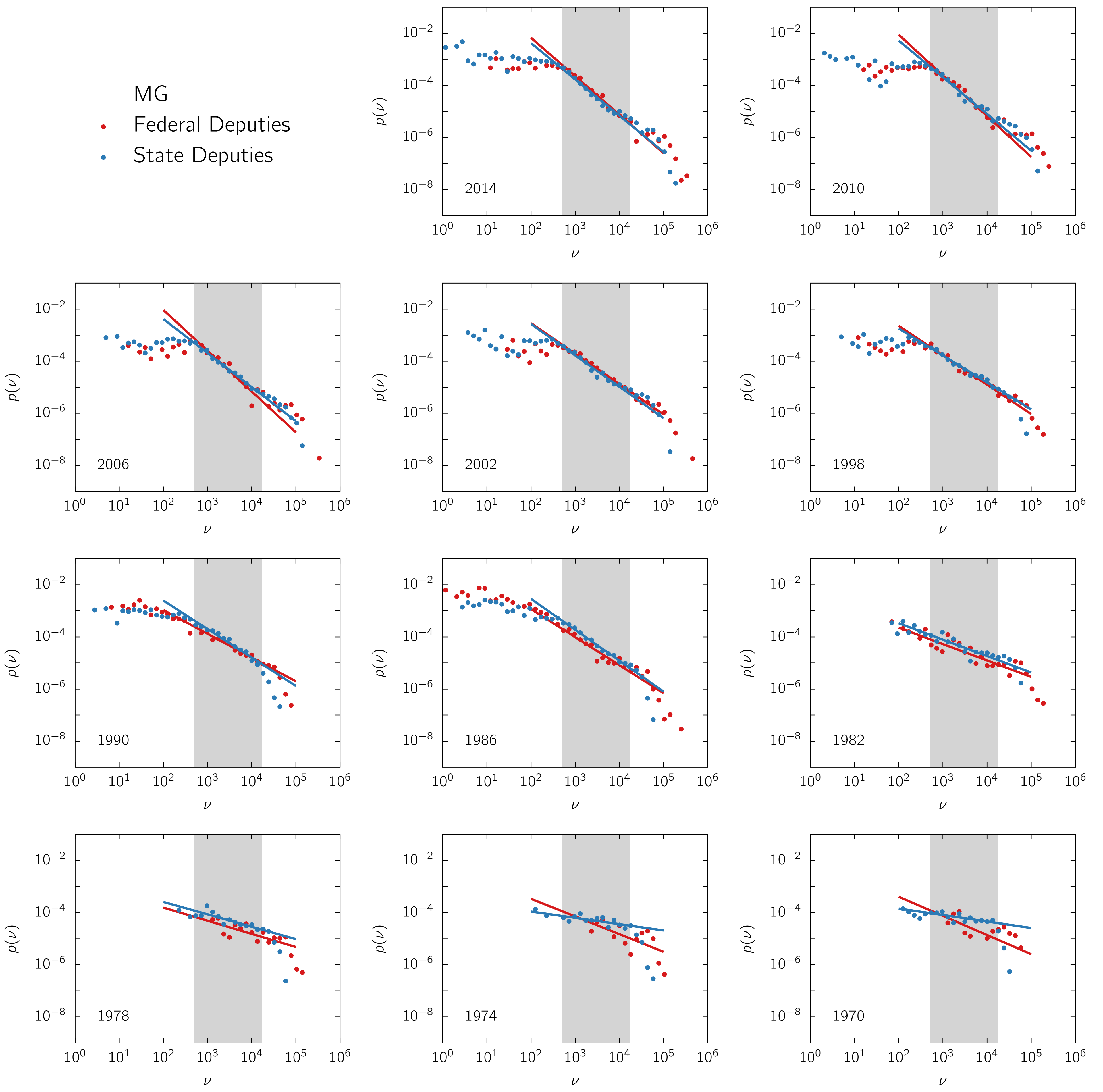
MG
Federal Deputies
State Deputies
2014
2010
2006
2002
1998
1990
1986
1982
1978
1974
1970
$p(\nu)$
ν
