# Supplementary material for: Stylized Facts in Brazilian Vote Distributions
Source: PLoS One. 2015 Sep 29;10(9):e0137732. doi: 10.1371/journal.pone.0137732 (PMC4587976; doi:10.1371/journal.pone.0137732)
Supplement: S3 Text — (DOCX) [file pone.0137732.s011.docx]

**Vote distributions for city councilors.**

We present the distributions of votes for city councilors, across the available calendars, in the cities of São Paulo (S9 Fig.) and Rio de Janeiro (S10 Fig.), which are the capitals of SP and RJ states, respectively.
