# Supplementary material for: Stylized Facts in Brazilian Vote Distributions
Source: PLoS One. 2015 Sep 29;10(9):e0137732. doi: 10.1371/journal.pone.0137732 (PMC4587976; doi:10.1371/journal.pone.0137732)
Supplement: S4 Text — (DOCX) [file pone.0137732.s014.docx]

**Vote distribution for deputies in capital cities.**

To compare the results for city councilors with those for deputies, we restricted the electorate to the corresponding state capitals. In S11 and S12 Figs., we draw together the distributions of votes for federal and state deputies, for the electorate in SP and RJ capitals, respectively.

In both cases, we also considered the complementary electorate (discarding the capital of the state) and plotted the corresponding distributions in S13 and S14 Figs.
