# Supplementary material for: Stylized Facts in Brazilian Vote Distributions
Source: PLoS One. 2015 Sep 29;10(9):e0137732. doi: 10.1371/journal.pone.0137732 (PMC4587976; doi:10.1371/journal.pone.0137732)
Supplement: S5 Text — (DOCX) [file pone.0137732.s019.docx]

**Vote distributions for federal deputies in all other states.**

Now we show the vote distributions of federal deputies in all states, except the four largest ones already shown above. The figures for calendars without available data were left blank.

To give an idea of the relative sizes, we present in S1 Table the number of votes and the number of candidates in each state in the elections of 2014.
