# Supplementary material for: Stylized Facts in Brazilian Vote Distributions
Source: PLoS One. 2015 Sep 29;10(9):e0137732. doi: 10.1371/journal.pone.0137732 (PMC4587976; doi:10.1371/journal.pone.0137732)
Supplement: S1 Table — (PDF) [file pone.0137732.s020.pdf]

State	N_v	N_c
RR	225,432	80
AP	368,061	104
AC	368,332	62
TO	670,894	47
RO	740,924	81
SE	902,788	73
MS	1,174,221	116
AL	1,283,120	100
MT	1,334,861	96
DF	1,362,160	128
RN	1,451,341	83
AM	1,560,085	79
PI	1,587,323	88
ES	1,665,277	157
PB	1,773,112	96
GO	2,824,329	95
MA	2,836,980	235
SC	3,120,297	128
PA	3,495,954	174
CE	4,002,492	195
PE	4,129,147	155
PR	5,275,880	295
RS	5,455,794	308
BA	6,010,974	312
RJ	7,026,297	953
MG	9,274,177	620
SP	18,806,745	1,318

S1 Table. Number of votes and candidates for federal deputies in the election of 2014.
